# Spectral biophysical cytometry with nanosensors reveals remodelling of immune cells in atherosclerosis

**DOI:** 10.1038/s41565-026-02236-8

**Published:** 2026-08-06

**Authors:** Cenk O. Gurdap, Dunya Aydos, Luca A. Andronico, Gábor Tóth, Tugce Ceker, John Cowgill, Irem Muge Akbulut Koyuncu, Neslihan Basak, Jaromir Mikes, Andrey S. Klymchenko, Federico Pietrocola, Petter Brodin, Ingela Lanekoff, Verda Ceylan Bitirim, Erdinc Sezgin

**Affiliations:** 1 Science for Life Laboratory, Department of Women’s and Children’s Health, Karolinska Institutet, Solna, Sweden; 2 Stem Cell Institute, Ankara University, Ankara, Turkey; 3 Ankara University Graduate School of Health Sciences, Ankara, Turkey; 4 Applied Physics, KTH Royal Institute of Technology, Stockholm, Sweden; 5 Department of Chemistry for Life Sciences, Uppsala University, Uppsala, Sweden; 6 Center of Excellence for the Chemical Mechanisms of Life, Uppsala University, Uppsala, Sweden; 7 Science for Life Laboratory, Department of Biochemistry and Biophysics, Stockholm University, Solna, Sweden; 8 Department of Cardiovascular Medicine, Ankara University School of Medicine, Ankara, Turkey; 9 Clinical Pediatrics Unit, Dept. of Women’s and Children’s Health, Karolinska Institutet, Solna, Sweden; 10 Laboratoire de Bioimagerie et Pathologies, UMR 7021 CNRS, ITI SysChem-Chimie des Systèmes Complexes, Faculté de Pharmacie, Université de Strasbourg, Illkirch, France; 11 Department of Cell and Molecular Biology, Karolinska Institute, Stockholm, Sweden; 12 Department of Immunology and Inflammation, Imperial College London, London, UK; 13 Medical Research Council, Laboratory of Medical Sciences, Imperial College Hammersmith Campus, London, UK

**Keywords:** Biosensors, Nanomedicine

## Abstract

The biophysical properties of cells determine cellular physiology. Leveraging these properties for biomedical applications demands the ability to measure multiple parameters simultaneously across millions of cells and diverse cell types. However, current technologies are limited by throughput and low dimensionality. Here we introduce spectral biophysical cytometry (SBC), a high-throughput platform that integrates environment-sensitive nanosensors with spectral flow cytometry to resolve multiparametric biophysical properties of immune cells at single-cell resolution. By using fluorescent nanosensors that report membrane order, mitochondrial potential and membrane potential, SBC enables simultaneous quantification of key cellular physical states across diverse immune cell populations. When applied to peripheral blood mononuclear cells, SBC reveals cell-type-specific biophysical heterogeneity and identifies distinct remodelling signatures associated with atherosclerosis. In particular, T-cell subsets exhibit substantial alterations in membrane order and mitochondrial depolarization, reflecting coordinated changes in lipid composition and metabolic pathways. Integration with lipidomics and transcriptomics demonstrates that the nanosensors can detect biophysical shifts that correlate with dysregulated lipid metabolism and mitochondrial function, providing mechanistic insight into immune dysfunction in disease. Importantly, SBC achieves rapid, label-efficient profiling using commercially available instrumentation, enabling scalable biomarker discovery directly from blood samples and establishing a powerful strategy for linking biophysical phenotypes to immune cell function.

## Abstract (web summary)

Membrane-permeant, environment-sensitive nanosensors enable high-throughput, multiparametric quantification of membrane order and mitochondrial potential, revealing immune-cell-specific remodelling and lipid-metabolic dysfunction in atherosclerosis.

## Main

Cells constantly remodel their biophysical properties, such as membrane order, tension, lateral diffusion, deformability and mitochondrial potential, to adapt to environmental cues, maintain homeostasis and execute specialized functions. These properties not only reflect the internal state of a cell, but also regulate fundamental processes, such as signalling, metabolism, trafficking and immune responses^1–4^. A variety of precision tools, such as atomic force microscopy and optical tweezers, have been developed to measure certain physical features, but they typically probe one cell at a time, limiting their utility in translational and clinical contexts. Among recent innovations, deformability cytometry has emerged as a powerful high-throughput platform capable of probing cell mechanical properties at rates exceeding 1,000 cells per second^5^. This exemplifies the potential for flow-based technologies to scale up physical measurements. Recent studies have demonstrated the utility of integrating a single biophysical parameter, such as membrane order^6,7^ or forces exerted through adhesion receptors^8^, with or without surface marker profiling with conventional flow cytometry to reveal previously undetected cell states. However, most current methods are: (1) custom-built; (2) constrained by their single-parameter design; (3) low sensitivity and low resolution due to the one-detector-per-fluorochrome approach; and (4) not readily compatible with immune phenotyping pipelines. This limits both cell-type specificity and the ability to integrate diverse biophysical properties. As a result, our understanding of how biophysical states vary across diverse cell types and how these features collectively contribute to health and disease remains limited.

In this study, we aimed to develop a high-throughput and multiparametric platform, spectral biophysical cytometry (SBC), based on commercially available spectral flow cytometry and environment-sensitive fluorescent probes as nanosensors of cellular health states. We used SBC to reveal the cell-type-dependent biophysical remodelling of immune cells in patients with atherosclerosis.

## Characterization of environment-sensitive nanosensors in a model system

**Fig. 1:**
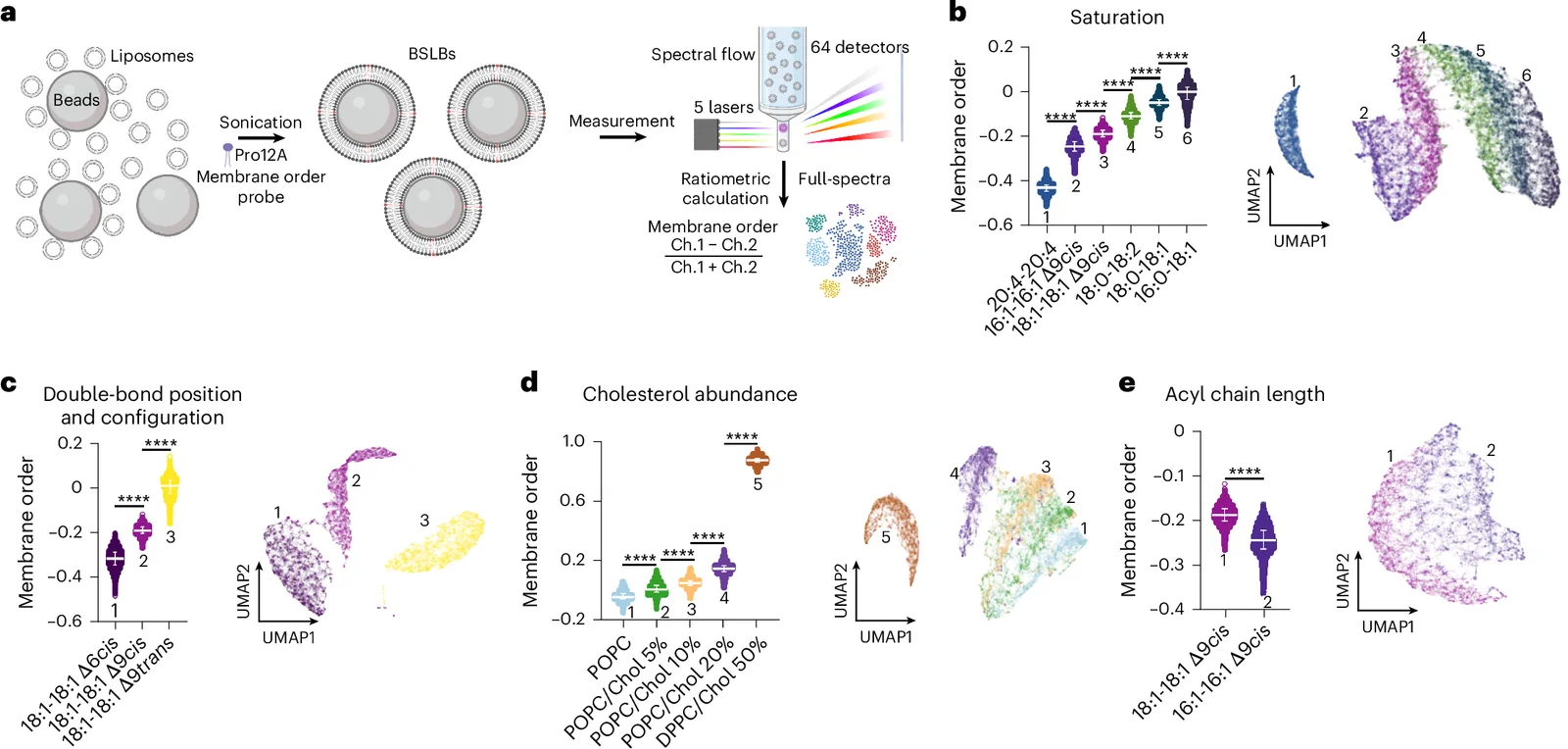
SBC applied to model membranes. **a**, Schematic overview of BSLB preparation and the SBC workflow. BSLBs serve as model membranes with defined lipid compositions, enabling systematic modulation of membrane order. **b**–**e**, Membrane order measurements in BSLBs using both ratiometric (left) and full-spectrum (right) analysis. Membrane order was systematically varied by lipid saturation (**b**), double-bond position and configuration (**c**), cholesterol abundance (**d**) and acyl chain length (**e**). White lines indicate the median and interquartile range of BSLBs. Data are representative of one experiment to compare ratiometric to full-spectrum side by side; four independent experiments are presented in the Supplementary Information. Statistical analysis was performed by one-way non-parametric ANOVA, except lipid tail length (tested by two-tailed Mann–Whitney): ****P < 0.0001. Each sample includes measurements from around 10,000 beads. DPPC, 1,2-dipalmitoyl-sn-glycero-3-phosphocholine; Chol, cholesterol. Illustrations in **a** created in BioRender; Andronico, L. https://biorender.com/ln4y9re (2026). Source data

First, to demonstrate the sensitivity and robustness of our pipeline, we utilized a model membrane system consisting of bead-supported lipid bilayers (BSLBs), created by coating cell-sized 5-μm silica beads with synthetic liposomes (Fig. 1a). BSLBs were labelled with either Pro12A or NR12S, two environment-sensitive molecular probes for biomembranes, whose emission spectra shift according to membrane packing^9–11^. BSLBs were analysed on a spectral flow cytometer equipped with five lasers and 64 narrow-band detectors, enabling high-dimensional data acquisition. Control experiments did not reveal any notable autofluorescence from pure beads, coated but unlabelled beads, or uncoated labelled beads, confirming that the signal originated specifically from coated and labelled BSLBs (Supplementary Fig. 1). We then tested the sensitivity of the probes by varying the lipid compositions in BSLBs that differ in saturation, double-bond position and configuration, cholesterol abundance, and acyl chain length. Pro12A outperformed NR12S across these categories, demonstrating higher sensitivity (Supplementary Fig. 2a–f). Hence, we selected Pro12A for subsequent high-dimensional analyses. Traditionally, membrane order is quantified using ratiometric measurements based on two specific emission channels (Fig. 1b–e, left). In contrast, applying Uniform Manifold Approximation and Projection (UMAP) to the full spectral profile improved separation between different lipid compositions across all tested categories (Fig. 1b–e, right). Quantitative comparisons using supervised learning further demonstrated that full-spectrum analysis significantly enhances classification accuracy relative to two-channel ratiometric approach (Supplementary Fig. 2g). These results suggest that incorporating the full spectral signature of the probe captures additional biophysical information, thereby offering superior resolution compared with conventional ratiometric methods.

## Multiparametric biophysical remodelling of cell lines upon perturbations

Following validation of the pipeline in a model membrane system, we applied SBC to the CEM cell line (T lymphoblast, acute lymphoblastic leukaemia). We began by optimizing the concentration of Pro12A to avoid self-quenching while maintaining a strong fluorescence signal (Supplementary Fig. 3a). Notably, we found that Pro12A also reliably provided a real-time readout of cell viability that is equivalent to conventional Live/Dead staining (Supplementary Fig. 3b–j), without requiring additional staining or wash steps. In dead cells, Pro12A is rapidly internalized and reports low membrane order, reflecting the disordered nature of intracellular organelles. Because the probe is applied immediately before acquisition, it detects cells that undergo death during the interval between conventional viability staining and cytometric measurement, a limitation inherent to amine-reactive dyes. This integrated viability assessment could reduce cell loss during sample preparation, simplifies the experimental workflow, and minimizes viability-related artefacts in time-sensitive sample measurements. This suggests that Pro12A could potentially obviate traditional viability dyes during biophysical measurements, which is especially advantageous when working with multiple probes and limited spectral bandwidth. Nevertheless, to ensure robustness, we included conventional viability staining alongside membrane order probe in all experiments, except in measurements using membrane potential dye due to its broad red spectrum. In these cases, the combination of membrane order and light scatter (since dead cells display distinct size distributions) provided a reliable means of discriminating live from dead cells. Importantly, all experiments were performed under optimized culturing conditions, and our analyses of blood samples consistently started with the highest possible viability.

**Fig. 2:**
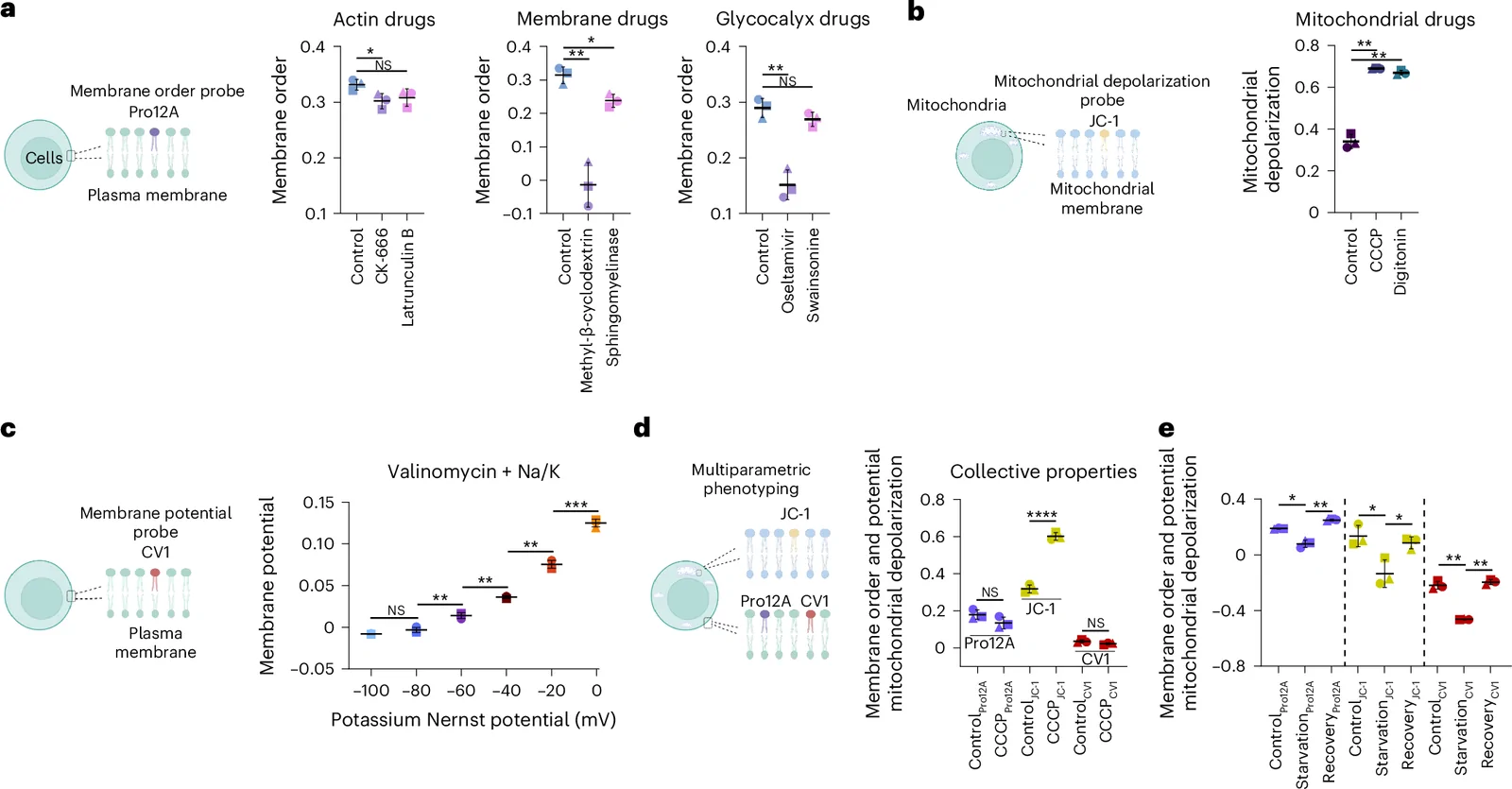
SBC applied to cell lines. **a**, Membrane order of CEM T cells after treatment with drugs targeting the actin cytoskeleton, membrane and glycocalyx, demonstrating SBC’s ability to resolve drug-induced changes at the single-cell level, using the Pro12A probe. **b**, Quantification of mitochondrial depolarization in CEM T cells following treatment with mitochondrial drugs, detected using the JC-1 probe. **c**, Changes in plasma membrane potential of CEM T cells under varying extracellular KCl concentrations and corresponding potassium Nernst potential, measured with the CV1 probe. **d**, Multiparametric profiling of CEM T cells treated with CCCP, showing simultaneous measurement of membrane order (Pro12A), mitochondrial depolarization (JC-1) and membrane potential (CV1). **e**, Multiparametric profiling of CEM T cells in starvation-recovery experiments, highlighting dynamic and coordinated changes across all three parameters. Data are representative of three independent experiments. Black lines indicate the mean ± s.d. Statistical analysis perfomed by unpaired *t*-test with Welch’s correction: ****P < 0.0001; ***P < 0.001; **P < 0.01; *P < 0.05; NS, not significant. Each data point includes measurements from at least 5,000 cells. Schematics in **a**–**d** created in BioRender: **a**, Andronico, L. https://biorender.com/go5919m (2026); **b**, https://biorender.com/twj9t3p (2026); **c**, https://biorender.com/2snu8ri (2026); **d**, https://biorender.com/zlcea4e (2026). Source data

Under optimized staining conditions, we treated CEM cells with a panel of compounds selected to perturb distinct cellular structures known to influence membrane organization (Fig. 2a). We selected drugs that can act on actin cytoskeleton, membrane lipids and glycocalyx. Although all these structures are crucial for membrane integrity and function, it is not clear how they contribute to distinct biophysical properties. Actin cytoskeleton drugs (CK-666, an Arp2/3 inhibitor, and latrunculin B, which sequesters actin monomers) were used to alter cortical actin dynamics that directly support membrane structure. Plasma membrane lipid composition was perturbed by methyl-β-cyclodextrin (which extracts cholesterol) and sphingomyelinase (which hydrolyses sphingomyelin) which target two important lipids for membrane properties. To probe the role of the glycocalyx, we included oseltamivir (a neuraminidase inhibitor that preserves sialylated glycans) and swainsonine (an α-mannosidase inhibitor that interferes with *N*-glycan maturation), thereby altering surface glycan composition. Across all drug treatments, we observed a consistent decrease in membrane order, showing the impact of these structural elements on the physical properties of membranes and indicating the sensitivity of Pro12A to biophysical perturbations induced by various cellular structures. To support our findings, we included representative spectral images, spectral plots, spectral flow cytometry data and full datasets from biological replicates (Supplementary Figs. 4, 5 and 6).

To enable multiparametric biophysical phenotyping and uncover underexplored interparameter correlations, we integrated two additional environment-sensitive probes, JC-1 (mitochondrial depolarization) and CV1 (membrane potential)^12^. Using JC-1, we monitored mitochondrial depolarization in cells treated with carbonyl cyanide 3-chlorophenylhydrazone (CCCP, a protonophore uncoupler) or digitonin (which permeabilizes plasma membrane without notable damage to intracellular membranes and disrupts ionic homeostasis) (Fig. 2b and Supplementary Fig. 7). JC-1 accumulates in mitochondria in a potential-dependent manner: at high membrane potential (lower depolarization), it forms red-fluorescent aggregates, and at low potential (higher depolarization), it remains as green-fluorescent monomers. As expected, depolarized cells displayed an increased JC-1 monomer signal. For membrane potential analysis, cells were modulated with valinomycin, a potassium ionophore enabling the selective transport of potassium ions (K^+^) in combination with varying extracellular K^+^/Na^+^ ratios to calibrate CV1 responses according to the Nernst equation. We observed a strong correlation between the theoretically calculated membrane potential and the ratiometric fluorescence signal, validating CV1 as a reporter of membrane potential in non-excitable cells (Fig. 2c and Supplementary Fig. 8). Next, we combined Pro12A, JC-1 and CV1 to perform high-throughput and multiparametric biophysical profiling in single cells (Fig. 2d and Supplementary Fig. 9a–d). Cells treated with CCCP were simultaneously assessed for changes in membrane order, mitochondrial depolarization and membrane potential (Supplementary Fig. 9e,f). Notably, CCCP not only affected mitochondrial depolarization, as previously described, but also slightly reduced membrane order, illustrating the power of multiparametric analysis to reveal off-target or secondary effects. Full-spectrum analysis with UMAP further enabled separation of treated and control populations based on their combined biophysical signatures (Supplementary Fig. 9g). To extend this approach to a physiological context, we created a serum starvation model to investigate how nutrient availability alters cellular biophysical states (Supplementary Fig. 9h). After 24 h of fetal bovine serum (FBS) deprivation, cells exhibited a decrease in membrane order, membrane potential and mitochondrial depolarization (Fig. 2e). Interestingly, these phenotypes were reversed upon re-exposure to FBS for 48 h, with cells returning to a state resembling the control condition. Together, these results demonstrate the feasibility and value of multiparametric SBC for capturing dynamic biophysical changes at the single-cell level in both drug-treated and physiologically perturbed systems.

## Multiparametric biophysical remodelling of blood cells upon perturbations

**Fig. 3:**
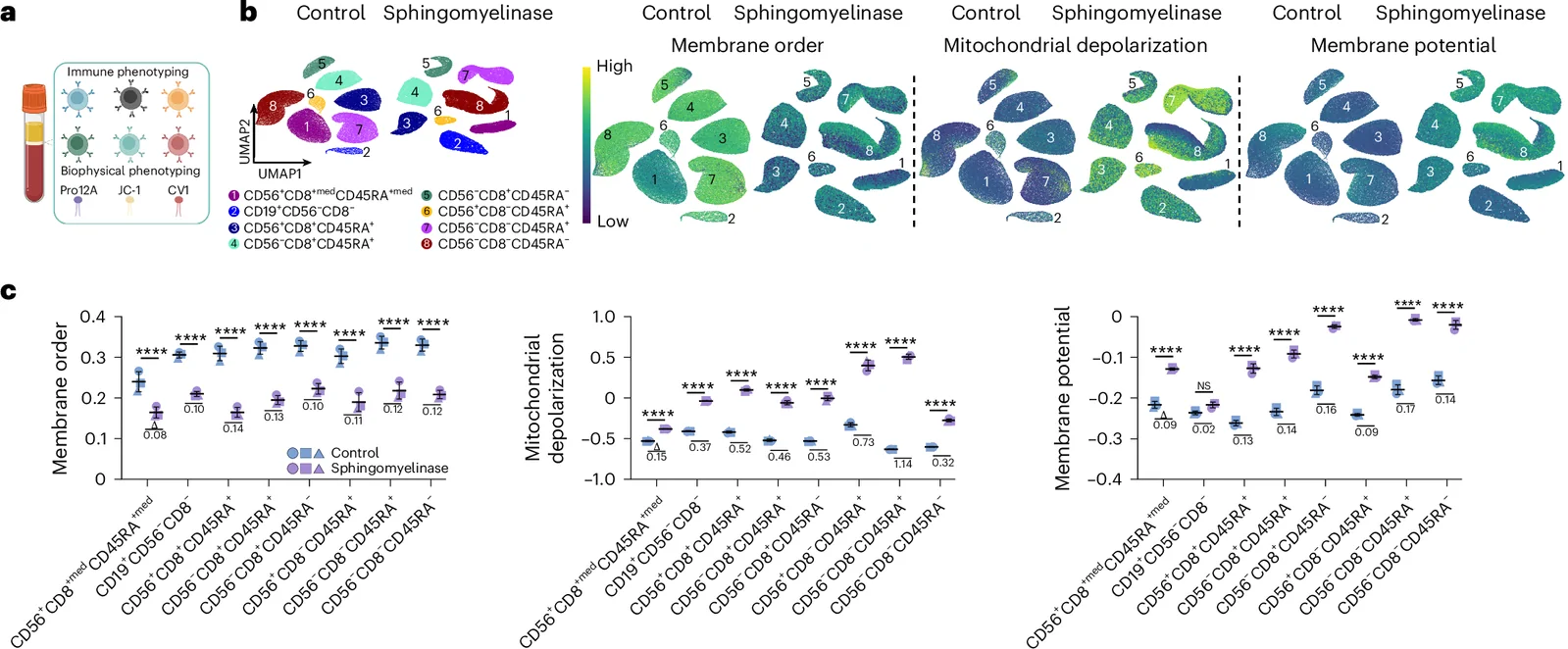
SBC applied to PBMCs. **a**, Scheme of integrating antibody-based immunophenotyping with SBC in human PBMCs, enabling simultaneous resolution of immune cell identity and biophysical state. **b**, Multiparametric phenotyping in PBMCs untreated or treated with sphingomyelinase, showing cell-type-dependent changes in membrane order, mitochondrial potential and membrane potential, where brighter colours indicate more ordered membranes, higher depolarization of mitochondria and higher membrane potential. Numbers on the UMAP plots indicate the annotated immune cell types as determined by antibody-based staining. **c**, Comparison of the three biophysical parameters across all PBMC subsets in control versus sphingomyelinase-treated samples, demonstrating cell-type-dependent responses to perturbation. PBMC data are representative of three independent experiments. Black lines indicate the mean ± s.d. Statistical analysis was performed by two-way ANOVA: ****P < 0.0001. Δ indicates mean difference. Each data point includes measurements from at least 15,000 cells. Illustrations in **a** created in BioRender; Andronico, L. https://biorender.com/4vi8i9z (2026). Source data

After establishing a robust multiparametric system for biophysical characterization, we extended the approach to human blood-derived immune cells. Immune cells continuously circulate throughout the body and can reflect systemic physiological and pathological states, making them attractive candidates for early disease biomarkers. A major advantage of SBC, due to the flexibility of spectral flow cytometry, is the ability to combine biophysical probes with immunophenotyping markers with high cell-type granularity. This enables simultaneous analysis of cell identity and biophysical state at single-cell resolution. Our goal was to integrate immune phenotyping with biophysical profiling to assess how different immune cell types exhibit distinct biophysical signatures in health versus disease. We first focused on perturbed immune cells to assess how different cell types respond to membrane-related challenges and to evaluate the sensitivity of our system (Fig. 3a). By applying well-defined cellular perturbations, we aimed to determine whether SBC can detect biophysical changes at the single-cell level in a cell-type-dependent manner. This approach provides a benchmark for the performance of the method before extending it to clinical or more heterogeneous samples. As a disease-relevant model, we first analysed drug-treated peripheral blood mononuclear cells (PBMCs) using all three biophysical probes, Pro12A, JC-1 and CV1, either individually or in combination, together with immunophenotyping panels. Our analysis strategy was to generate UMAP projections of manual gating based on antibodies for cell-type clusters and then overlay biophysical properties for each cluster with single-cell resolution. To assess membrane order, we treated PBMCs with sphingomyelinase and stained with Pro12A (Supplementary Fig. 10). In this set-up, a ten-colour panel (antibodies + Live/Dead dye) was used alongside Pro12A, enabling assessment of membrane order across 16 immune cell subsets. Next, to evaluate mitochondrial depolarization, we treated cells with CCCP and stained with JC-1. Here, a nine-colour panel was combined with JC-1 to quantify mitochondrial depolarization changes across 15 immune cell types (Supplementary Fig. 11). For membrane potential measurements, PBMCs were treated with KCl and stained with CV1. A four-colour panel was used with CV1, enabling resolution of eight immune cell types and their membrane potential profiles (Supplementary Fig. 12). We further demonstrated dual-probe applications, combining Pro12A and JC-1 in CCCP-treated cells (Supplementary Figs. 13 and 14), and finally applied the full triple-probe set-up (Pro12A, JC-1, and CV1) in drug-treated PBMCs to perform multiparametric biophysical phenotyping (Fig. 3b and Supplementary Figs. 15 and 16). We used sphingomyelinase as a model drug that targets sphingomyelin (hydrolysis of sphingomyelin to ceramide), which is one of the main lipids regulating membrane integrity and order^13^. It was shown by lipidomics that there are different levels of sphingomyelin in different immune cell types^14^. Therefore, we treated PBMCs with sphingomyelinase to test how modulating membrane sphingomyelin content alters biophysical properties. Sphingomyelinase treatment induced changes across all three measured parameters; however, the magnitude of these parameters varied between immune cell types (Fig. 3c). In addition, we observed baseline differences in biophysical properties across untreated immune cell populations (Supplementary Fig. 16c,f,i), highlighting intrinsic heterogeneity between immune cell types. These findings are consistent with previous lipidomic studies^14^, which have reported substantial diversity in lipid composition across immune subsets, probably underlying differences in membrane composition and responsiveness to lipid-modifying interventions. Together, these results establish SBC as a high-throughput and multiparametric approach for profiling biophysical states of diverse immune cell populations under perturbation, opening new avenues for immune biomarker discovery and disease monitoring.

## Biophysical remodelling in atherosclerosis

**Fig. 4:**
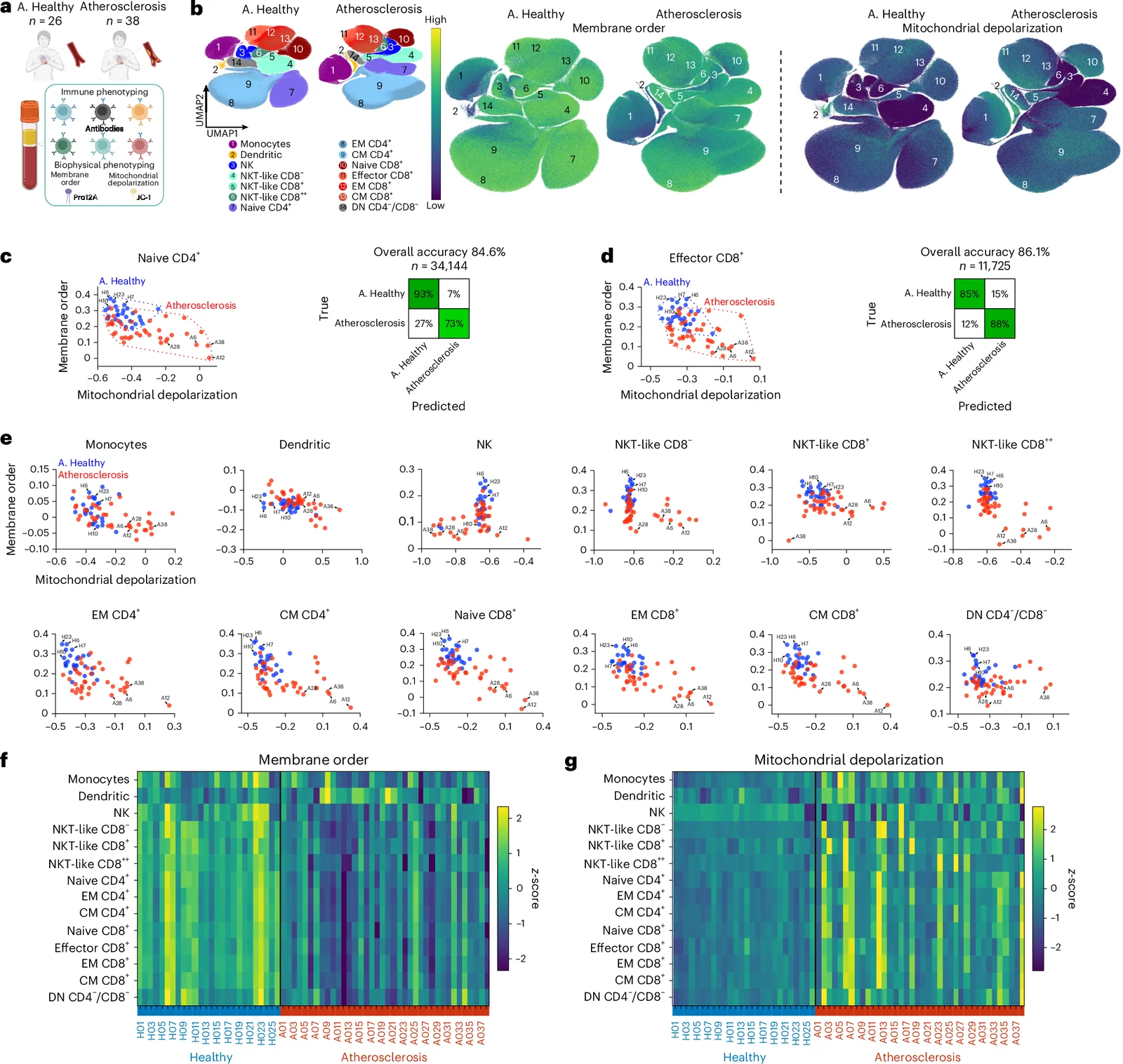
Multiparametric biophysical phenotyping of atherosclerosis. **a**, Integration of antibody-based immunophenotyping with SBC in PBMCs from apparently healthy donors (*n* = 26) and patients with atherosclerosis (*n* = 38). **b**, Comparison of single-cell membrane order (Pro12A) and mitochondrial depolarization (JC-1) across major immune cell types in both groups, revealing cell-type-dependent biophysical differences, where brighter colours indicate higher-ordered membranes and higher depolarization of mitochondria. **c**, Biophysical profiling of naive CD4^+^ T cells. Shown are median of membrane order versus mitochondrial depolarization by ratiometric analysis (left) and full-spectrum supervised classification (right), illustrating multiparametric phenotyping and its predictive power. Donor numbers on the left plot indicate the selected donors later used for omics studies. **d**, Equivalent analyses for effector CD8^+^ T cells. **e**, Extended 2D biophysical profiling across additional immune cell types, showing broad immune heterogeneity between healthy and atherosclerosis donors. Colours indicate donor medians for that specific cell type. **f**, Pooled *z*-score heatmap of biophysical remodelling for membrane order across immune cell types, showing how far each individual deviates from the overall population centre and reflecting the heterogeneity between donors. **g**, Equivalent analyses for mitochondrial depolarization. H6, H7, H10, H23, A6, A12, A28 and A38 annotations in the graphs indicate selected individuals for omics studies in Fig. 5. Illustrations in **a** created in BioRender; Andronico, L. https://biorender.com/692roa1 (2026). Source data

Following validation of SBC on drug-treated PBMCs, we integrated immune phenotyping with biophysical profiling to differentiate healthy individuals from those with atherosclerosis (Fig. 4a). Atherosclerosis is a multifactorial disease marked by endothelial dysfunction, dysregulated lipid metabolism, chronic inflammation and sustained immune activation^15^. Circulating immune cells are central to both the initiation and progression of atherosclerotic lesions in a cell-type-dependent manner^16^, yet their biophysical phenotypes remain poorly characterized, largely due to a lack of high-throughput tools capable of resolving immune cell heterogeneity at the single-cell level. To address this, we applied SBC to PBMCs using two biophysical probes, Pro12A for membrane order and JC-1 for mitochondrial depolarization, combined with immune markers to capture biophysical remodeling across 14 immune cell types in health and disease. Applying the gating strategy established in drug-treated PBMCs (Supplementary Fig. 13), we achieved single-cell resolution profiling of biophysical and metabolic states across immune subsets (Fig. 4b). Before comparing conditions, we characterized the baseline biophysical heterogeneity across immune cell types within the healthy donor cohort (Supplementary Fig. 17). Membrane order and mitochondrial depolarization both exhibited pronounced cell-type-dependent differences, demonstrating that biophysical states are inherently heterogeneous across the immune system rather than uniformly distributed. Pairwise comparisons confirmed that most cell types carry statistically distinct biophysical profiles with consistent effect sizes across donors (Supplementary Fig. 17a,b,d,e). Critically, variance decomposition analysis showed that cell identity accounted for the large fraction of total variability in both membrane order (84.8%) and mitochondrial depolarization (83.4%), with interindividual differences contributing substantially less (10.6% and 5.2%, respectively; Supplementary Fig. 17c,f). Together, these results establish a cell-type-intrinsic baseline landscape of immune cell biophysics in healthy individuals, providing a necessary reference for interpreting disease-associated alterations in a cell-type-resolved manner. We then compared healthy donors and atherosclerotic individuals across all 14 immune cell types. Two-way mixed ANOVA revealed significant group and cell-type interactions for both membrane order and mitochondrial depolarization, confirming that disease-associated biophysical remodelling is cell-type-dependent rather than uniform across the immune compartment (Supplementary Fig. 18). Per-cell-type comparisons identified T cells, including CD4^+^ and CD8^+^ subsets and their respective subtypes, as carrying the most pronounced biophysical changes in both parameters (Supplementary Fig. 18a–d). To visualize interindividual differences, we plotted median membrane order and mitochondrial depolarization for naive CD4^+^ and effector CD8^+^ T cells in a two-dimensional (2D) feature space, which indicated a shift in the distribution between healthy and atherosclerotic individuals (Fig. 4c,d, left). Supervised machine learning on full spectral profiles further confirmed the high predictive accuracy for distinguishing the two conditions in both populations (Fig. 4c,d, right). We note, however, that our cohort (*n* = 26 healthy, *n* = 38 atherosclerosis) was designed for proof-of-concept discovery at the single-cell level and is not powered for subject-level diagnostic modelling; accordingly, we restrict interpretation to cell-level predictive performance.

Extending the analysis across all measured immune subsets, 2D feature plots (Fig. 4e) and per-subset classification results (Supplementary Fig. 18e) confirmed cell-type-dependent biophysical alterations throughout the immune compartment. To examine interindividual variability better, we visualized donor-specific deviations in membrane order and mitochondrial depolarization across all 14 populations (Fig. 4f,g). While consistent cell-type-level trends were apparent, the magnitude and pattern of remodelling varied between individuals, revealing distinct donor-specific biophysical signatures and remodelling. Together, these findings highlight the cell and donor-dependent nature of disease-associated biophysical remodelling and underscore the value of SBC in uncovering both shared and individual-specific immune biomarkers.

## Integrating biophysical remodelling with lipidomics and transcriptomics

**Fig. 5:**
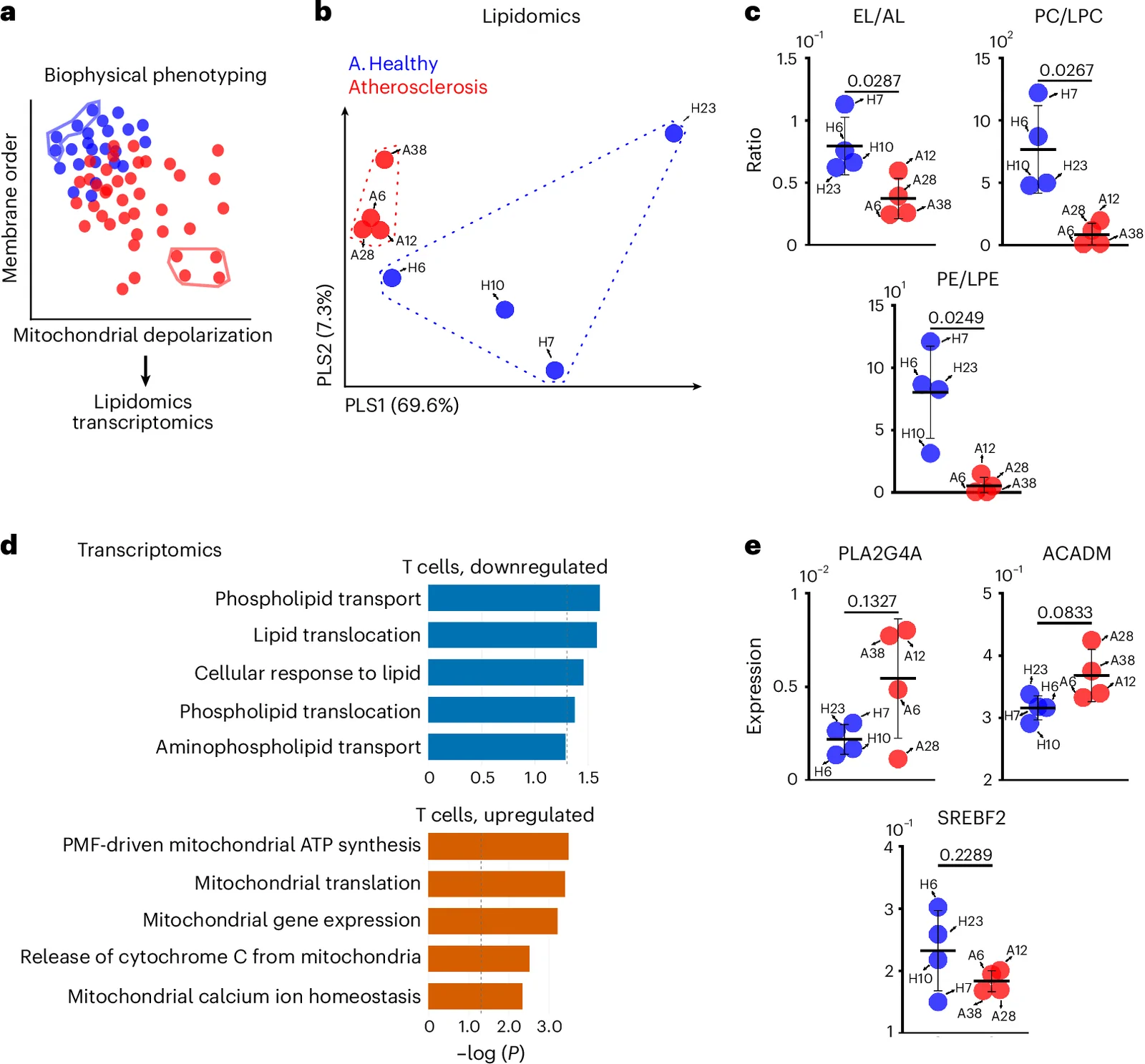
Integration of biophysics with multi-omics. **a**, Schematic representation of subset of donors (*n* = 8, both healthy and atherosclerosis, annotated individuals) selected based on divergent biophysical signatures for subsequent multi-omics integration. **b**, Lipidomic profiling of T cells from the same donors by mass spectrometry using PLS regression identifies key lipid features that discriminate between conditions. **c**, Ratios of lipid classes (EL/AL, ether/acyl phospholipids; PC/LPC, phosphatidylcholine/lysophosphatidylcholine; PE/LPE, phosphatidylethanolamine/lysophosphatidylethanolamine), demonstrating systematic remodelling in atherosclerosis. **d**, Pathway-level analysis of scRNA-seq data from the same T cells, focusing on lipid- and mitochondria-related processes. **e**, Expression of genes involved in lipid synthesis across groups, shown as average log-normalized counts from scRNA-seq. Black lines indicate the mean ± s.d. across donors. Statistical significance was assessed by two-tailed Welch’s *t*-test. The numbers shown above the graphs indicate the *P* values. PMF, proton-motive force. Source data

To investigate the underlying mechanisms behind biophysical remodelling in T cells of patients with atherosclerosis, we selected four individuals from each group, healthy and atherosclerotic, who represented the clear biophysical phenotypes (Fig. 5a). We focused our downstream analysis on T cells, which not only showed substantial biophysical remodelling but are also known as critical mediators in atherosclerotic disease progression^17^. Mass-spectrometry-based lipidomics of T cells from eight donors revealed global alterations in lipid composition in individuals with atherosclerosis (Fig. 5b and Supplementary Fig. 19). Notably, the ether:acyl phospholipid and phospholipid:lysophospholipid ratios were significantly altered (Fig. 5c). Both ether lipids and lysolipids are known to modulate membrane order^18,19^. Therefore, a notable depletion of ether lipids and increase in lysolipids explain the biophysical anomalies observed in the membranes of T cells from patients with atherosclerosis. These changes in ether and lysolipids might also suggest increased oxidative stress susceptibility, impaired immune signalling and enhanced release of inflammatory mediators^18,20,21^. In addition, single-cell RNA sequencing (scRNA-seq) of T cells from the same individuals revealed consistent transcriptional changes across key pathways; genes involved in lipid metabolism^22,23^ were significantly downregulated, while those associated with mitochondrial function^24,25^ were upregulated in cells from atherosclerotic individuals (Fig. 5d). Specifically, gene expression changes in key lipid regulators, including PLA2G4A (cleaves phospholipids to lysophospholipids), ACADM (enhanced fatty acid oxidation) and SREBF2 (cholesterol biosynthesis), suggests phospholipid turnover, increased proinflammatory lipid production, metabolic reprogramming and feedback inhibition of cholesterol biosynthesis due to the excess cholesterol environment (Fig. 5e). Together, omics data suggest that T cells in atherosclerosis undergo coordinated lipid remodelling and metabolic adaptation that fuels immune dysfunction and inflammation. These molecular changes occur in parallel with the observed shifts in membrane order and mitochondrial depolarization. This finding reinforces the conclusion that our affordable method of measuring biophysical properties of many cell types simultaneously is a robust proxy of expensive and time-consuming multi-omics approaches because lipid composition and mitochondrial activity are intimately connected to immune cell biophysics in the context of atherosclerosis.

## Conclusion

We present SBC as a robust, scalable and accessible platform for high-throughput and multiparametric biophysical profiling of immune cells. By integrating environment-sensitive nanosensors with spectral flow cytometry, SBC enables detailed and single-cell characterization of fundamental cellular properties, such as membrane order, mitochondrial depolarization and membrane potential. This approach can be extended to other environment-sensitive nanosensors capable of detecting metabolites and monitoring cellular processes, thereby enabling a more comprehensive characterization of physiological parameters in live systems. In addition, our strategy provides a roadmap for the development of robust, quantitative nanosensors, which currently represent a key limiting factor in expanding our biophysical screening platform beyond two or three sensors.

Applied to PBMCs from individuals with atherosclerosis, SBC successfully identified disease states based solely on biophysical signatures, independent of conventional molecular profiling with omics. Importantly, SBC relies on commercially available instruments and reagents, making it readily adaptable to diverse research and clinical settings. Its ability to simultaneously capture multiple biophysical parameters and integrate them with immunophenotyping renders SBC a powerful tool for biomarker discovery, drug screening and mechanistic exploration. As such, SBC represents opportunities for uncovering biophysical hallmarks of diseases and thus advancing our understanding of how biophysical properties of cells reflect and contribute to pathophysiological processes.

## Methods

### Preparation of BSLBs

1,2-Diarachidonoyl-*sn*-glycero-3-phosphocholine (20:4-20:4 (DAPC)), 1,2-di-(9*Z*-octadecenoyl)-*sn*-glycero-3-phosphocholine (18:1-18:1 Δ9*cis* (DOPC)), 1,2-dipetroselenoyl-*sn*-glycero-3-phosphocholine (18:1-18:1 Δ6*cis*), 1,2-dielaidoyl-*sn*-glycero-3-phosphocholine (18:1-18:1 Δ9*trans*), 1,2-dipalmitoleoysl-*sn*-glycero-3-phosphocholine (16:1-16:1 Δ9*cis*), 1,2-dipalmitelaidoyl-*sn*-glycero-3-phosphocholine (16:1-16:1 Δ9*trans*), 1-stearoyl-2-oleoyl-*sn*-glycero-3-phosphocholine (18:0-18:1), 1-stearoyl-2-linoleoyl-*sn*-glycero-3-phosphocholine (18:0-18:2), 1-palmitoyl-2-oleoyl-*sn*-glycero-3-phosphocholine (16:0-18:1 (POPC)) and cholesterol were from Avanti Polar Lipids. As a first step, 10 µl of each lipid at 25 mg ml^−1^ (in chloroform) was dried under a gentle stream of nitrogen to form a thin lipid film. The dried film was rehydrated in 1 ml of SLB buffer (150 mM NaCl, 10 mM HEPES, pH 7.4), vortexed briefly and sonicated using a tip sonicator (power, 3; duty cycle, 40%) for 10 min to generate small unilamellar vesicles (SUVs). To form BSLBs, 45 µl of the SUV solution was mixed with 5 µl of silica beads (Bangs Labs) in a final volume of 500 µl SLB buffer. The mixture was bath-sonicated at room temperature for 30 min to facilitate bilayer formation on the bead surfaces. The resulting lipid-coated beads were washed three times with SLB buffer to remove unbound lipids. NR12S and Pro12A were synthesized as described previously^9,10^. For flow cytometry analysis, beads were incubated with the membrane order probes Pro12A or NR12S (final concentration, 250 nM) for 1 min in flow cytometry tubes immediately prior to acquisition on a Cytek Aurora spectral flow cytometer (5 lasers, 64 channels). For imaging, beads were plated in an 18-well glass-bottom chamber (ibidi) and excited with a 405-nm laser using a Zeiss LSM 780 confocal microscope equipped with 32-channel spectral detectors.

### Drug treatments on CEM cells

CEM cells (ATCC, CRL-2265) were cultured in RPMI 1640 medium (Sigma-Aldrich) supplemented with 10% FBS (Sigma-Aldrich) under standard conditions. The following compounds were used for drug treatment experiments: CK-666 (5 µg ml^−1^), latrunculin B (1 µg ml^−1^), methyl-β-cyclodextrin (12 µg ml^−1^), sphingomyelinase (1/100 dilution of 25 units), oseltamivir phosphate (40 µg ml^−1^), swainsonine (1 µg ml^−1^), digitonin (5 µg ml^−1^) and valinomycin (25 µM) (all from Sigma-Aldrich), and CCCP (20 µM) (Thermo Fisher). For each condition, 3 × 10^5^ cells were used. If not stated otherwise, all treatments were performed in full medium at 37 °C for 30 min–1 h. Following incubation, cells were washed three times with PBS. Cell viability was assessed using BD Horizon Fixable Viability Stain 660 (FVS660, Live/Dead), applied for 15 min at room temperature, followed by two PBS washes. For membrane order analysis, cells were stained with Pro12A dye (0.6 µl of 100 µM dye in 300 µl PBS for 300,000 cells) for 1 min directly in the flow cytometry tubes immediately before the acquisition. For mitochondrial depolarization measurements, JC-1 dye (Thermo Fisher) was incubated with the cells for 30 min either prior to or during drug treatment. For CCCP treatments, JC-1 (0.5 µl of 250 µM dye in 500 µl medium for 300,000 cells) was added during the last 5, 15 or 30 min of drug exposure, while for Digitonin, JC-1 was added during the final 5 min of treatment. Cells were washed twice prior to Live/Dead staining. For membrane potential measurements with CV1 dye (CytoCybernetics), cells were incubated for 15 min in varying ratios of high-potassium buffer (138 mM KCl, 2 mM NaCl, 2 mM CaCl2, 20 mM HEPES, pH 7.5) and low-potassium buffer (2 mM KCl, 138 mM NaCl, 2 mM CaCl2, 20 mM HEPES, pH 7.5), followed by treatment with valinomycin for 5 min. The Nernst potential for potassium was calculated by the equation:

1 $${E}_{{\rm{K}}}=\frac{-{RT}}{F}\mathrm{ln}\frac{140\,\mathrm{mM}}{[{{\rm{K}}}_{\mathrm{out}}]}$$

where *R* is the gas constant, *T* is the temperature, *F* is Faraday’s constant and [K_out_] is the concentration of potassium in the external solution, assuming an intracellular potassium concentration of 140 mM. Cells were then stained with CV1 (0.5 µl of 50 µM dye in 300 µl PBS for 300,000 cells) for 1 min immediately prior to acquisition. For spectral imaging, cells were plated in an 18-well glass-bottom chamber and imaged using a Zeiss LSM 780, as described previously for Pro12A^11^. Cells with JC-1 were excited with a 488-nm laser, and 19 detectors were set, ranging between 494 and 665 nm with 9-nm intervals. Normalized ratios ((monomer − aggregates)/(monomer + aggregates)) were calculated using wavelengths of 539 nm (monomers) and 592 nm (aggregates). For imaging of CV1, cells were excited with wavelengths of 633 nm and collected between 632 and 694 nm.

### Drug treatments on PBMCs

PBMCs (covered by ethical permit Dnr 2025-01255-01, 2025-02-20) were isolated from 45 ml of buffy coat obtained from healthy donors. The buffy coat was diluted 1:1 with PBS and evenly distributed into two 50-ml Falcon tubes. After thorough mixing with a serological pipette, 30 ml of the diluted sample was carefully layered over 15 ml of Ficoll-Paque (Sigma-Aldrich) in three separate 50-ml tubes. Samples were centrifuged at 400*g* for 25 min without applying the brake. The PBMC layer was collected and transferred into fresh tubes, followed by two PBS washes (300*g*, 5 min, brake on). After the final wash, cells were resuspended in 20 ml PBS, filtered through a 70-µm cell strainer (Thermo Fisher), and centrifuged at 200*g* for 10 min. The supernatant was discarded, and the cell pellet was resuspended in cryopreservation medium (90% FBS, 10% DMSO). Cells were aliquoted (1 ml per vial) and stored at –150 °C until use.

For experiments, frozen PBMCs were thawed by gradual addition of a 10-fold volume of prewarmed RPMI 1640 medium supplemented with 10% FBS, followed by centrifugation at 400*g* for 6 min. Cells were processed in Eppendorf tubes at a final concentration of 6 × 10^5^ cells per condition. For mitochondrial depolarization assays, cells were first incubated with JC-1 dye for 30 min at 37 °C, followed by two PBS washes. Cells were then treated with CCCP for 5 min to induce mitochondrial depolarization and washed twice with PBS. For membrane order analysis, cells were treated with sphingomyelinase (Sigma-Aldrich) for 1 h at 37 °C and subsequently washed twice with PBS. While antibody staining was performed after treatments and JC-1 labelling for mitochondrial depolarization, it was done before Pro12A or/and CV1 labelling.

Following treatments, cells and their untreated controls were used to prepare both single-stained controls and multicolour antibody panels for spectral flow cytometry. Antibody titrations were optimized based on staining index calculations. Each sample was either unstained, stained with a single antibody or probe (for spectral unmixing) or with a full antibody cocktail together with probe(s) in staining buffer (Thermo Fisher). Staining was performed at 4 °C in the dark for 30 min, followed by two PBS washes. Cells were then resuspended in 500 µl PBS, and viability was assessed by adding 1 µl of a Live/Dead dye (1:100 dilution), incubating for 15 min at room temperature in the dark, followed by two final PBS washes. Cells were then resuspended in 300 µl PBS in flow tubes for acquisition.

For Pro12A-only experiments, PBMCs were stained with the following antibodies: anti-human CD3-PerCP-Cy5.5 (clone SK7, BioLegend), CD56-PerCP-eFluor 710 (clone CMSSB, Thermo Fisher), CD4-NovaFluor Yellow 660 (clone SK3, Thermo Fisher), CD197 (CCR7)-PE-Vio770 (clone REA108|TG8, Miltenyi Biotec), CD14-APC (clone M5E2, BioLegend), CD19-Alexa Fluor 647 (clone HIB19, BioLegend), CD11c-APC-R700 (clone 3.9, BD Biosciences), CD45RA-APC-H7 (clone HI100, BD Biosciences), CD8a-APC-Fire 810 (clone SK1, BioLegend) and Fixable Viability Stain 570 (BD Biosciences). For JC-1-only and Pro12A–JC-1 combination experiments, the following antibodies were used: CD3-PerCP-Cy5.5, CD56-PerCP-eFluor 710, CD4-NovaFluor Yellow 660, CD197 (CCR7)-PE-Vio770, CD14-APC, CD11c-APC-R700, CD45RA-APC-H7, CD8a-APC-Fire 810 and Fixable Viability Stain 660 (BD Biosciences). For CV1-only and Pro12A–JC-1–CV1 combination experiments, the antibody panel included: CD56-PerCP-eFluor 710, CD19-Alexa Fluor 647, CD45RA-APC-H7 and CD8a-APC-Fire 810.

### PBMCs from individuals with atherosclerosis

This study was approved by the Lokman Hekim University Scientific Research Ethics Committee (approval number 2024/46; approval date 30 January 2024) and by Ethics Review Authority Stockholm (Dnr 2025-02560-01, 2025-04-11). Written informed consent was received from donors prior to the study. All procedures involving human participants adhered to the ethical principles outlined in the 1964 Helsinki Declaration and its subsequent amendments or equivalent ethical standards. PBMC samples were obtained from patients with angiographically confirmed coronary artery disease, but without prior myocardial infarction, as part of the inclusion criteria. Sex- and age-matched healthy subjects had no known cardiovascular disease or drug treatment for it, had never smoked, had no history of diabetes and had no family history of cancer. Whole blood from 38 patients and 26 healthy controls was drawn into EDTA tubes (Becton Dickinson) and PBMCs were isolated immediately. No statistical methods were used to predetermine sample sizes. The samples were not randomized, and data collection and analysis were not performed blind to the conditions of the experiments to be able to measure the same number of healthy and patient material on each day. No donors were excluded. Peripheral blood was diluted 1:1 with 1× DPBS and carefully layered over an equal volume of Ficoll-Paque solution in a 50-ml conical tube, tilted at ∼45°, to maintain phase separation. The tubes were centrifuged at 400*g* for 30 min at room temperature with minimal acceleration and no brake to preserve interface integrity. The mononuclear cell layer (buffy coat) was collected, washed sequentially with 1× DPBS and IMDM medium (Thermo Fisher), and centrifuged at 300*g* for 10 min between washes. After the final wash, cells were cryopreserved at 1 × 10^6^ cells per cryovial in freezing medium (90% FBS + 10% DMSO). The cryovials were cooled at a rate of 1 °C min^−1^ in a Mr Frosty and stored overnight at −86 °C. All vials were subsequently transferred to liquid nitrogen until analysis. The handling of the PBMCs and the analysis pipeline for membrane order and mitochondrial depolarization with Pro12A and JC-1 is described above.

### SBC measurements and analysis

Spectral flow cytometry data obtained with a Cytek Aurora (Supplementary Data 1) were analysed using FCS Express 7 (DeNovo Software), and downstream computational analyses were conducted using Python-based pipelines. Spectral unmixing was performed within FCS Express using unstained, autofluorescence for lymphocytes and monocytes, single-stained controls for each fluorochrome, and probes to accurately resolve signal overlap across channels. Spectral signatures were carefully curated and reviewed to minimize spillovers and maintain optimal separation between fluorochromes.

To identify the optimal fluorescence channel pair for separating experimental conditions, we computed ratiometric values for all dual-channel combinations. Each pair was evaluated using the Kolmogorov–Smirnov statistic and the absolute difference in median ratios. An overall score combining both metrics was used to rank channel pairs, with adjustments for high intragroup variance. The top-ranking pair was selected as the most discriminative together with excitation/emission properties of the probes. For BSLBs stained with Pro12A or NR12S, V1–V9 (428–598 nm) and YG2–YG6 (598–697 nm) channels were used, respectively. For cellular data, Pro12A emission was captured in V1–V7 (428–542 nm), JC-1 in B3–B5 (542–598 nm) and CV1 in B7–R5 (661–738 nm). When Pro12A and JC-1 were coincubated, V1–V5 (428–508 nm) were selected for membrane order quantification. When all probes were combined (Pro12A, JC-1, CV1), V1–V5 were used for Pro12A and B9–R5 (697–738 nm) for CV1. ROUT 1% outlier was applied to eliminate instrument-related erroneous or disproportionately large values. Gating strategies for all conditions, including singlet discrimination, avoiding internalized probes, viability gating, and major immune lineage identification, are detailed (Supplementary Figs. 10–15).

To explore high-dimensional spectral features and visualize population-level differences, UMAP was used for dimensionality reduction. Raw fluorescence intensity values were first normalized on a per-cell basis by dividing each channel’s value by the maximum intensity across all channels for that cell. This per-cell normalization preserved the relative spectral composition while mitigating absolute intensity variability. The intensities were then normalized using StandardScaler (scikit-learn), transforming each feature to have zero mean and unit variance. UMAP was implemented using the umap-learn package with parameters tuned to balance global and local structure preservation (for example, n_neighbors, min_dist). These parameters were chosen to maintain separation between biologically meaningful subpopulations while preserving finer structure in the data. For classification and predictive modelling tasks, we utilized Extreme Gradient Boosting (XGBoost) via the xgboost Python library. Prior to modelling, input data were scaled using StandardScaler and labels were numerically encoded using LabelEncoder (scikit-learn). Bayesian hyperparameter optimization was conducted using the skopt package (Bayesian optimization with Gaussian Processes), with search space definitions spanning key XGBoost hyperparameters (for example, learning_rate, n_estimators, max_depth, subsample, colsample_bytree, reg_alpha, reg_lambda, gamma, min_child_weight). Model performance was evaluated using stratified *k*-fold cross-validation (typically 10-fold; 5-fold for small sample sets), with classification accuracy and confusion matrices.

### scRNA-seq of biophysically selected PBMCs

PBMC samples (four atherosclerosis and four sex- and age-matched healthy controls (A. Healthy)) were processed for scRNA-seq using the 10x Genomics Chromium Single Cell 3′ v.4 platform (Supplementary Data 2), following the manufacturer’s protocol. Briefly, after thawing and washing, cells were resuspended in PBS containing 0.04% BSA, counted and assessed for viability. Cell suspensions were loaded into the Chromium Controller to generate gel bead-in-emulsions, followed by reverse transcription, cDNA amplification and library construction using the Single Cell 3′ v.4 Reagent Kit. Final libraries were quality-checked using an Agilent Bioanalyzer and quantified by quantitative polymerase chain reaction. Sequencing was performed on an Illumina NovaSeq X Plus system (NovaSeq X Series Control Software v.1.2.2.48004) using a 151 nucleotide (nt) (read 1) – 10 nt (index 1) – 10 nt (index 2) – 151 nt (read 2) configuration on a ‘25B’ mode flow cell.

Sequencing data were processed with Cell Ranger v.9.0.1 using the GRCh38-2024-A reference genome to generate filtered gene-barcode matrices. Downstream analysis was conducted in R v.4.4.3 using the Seurat v.5.2.1 pipeline^26^. Briefly, standard quality control excluded cells with >20% mitochondrial content or <5% ribosomal gene expression. Genes such as *MALAT1*, mitochondrial (*MT-*), ribosomal (*RPS*, *RPL*), and haemoglobin (*HB*) genes were also excluded. Sample-specific expected doublet rates (ranging from 1.2% to 7.2%) were applied using DoubletFinder to identify and exclude potential doublets. The final dataset was normalized, scaled and subjected to PCA and UMAP for dimensionality reduction, followed by graph-based clustering using the Louvain algorithm and GSEA-informed cell-type prediction. To focus on immune dynamics, we specifically selected T cells and redefined identities based on disease condition (Atherosclerosis versus A. Healthy). Differential gene expression analysis was performed using the Wilcoxon rank-sum test with conservative filtering (logfc.threshold = 0.25, min.pct = 0.15), identifying genes significantly up- or downregulated in the disease context. These gene sets were analysed for pathway enrichment using the GO Biological Process 2023 database via enrichr. Enrichment results were filtered for pathways containing keywords relevant to lipids and mitochondria and visualized with −log_10_(*P* value) bar plots to highlight condition-specific changes in T-cell biology.

### Mass spectrometry of biophysically selected PBMCs

We performed direct infusion shotgun lipidomics analysis on selected atherosclerosis (*n* = 4) and sex- and age-matched healthy control (*n* = 4) samples, using the same donor material as for the scRNA-seq analysis. Prior to lipidomics, primary human T cells were isolated from PBMCs via negative magnetic selection using the MojoSort Human CD3 T Cell Isolation Kit (BioLegend), following the manufacturer’s protocol. The resulting cell populations exhibited >90% purity. Cells were counted using a BioRad TC20 cell counter and assessed for viability with trypan blue staining. Subsequently, cells were washed twice with PBS, followed by a final wash with 140 mM ammonium formate. Cell pellets were then snap-frozen and stored −80 °C until the analysis. Prior to sampling, the cells were rapidly thawed and lysed using methanol:H_2_O 9:1 v/v (0.1% formic acid), targeting a final cell concentration of 1000 cells µl^−1^ in each sample. The cell lysates were centrifuged for 1 min at 8,000 r.p.m., and the supernatant was directly analysed by immersing the direct infusion probe^27^ in the solvent. The electrospray voltage was directly applied on the steel capillary and was set to 2,150 V. Mass spectrometry was performed in positive-ion mode on an Orbitrap IQ-X (Thermo Fisher Scientific) instrument where the AGC target was 40% and scans were acquired at 240,000 resolution (at *m/z* 200) in the mass range of *m/z* 250–1,200. For each measurement, a 1-min-long direct infusion was performed, corresponding to 120 individual mass spectra on average. The acquired RAW data files were converted to .mzML using MSConvertGUI (ProteoWizard, v.3.0.22285). Analytes were identified based on accurate mass match (3 ppm tolerance) to our in-house library using custom-made MATLAB scripts and the total-ion-current-normalized intensity of molecular features were extracted from each spectrum and averaged through all scan events. Next, the identities of detected molecules were confirmed using tandem mass spectrometry measurements on pooled samples. Specifically, we pooled equal volumes of all four control and all four atherosclerotic samples and then performed higher-energy collisional dissociation fragmentation using normalized collisional energies of 30, 40 and 50. All tandem mass spectrometry spectra were manually curated for structural annotation, and in the case of multiple adduct forms of the same molecular feature, only the highest intensity one was used for further data analysis. Sparse partial least squares (PLS) discriminant analysis was performed to identify features distinguishing between atherosclerosis and healthy T-cell lipidomes. Lipid intensity values were standardized first and the top 30 discriminative features were selected using univariate analysis of variance (ANOVA) (SelectKBest, f_classif). PLS discriminant analysis was conducted using two components, and the explained variance per component was calculated. Analyses were conducted using Python’s scikit-learn and matplotlib libraries.

### Reporting summary

Further information on research design is available in the Nature Portfolio Reporting Summary linked to this article.

## Online content

Any methods, additional references, Nature Portfolio reporting summaries, source data, extended data, supplementary information, acknowledgements, peer review information; details of author contributions and competing interests; and statements of data and code availability are available at https://doi.org/10.1038/s41565-026-02236-8.

## Supplementary information

Supplementary Figs. 1–19.

Reporting Summary

Peer Review File

Supplementary Data 1 Flow cytometry channels.

Supplementary Data 2 Quality control for scRNA-seq.

## Source data

Source Data Fig. 1 Excel sheets for data.

Source Data Fig. 2 Excel sheets for data.

Source Data Fig. 3 Excel sheets for data.

Source Data Fig. 4 Excel sheets for data.

Source Data Fig. 5 Excel sheets for data.

## Data Availability

Data presented in the figures are available from figshare via https://doi.org/10.17044/scilifelab.30406327 (ref. ^28^). Single-cell transcriptomics data are available in the GEO database with accession code GSE335789. Source data are provided with this paper.
