# Supplementary material for: Spectral biophysical cytometry with nanosensors reveals remodelling of immune cells in atherosclerosis
Source: Nat Nanotechnol. 2026 Aug 6;21(9):1308–15. doi: 10.1038/s41565-026-02236-8 (PMC13569417; doi:10.1038/s41565-026-02236-8)
Supplement: Supplementary file 1 [file 41565_2026_2236_MOESM1_ESM.pdf]

nature nanotechnology

Supplementary information https://doi.org/10.1038/s41565-026-02236-8

Spectral biophysical cytometry with nanosensors reveals remodelling of immune cells in atherosclerosis

In the format provided by the authors and unedited

Table of Contents

Supplementary fig. 1: Characterization of bead-supported lipid bilayers (BSLBs) for probe labeling.
Supplementary fig. 2: Probe sensitivity to bead-supported lipid bilayers (BSLB) composition.
Supplementary fig. 3: Cell characterization with Pro12A and LiveDead staining.
Supplementary fig. 4: Membrane order measurements of CEM cells treated with actin-targeting drugs.
Supplementary fig. 5: Membrane order measurements of CEM cells treated with membrane-targeting drugs.
Supplementary fig. 6: Membrane order measurements of CEM cells treated with glycocalyx-targeting drugs.
Supplementary fig. 7: Mitochondrial depolarization measurements of CEM cells treated with mitochondria-targeting drugs.
Supplementary fig. 8: Membrane potential measurements of CEM cells under varying extracellular KCl concentrations.
Supplementary fig. 9: Multi-parametric biophysical phenotyping of CEM cells.
Supplementary fig. 10: Membrane order analysis of PBMCs treated with sphingomyelinase.
Supplementary fig. 11: Mitochondrial depolarization analysis of PBMCs treated with CCCP.
Supplementary fig. 12: Membrane potential analysis of PBMCs incubated with KCl buffer.
Supplementary fig. 13: Membrane order and mitochondrial depolarization analysis of PBMCs treated with CCCP v1.
Supplementary fig. 14: Membrane order and mitochondrial depolarization analysis of PBMCs treated with CCCP v2.
Supplementary fig. 15: Membrane order, mitochondrial depolarization, and membrane potential analysis of PBMCs treated with sphingomyelinase v1.
Supplementary fig. 16: Membrane order, mitochondrial depolarization, and membrane potential analysis of PBMCs treated with sphingomyelinase v2.
Supplementary fig. 17: Baseline biophysical heterogeneity across immune cell types in healthy donors.
Supplementary fig. 18: Multi-parametric biophysical profiling of atherosclerosis by immune cell type.
Supplementary fig. 19: Feature contributions to PLS components in atherosclerosis PBMC analysis.

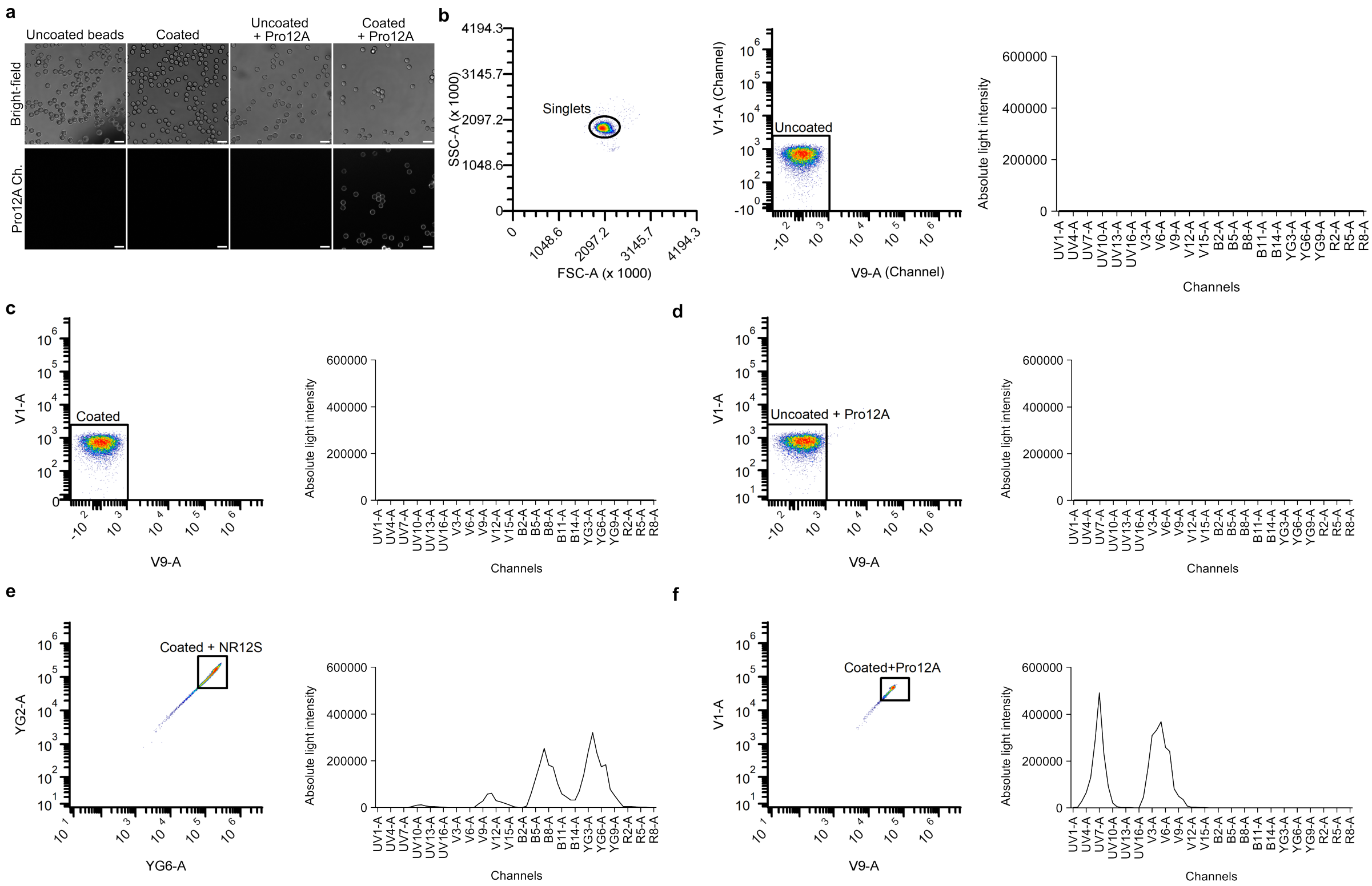

Supplementary fig. 1: Characterization of bead-supported lipid bilayers (BSLBs) for probe labeling.

a, Representative microscopy images of silica beads at different preparation steps: uncoated beads (bare beads), beads after coating with lipid bilayers, and beads after fluorescent labeling, confirming successful lipid coating and probe incorporation. **b**, Spectral flow cytometry analysis of BSLBs showing forward scatter (FSC) and side scatter (SSC) profiles to distinguish bead populations. The x-axis and y-axis represent detector channels (middle graph), with the optimized detection window for the membrane order probe, Pro12A. Data are shown for uncoated beads as negative control. **c–f**, Optimized fluorescence detection channels for different bead conditions: **(c)** coated beads without dye, **(d)** uncoated beads labeled with Pro12A, **(e)** coated beads labeled with NR12S, and **(f)** coated beads labeled with Pro12A. These comparisons demonstrate that membrane-sensitive probes selectively incorporate into lipid bilayers and that their spectral signatures can be reliably resolved using spectral flow cytometry. Each sample includes measurements from around ten thousand beads.

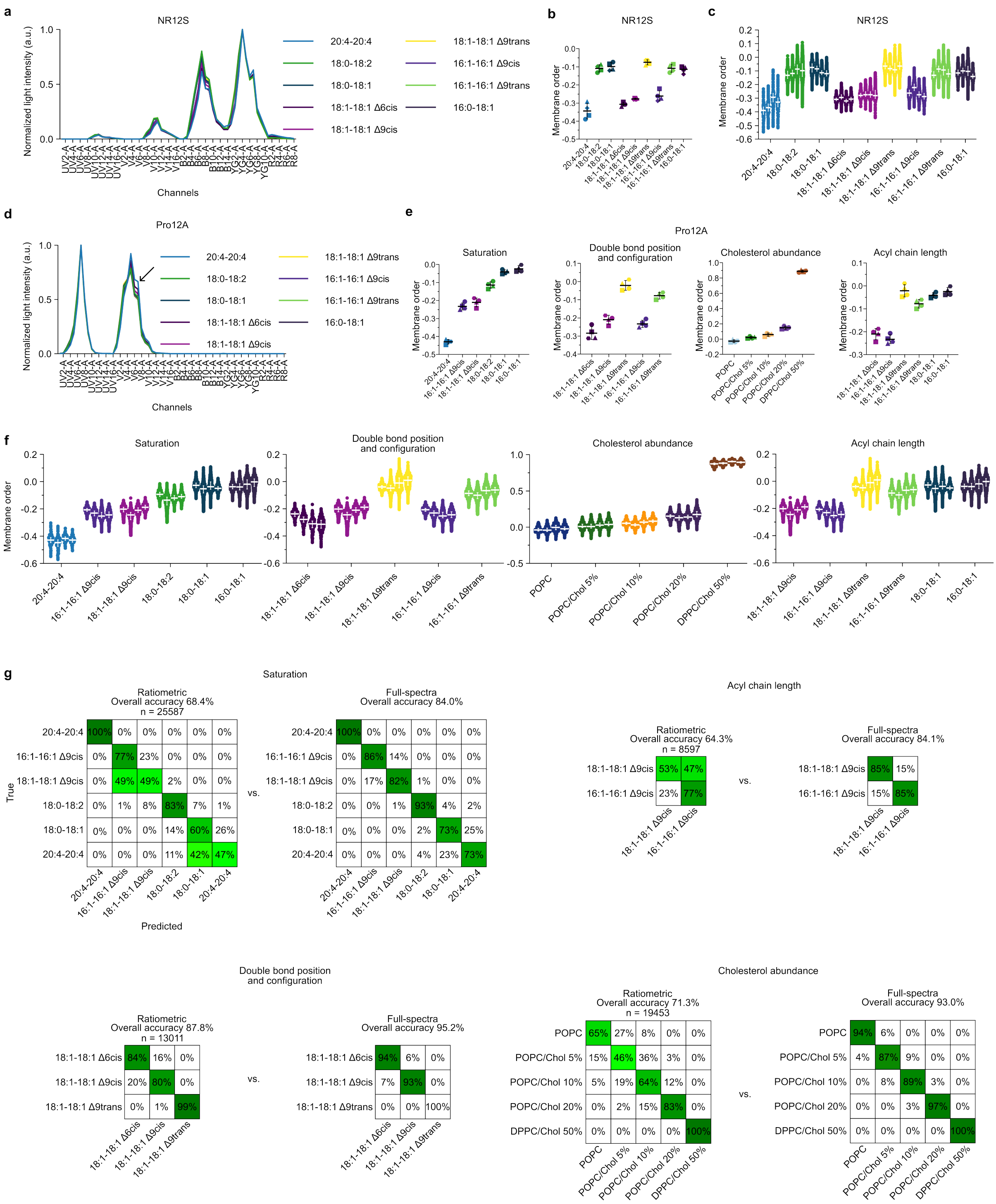

Supplementary fig. 2: Probe sensitivity to bead-supported lipid bilayers (BSLB) composition.

a–c, Spectral flow cytometry of NR12S-labeled BSLBs across a panel of lipid compositions. **(a)** Normalized emission spectra highlight probe sensitivity to differences in bilayer composition. **(b)** Median membrane order values derived from ratiometric analysis across lipid categories. **(c)** Single-bead membrane order distributions, with white lines showing median and interquartile range. **d–f**, Spectral flow cytometry of Pro12A-labeled BSLBs. **(d)** Normalized emission spectra across all lipid compositions. Arrow shows increased relative emission at longer wavelengths corresponds to a red shift associated with lower membrane order. **(e)** Median membrane order values grouped by lipid category. **(f)** Single-bead membrane order

distributions, with white lines indicating median and interquartile range. Black lines in panels b and e indicate mean ± SD across replicates. **g**, Machine-learning–based classification of lipid categories using an XGBoost model, comparing the performance of ratiometric versus full-spectrum analysis. Full-spectrum analysis provides improved separation of BSLBs differing in lipid saturation, double-bond position, cholesterol content, and acyl chain length. Data are representative of four independent experiments, and colors throughout indicate replicate medians. Black lines in graphs b and e indicate the mean ± SD. Each sample includes measurements from around ten thousand beads.

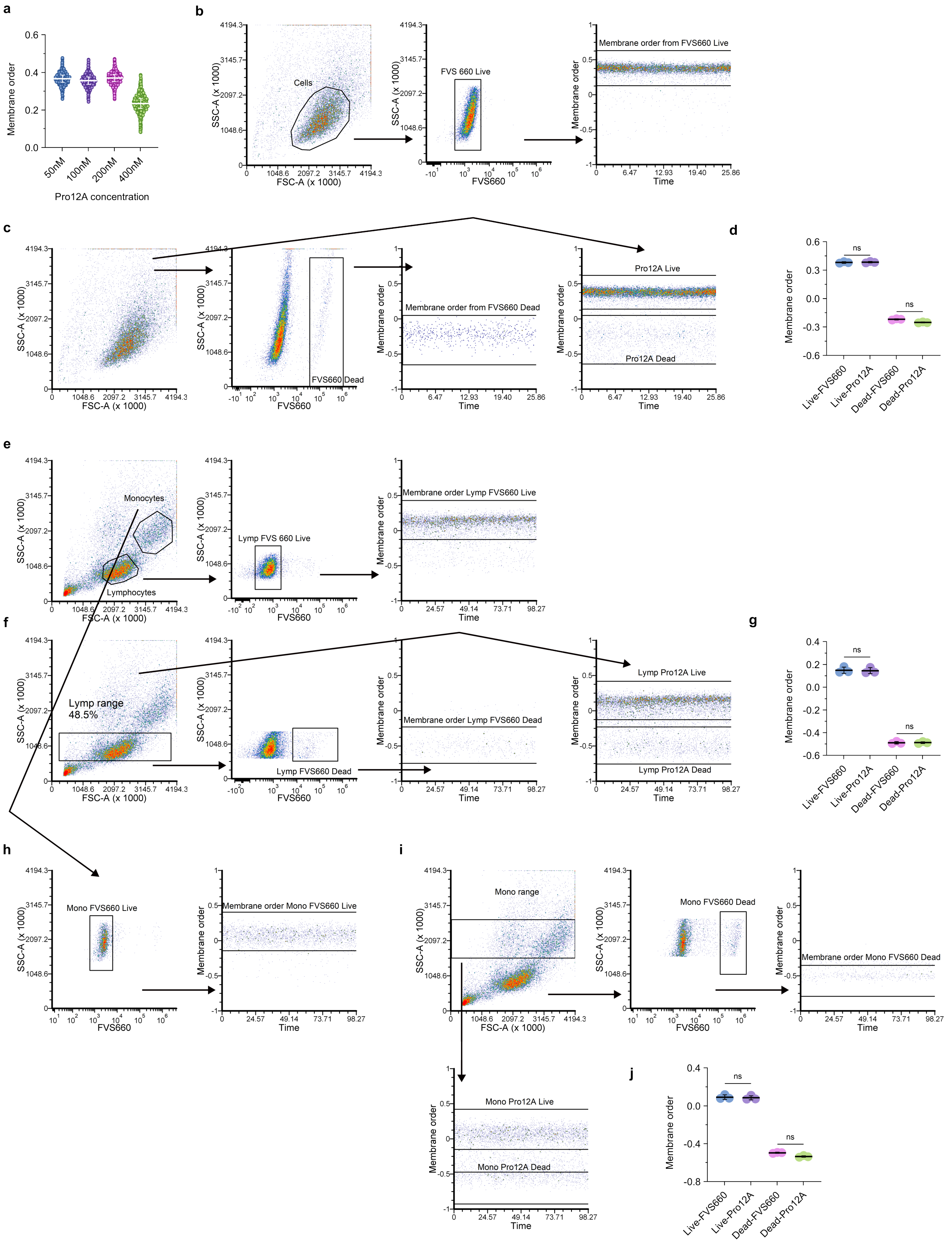

Supplementary fig. 3: Cell characterization with Pro12A and LiveDead staining. **a**, Titration of Pro12A in CEM cells to determine the optimal probe concentration for labeling, shown as single-cell distributions of membrane order. White lines indicate the median and interquartile range. **b–c**, Membrane order measurements of cells gated by viability using the LiveDead (FVS660) dye. **(b)** Live-gated cells, **(c)** Dead-gated cells. **d**, Direct comparison of live and dead cell populations gated either by LiveDead staining or by Pro12A labeling, showing that Pro12A can effectively separate viable

and non-viable populations. **e–f**, Membrane order measurements lymphocytes of blood cells gated by viability using the LiveDead dye. **(e)** Live-gated cells, **(f)** Dead-gated cells. **g**, Direct comparison of live and dead cell populations gated either by LiveDead staining or by Pro12A labeling, showing that Pro12A can effectively separate viable and non-viable populations in lymphocytes. **h–i**, Membrane order measurements monocytes of blood cells gated by viability using the LiveDead dye. **(h)** Live-gated cells, **(i)** Dead-gated cells. **j**, Direct comparison of live and dead cell populations gated either by LiveDead staining or by Pro12A labeling, showing that Pro12A can effectively separate viable and non-viable populations in monocytes. Black lines indicate mean ± SD. Data are representative of three independent experiments. Statistical significance was evaluated using a one-way nonparametric Kruskal–Wallis test: ns, not significant. Each sample includes measurements from at least five to ten thousand cells.

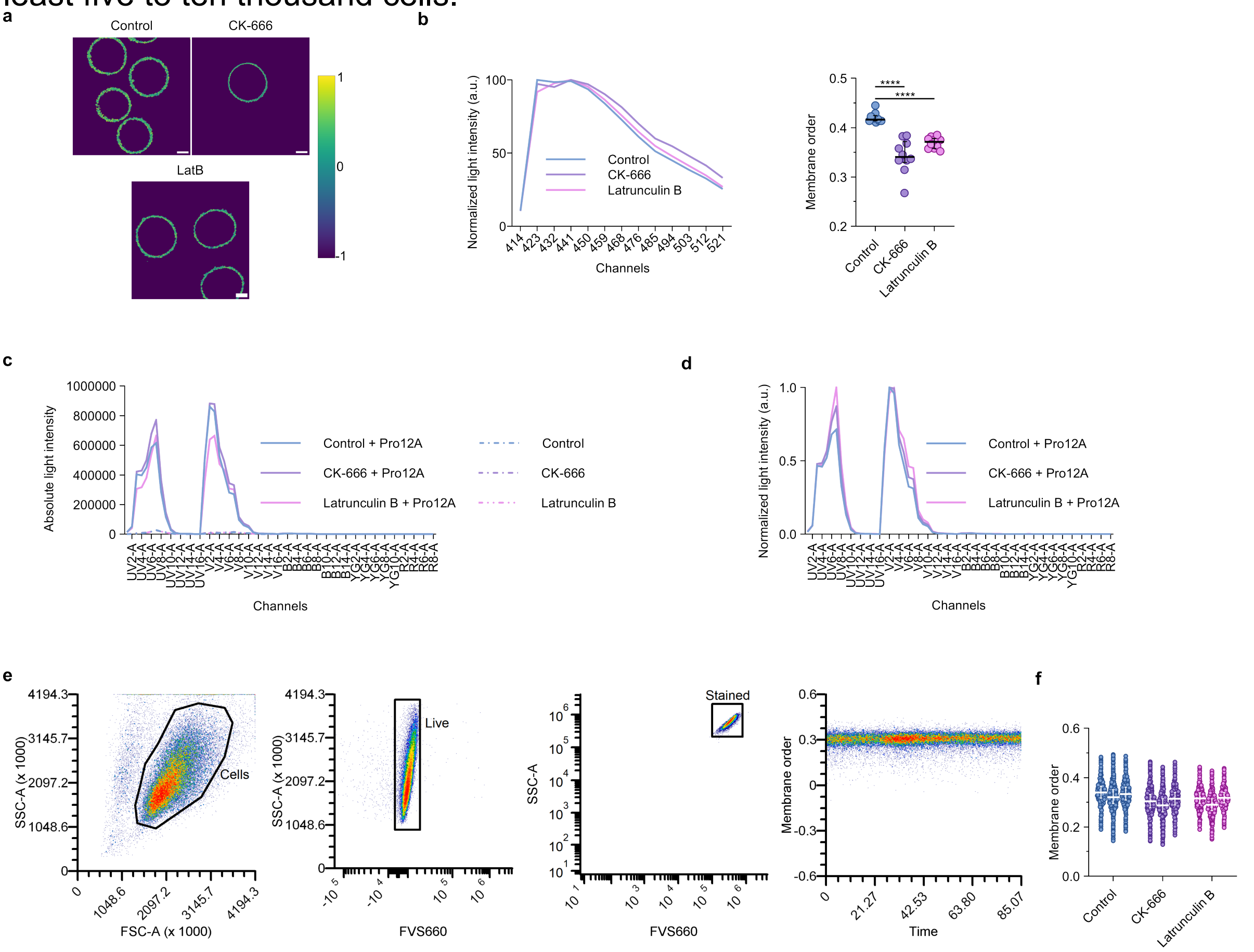

Supplementary fig. 4: Membrane order measurements of CEM cells treated with actin-targeting drugs.

a, Spectral imaging of CEM cells treated with CK-666 (Arp2/3 inhibitor) or Latrunculin B (actin polymerization inhibitor), showing changes in membrane order index (values range from −1 to 1, where higher values (brighter) indicate more ordered membranes and lower values (darker) indicate more disordered membranes). Scale bar, 5 µm. **b**, Corresponding normalized emission spectra and calculated membrane order values for treated versus untreated cells. Black lines indicate the median and interquartile range. **c**, Spectral flow cytometry of Pro12A-stained and unstained cells, comparing absolute fluorescence intensities between treated and untreated groups. **d**, Normalized spectra across all channels for Pro12A-labeled cells highlighting spectral shifts induced by drug treatment. **e**, Representative gating strategy for single-cell

analysis of treated versus untreated populations. **f**, Single-cell membrane order values across biological replicates. White lines indicate the median and interquartile range. Data are representative of three independent experiments. Statistical analysis was tested by two-tailed nonparametric t test with Mann–Whitney; **** p<0.0001. For flow cytometry measurements, each sample includes measurements from at least five to ten thousand cells.

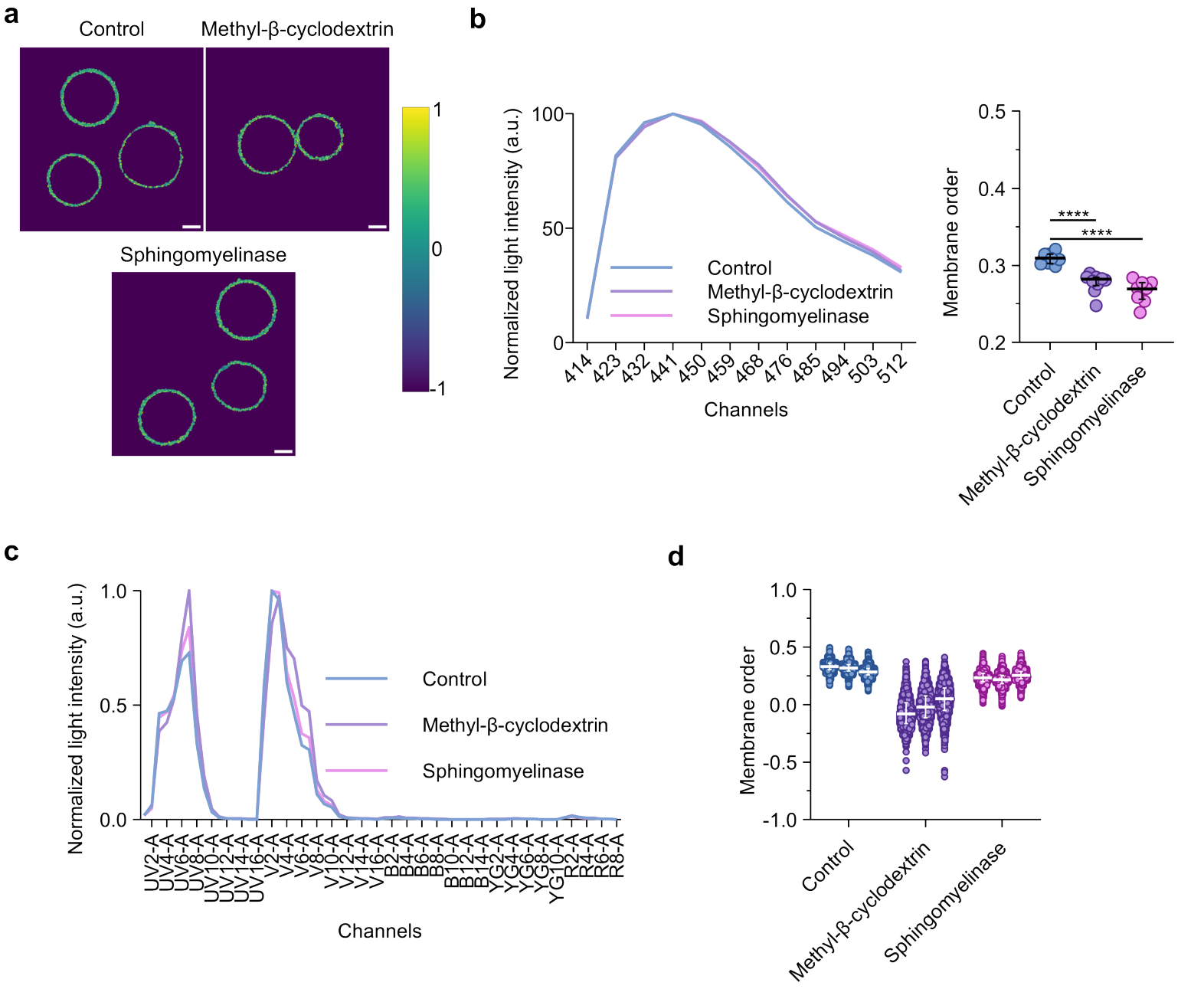

Supplementary fig. 5: Membrane order measurements of CEM cells treated with membrane-targeting drugs.

a, Spectral imaging of CEM cells treated with methyl-β-cyclodextrin (cholesterol depletion) or sphingomyelinase (hydrolyzes sphingomyelin), showing alterations in membrane order index (−1 to 1, with higher values indicating more ordered and lower values indicating more disordered membranes). Scale bar, 5 μm. **b**, Corresponding normalized emission spectra and calculated membrane order for treated versus untreated cells. Black lines represent the median and interquartile range. **c**, Spectral flow cytometry analysis of Pro12A-stained cells, showing normalized fluorescence spectra across all detection channels. **d**, Single-cell membrane order distributions across replicates, highlighting treatment-induced shifts. White lines indicate the median and interquartile range. Data are representative of three independent experiments. Statistical analysis was tested by two-tailed nonparametric t test with Mann–Whitney; **** p<0.0001. For flow cytometry measurements, each sample includes measurements from at least five to ten thousand cells.

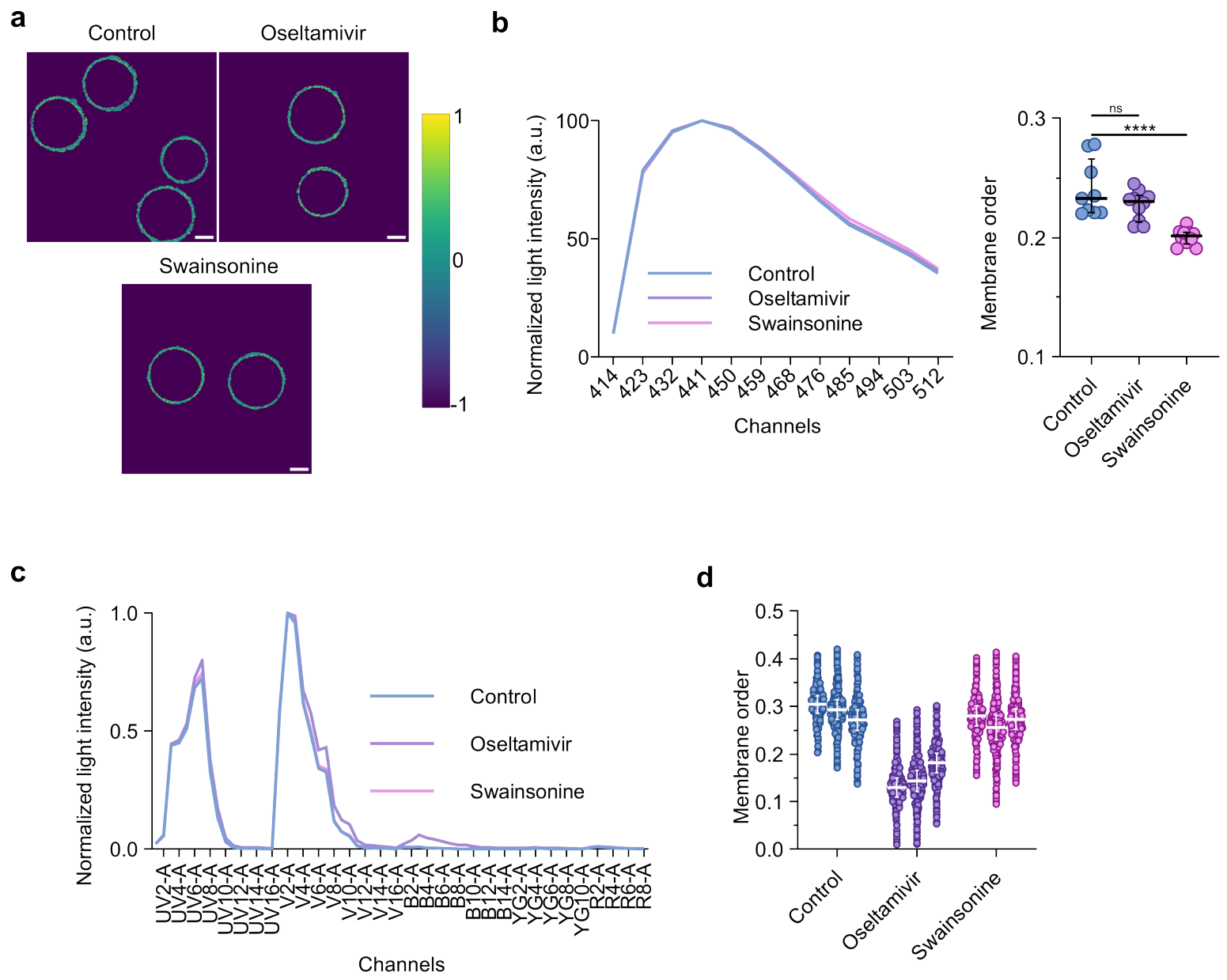

Supplementary fig. 6: Membrane order measurements of CEM cells treated with glycocalyx-targeting drugs.

a, Spectral imaging of CEM cells treated with Oseltamivir (neuraminidase inhibitor, alters sialic acid residues on the glycocalyx) or Swainsonine (α-mannosidase II inhibitor, disrupts N-glycan maturation), showing changes in membrane order index (−1 to 1, with higher values reflecting more ordered and lower values reflecting more disordered membranes). Scale bar, 5 μm. **b**, Corresponding normalized emission spectra and calculated membrane order values for treated versus untreated cells. Black lines indicate the median and interquartile range. **c**, Spectral flow cytometry of Pro12A-labeled cells, showing normalized fluorescence spectra across all detection channels. **d**, Single-cell membrane order distributions across biological replicates, highlighting glycocalyx-dependent shifts. White lines indicate the median and interquartile range. Data are representative of three independent experiments. Statistical analysis was tested by two-tailed nonparametric t test with Mann–Whitney; **** $p<0.0001$; ns, not significant. For flow cytometry measurements, each sample includes measurements from at least five to ten thousand cells.

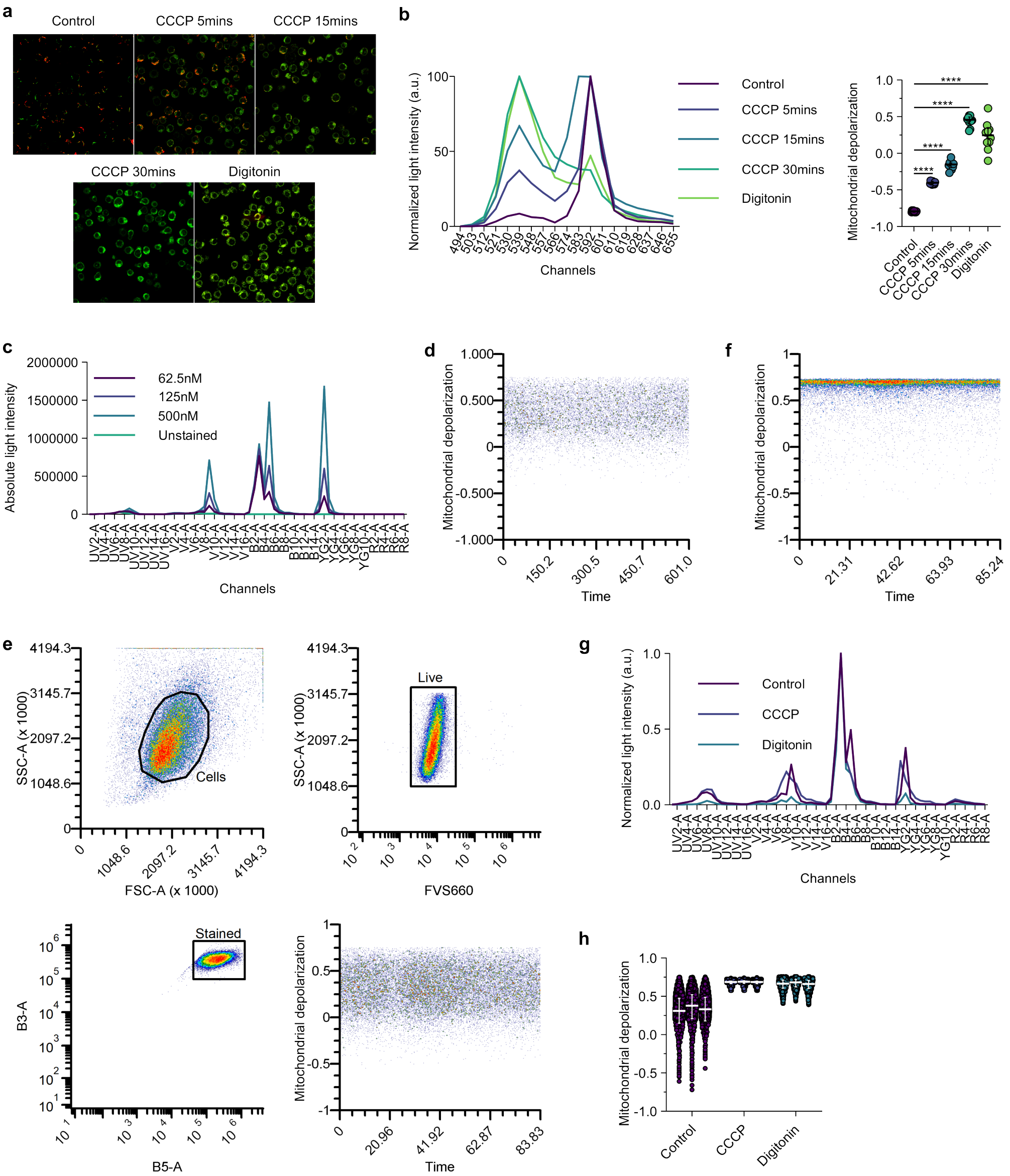

Supplementary fig. 7: Mitochondrial depolarization measurements of CEM cells treated with mitochondria-targeting drugs.

a, Spectral imaging of CEM cells treated with CCCP (protonophore uncoupler) and Digitonin (permeabilizes plasma membrane and indirectly affects mitochondria), showing altered JC-1 fluorescence as a proxy for mitochondrial membrane potential. Scale bar, 5 μm. **b**, Corresponding normalized emission spectra and calculated mitochondrial depolarization index (higher values indicate greater depolarization thus lower mitochondrial potential). Black lines represent the median and interquartile range. **c**, Spectral flow cytometry of cells labeled with varying JC-1 concentrations, shown as absolute intensities across all channels to determine optimal probe concentration. **d**, Temporal stability analysis of JC-1 signal over 10 minutes, confirming stable depolarization readout. **e**, Representative gating strategy applied on untreated samples to identify viable singlet populations. **f**, Quantification of mitochondrial depolarization changes in CCCP-treated cells. **g**, Spectral flow cytometry showing normalized fluorescence spectra across all detection channels for JC-1-labeled cells. **h**, Single-cell mitochondrial depolarization distributions across biological replicates. White lines indicate the median and interquartile range. Data are representative of three independent experiments. Statistical analysis was tested by

two-tailed nonparametric t test with Mann–Whitney; **** $p<0.0001$. For flow cytometry measurements, each sample includes measurements from at least five to ten thousand cells.

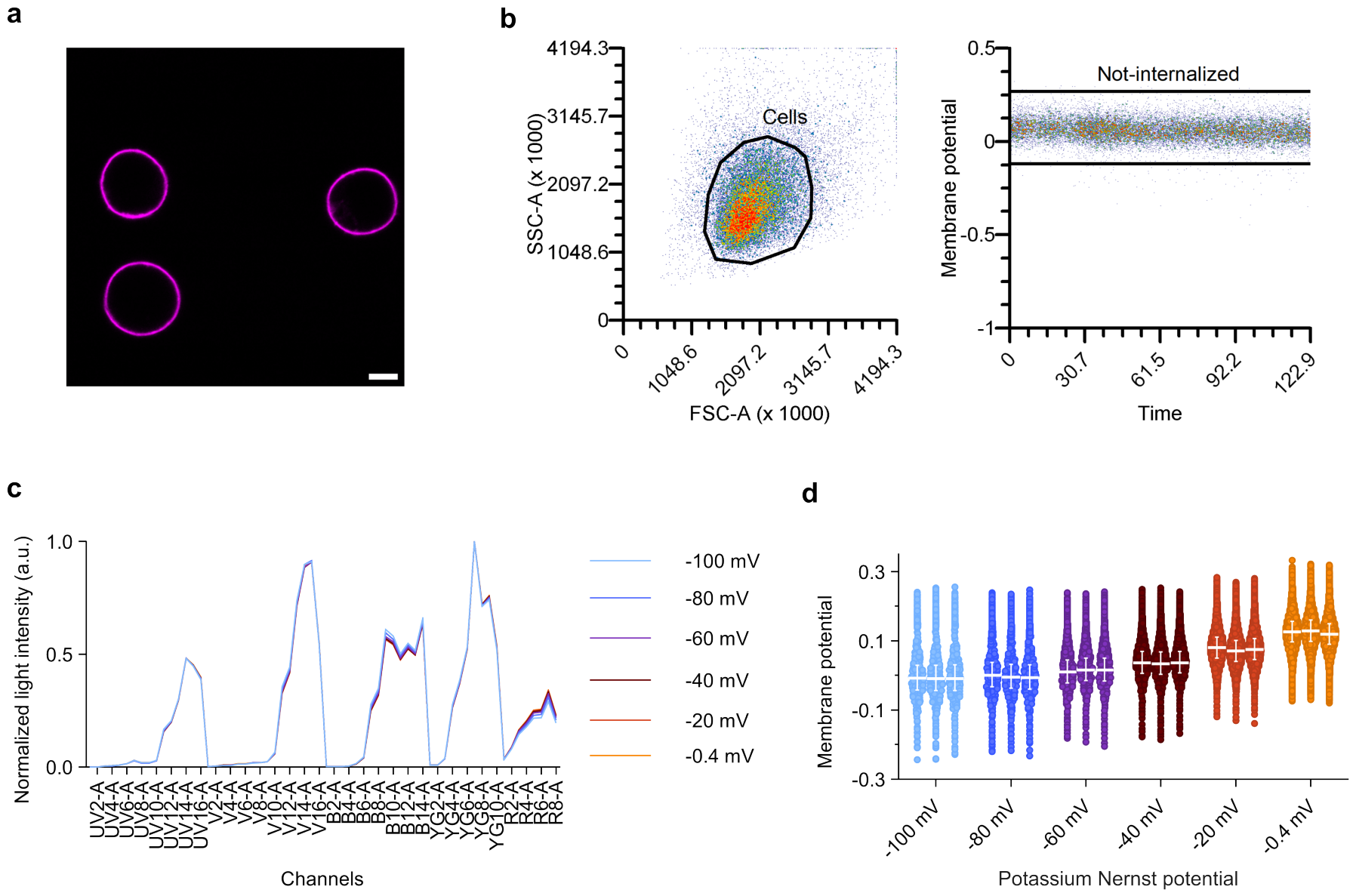

Supplementary fig. 8: Membrane potential measurements of CEM cells under varying extracellular KCl concentrations.

a) Representative image of CEM cells labeled with membrane potential dye, CV1. Scale bar, 5 µm. **b)** Representative spectral flow cytometry gating strategy used to identify viable singlet cells for analysis of membrane potential. **c)** Normalized fluorescence spectra across all detection channels for CV1-labeled cells, illustrating probe response under varying extracellular buffer conditions. **d)** Single-cell membrane potential distributions across biological replicates. White lines indicate the median and interquartile range. Data are representative of three independent experiments. Each sample includes measurements from at least five to ten thousand cells.

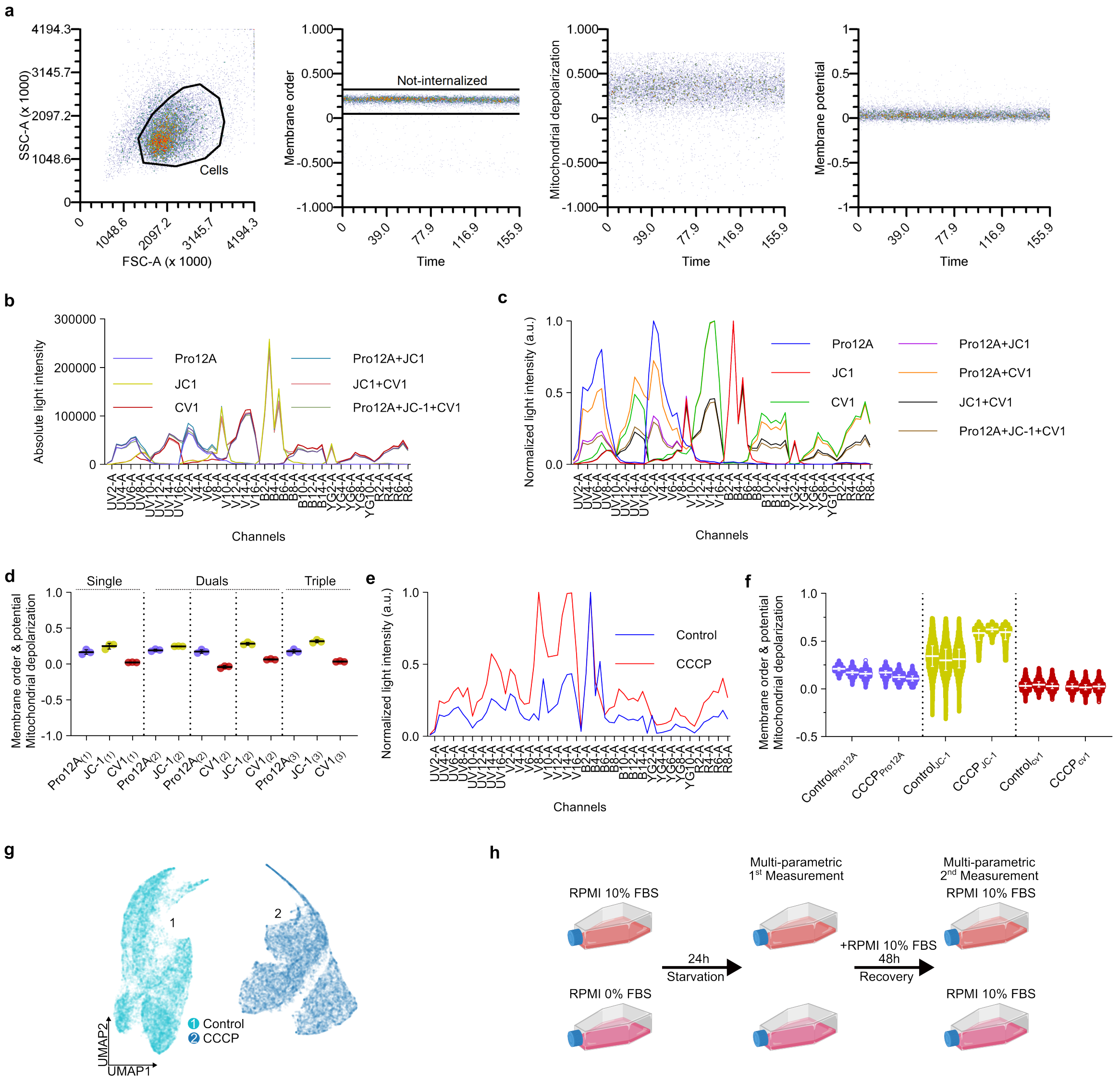

Supplementary fig. 9: Multi-parametric biophysical phenotyping of CEM cells. **a**, Representative spectral flow cytometry gating strategy used to identify viable singlet cells for analysis across multiple probes. **b**, Absolute fluorescence intensities for different probe combinations: Pro12A (membrane order), JC-1 (mitochondrial depolarization), and CV1 (membrane potential), demonstrating probe incorporation and compatibility. **c**, Normalized fluorescence spectra across all detection channels for the same probe combinations. **d**, Comparison of calculated biophysical properties (membrane order, mitochondrial depolarization, and membrane potential) derived from single, dual, and triple probe measurements. Colors indicate replicate medians. Black lines indicate mean ± SD. **e**, Normalized spectral profiles of CCCP-treated cells labeled with all three probes, highlighting coordinated changes across parameters. **f**, Calculated biophysical parameters for each replicate. White lines indicate median and interquartile range. **g**, Full-spectrum projection of multiparametric data, illustrating separation of cell states in high-dimensional space. **h**, Schematic of a starvation-recovery experiment to assess dynamic changes in membrane order, mitochondrial depolarization, and membrane potential using multiparametric profiling. Data are representative of three independent experiments. Each sample includes measurements from at least five to ten thousand cells.

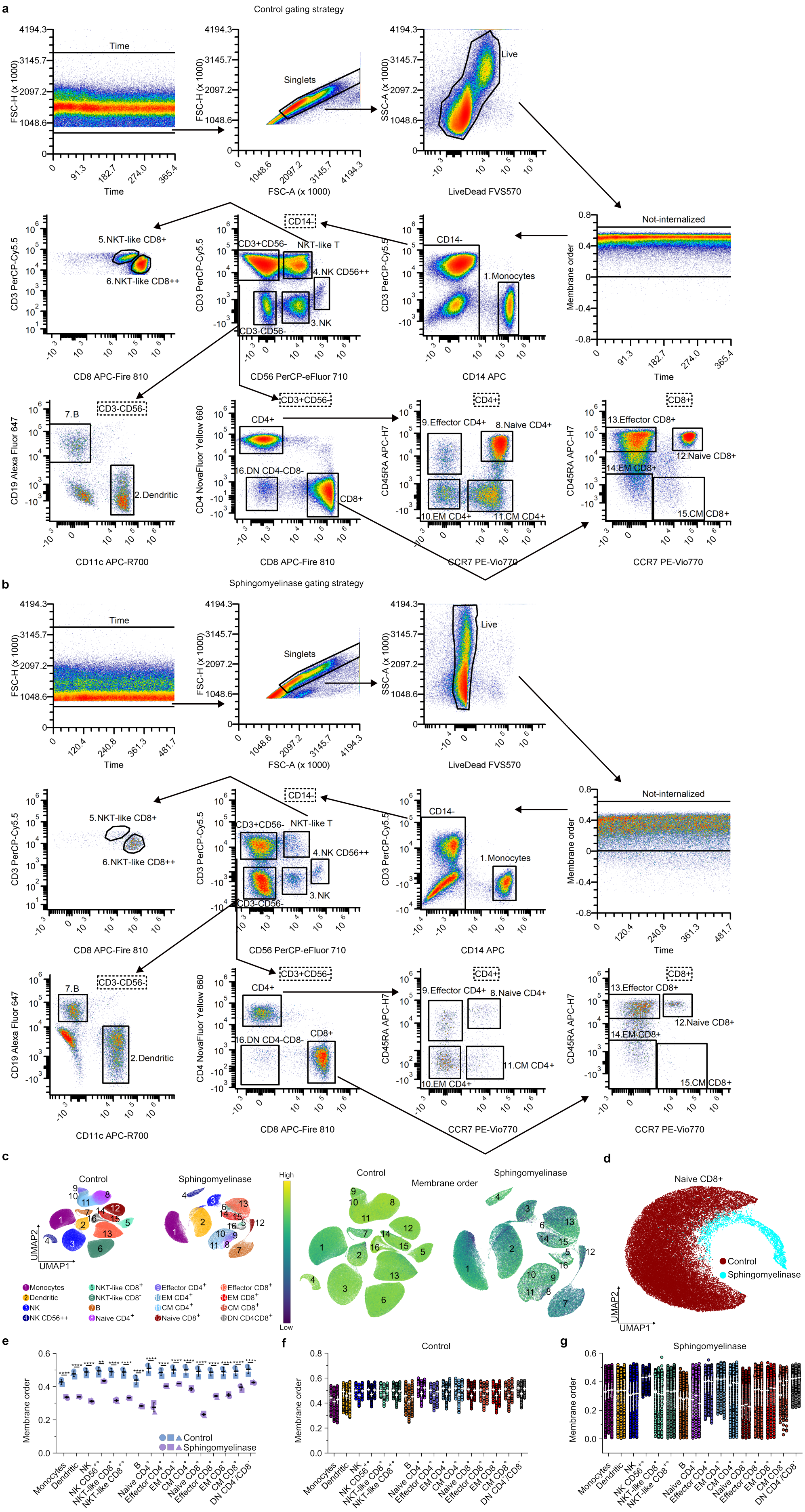
a
Control gating strategy
Time
Singlets
Live
FSC-H (x 1000)
FSC-A (x 1000)
SSC-A (x 1000)
LiveDead FVS570
Not-internalized
Membrane order
CD14-
1.Monocytes
CD14 APC
CD3 PerCP-Cy5.5
CD3+CD56-
NKT-like T
4.NK CD56++
CD3-CD56-
3.NK
CD56 PerCP-eFluor 710
5.NKT-like CD8+
6.NKT-like CD8++
CD8 APC-Fire 810
7.B
2.Dendritic
CD19 Alexa Fluor 647
CD11c APC-R700
CD4+
16.DN CD4-CD8-
CD8+
CD4 NovaFluor Yellow 660
9.Effector CD4+
8.Naive CD4+
10.EM CD4+
11.CM CD4+
CD45RA APC-H7
CCR7 PE-Vio770
13.Effector CD8+
12.Naive CD8+
14.EM CD8+
15.CM CD8+
b
Sphingomyelinase gating strategy
c
Control
Sphingomyelinase
UMAP1
UMAP2
High
Low
Membrane order
1 Monocytes
2 Dendritic
3 NK
4 NK CD56++
5 NKT-like CD8+
6 NKT-like CD8-
7 B
8 Naive CD4+
9 Effector CD4+
10 EM CD4+
11 CM CD4+
12 Naive CD8+
13 Effector CD8+
14 EM CD8+
15 CM CD8+
16 DN CD4CD8+
d
Naive CD8+
Control
Sphingomyelinase
e
Control
Sphingomyelinase
f
Control
g
Sphingomyelinase
Monocytes
Dendritic
NK
NK CD56++
NKT-like CD8+
NKT-like CD8++
B
Naive CD4+
Effector CD4+
EM CD4+
CM CD4+
Naive CD8+
Effector CD8+
EM CD8+
CM CD8+
DN CD4-/CD8-

Supplementary fig. 10: Membrane order analysis of PBMCs treated with sphingomyelinase.

a–b, Spectral flow cytometry gating strategies used to identify viable singlet populations in **(a)** control and **(b)** sphingomyelinase-treated PBMCs. **c**, Cell type-dependent membrane order calculations, showing baseline differences among immune subsets. **d**, Example full-spectrum analysis of naive $CD8^+$ T cells, illustrating how single-cell membrane order can be resolved using the full emission profile of Pro12A. **e**, Comparison of membrane order across all PBMC subsets in control versus sphingomyelinase-treated conditions, highlighting cell type-dependent responses. Colors indicate replicate medians. Black lines indicate mean ± SD. **f–g**, Single-cell membrane order distributions per replicate in **(f)** control and **(g)** sphingomyelinase-treated PBMCs. White lines represent the median and interquartile range. Data are representative of three independent experiments. Statistical analysis was tested by two-way ANOVA; **** $p<0.0001$; ** $p<0.01$. Each sample includes measurements from at least fifteen thousand cells.

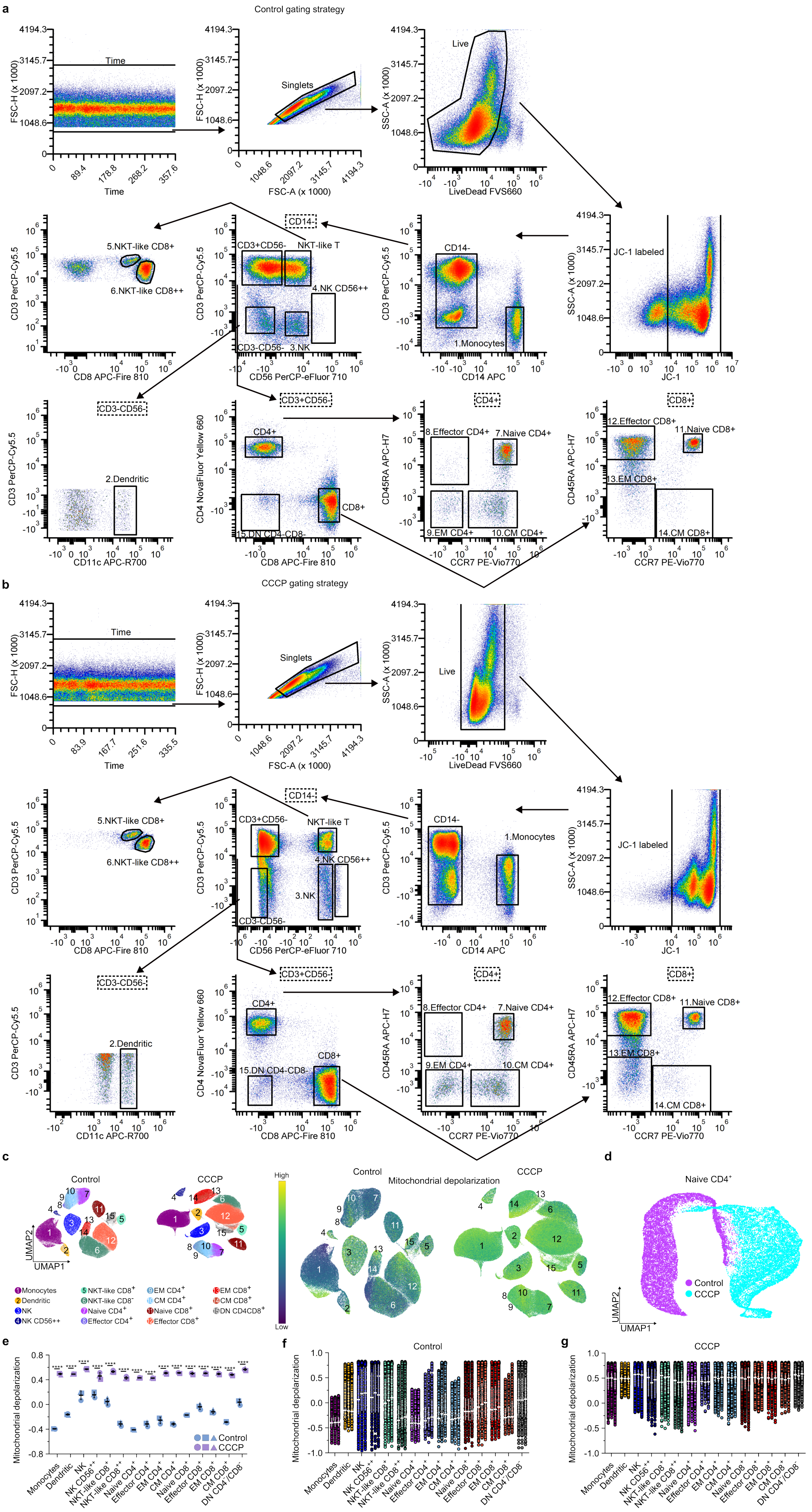

a
Control gating strategy
Time
FSC-H (x 1000)
Singlets
FSC-A (x 1000)
Live
SSC-A (x 1000)
LiveDead FVS660
JC-1 labeled
JC-1
CD14-
1.Monocytes
CD3 PerCP-Cy5.5
CD14 APC
CD14-
CD3+CD56-
NKT-like T
4.NK CD56++
CD3-CD56-
3.NK
CD56 PerCP-eFluor 710
5.NKT-like CD8+
6.NKT-like CD8++
CD8 APC-Fire 810
CD3-CD56-
2.Dendritic
CD11c APC-R700
CD3+CD56-
CD4+
CD8+
15.DN CD4-CD8-
CD4 NovaFluor Yellow 660
CD4+
8.Effector CD4+
7.Naive CD4+
9.EM CD4+
10.CM CD4+
CD45RA APC-H7
CCR7 PE-Vio770
CD8+
12.Effector CD8+
11.Naive CD8+
13.EM CD8+
14.CM CD8+
b
CCCP gating strategy
c
Control
CCCP
UMAP1
UMAP2
1 Monocytes
2 Dendritic
3 NK
4 NK CD56++
5 NKT-like CD8+
6 NKT-like CD8-
7 Naive CD4+
8 Effector CD4+
9 EM CD4+
10 CM CD4+
11 Naive CD8+
12 Effector CD8+
13 EM CD8+
14 CM CD8+
15 DN CD4CD8+
High
Low
Mitochondrial depolarization
d
Naive CD4+
Control
CCCP
e
Mitochondrial depolarization
Control
CCCP
Monocytes
Dendritic
NK
NK CD56++
NKT-like CD8+
NKT-like CD8++
Naive CD4+
Effector CD4+
EM CD4+
CM CD4+
Naive CD8+
Effector CD8+
EM CD8+
CM CD8+
DN CD4-/CD8-
f
Control
g
CCCP

Supplementary fig. 11: Mitochondrial depolarization analysis of PBMCs treated with CCCP.

a–b, Spectral flow cytometry gating strategies to identify viable singlet PBMCs in **(a)** control and **(b)** CCCP-treated samples. **c**, Cell type-dependent mitochondrial depolarization calculations, revealing baseline differences in mitochondrial membrane potential among immune subsets. **d**, Example full-spectrum analysis of naive $CD4^+$ T cells, demonstrating single-cell resolution of mitochondrial depolarization using JC-1 emission profiles. **e**, Comparison of mitochondrial depolarization across all PBMC subsets in control versus CCCP-treated conditions, highlighting cell type-dependent responses. Colors indicate replicate medians. Black lines indicate mean ± SD. **f–g,** Single-cell mitochondrial depolarization distributions per replicate in **(f)** control and **(g)** CCCP-treated PBMCs. White lines represent the median and interquartile range. Data are representative of three independent experiments. Statistical analysis was tested by two-way ANOVA; **** $p<0.0001$. Each sample includes measurements from at least fifteen thousand cells.

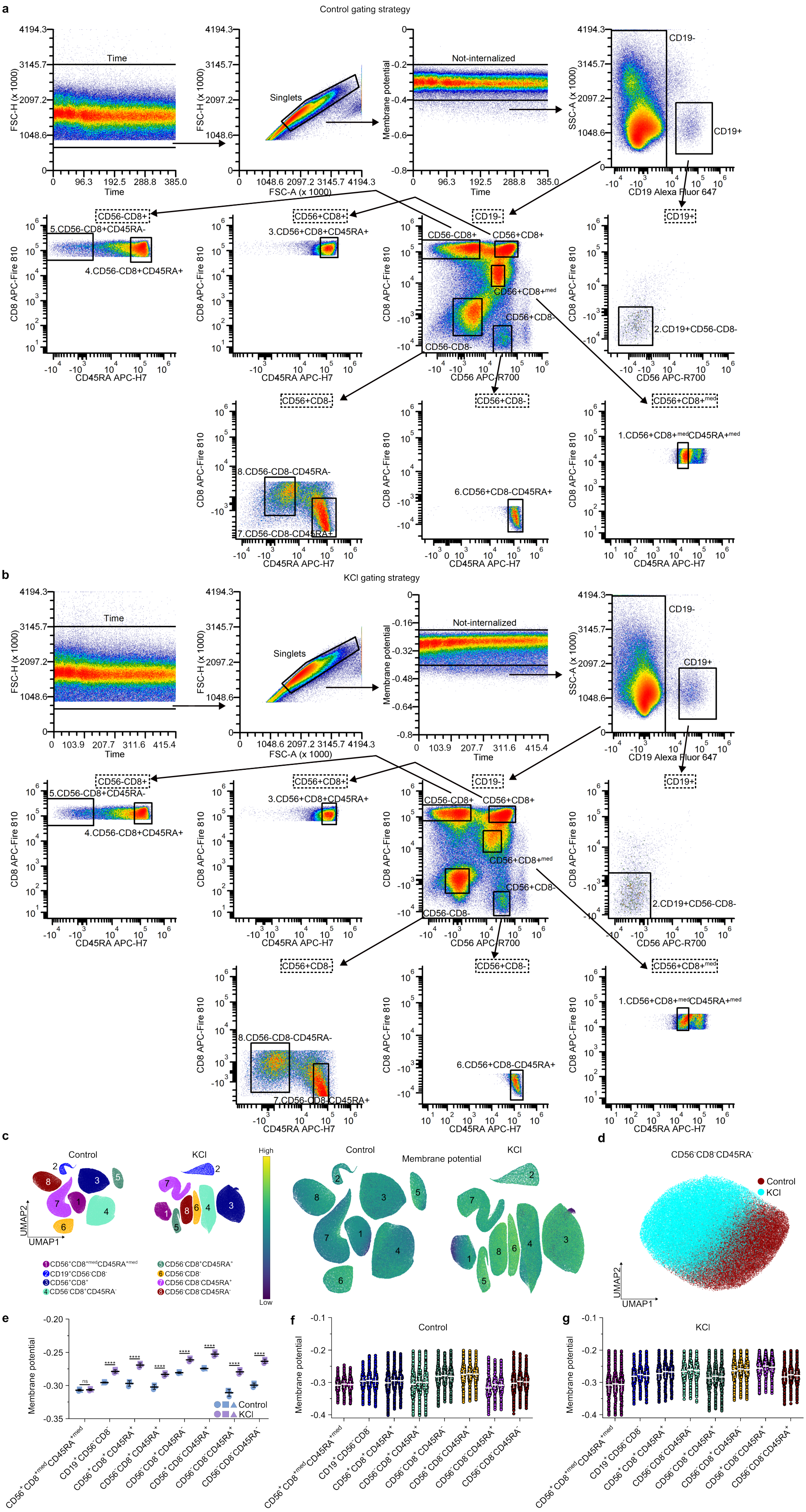

a
Control gating strategy
Time
Singlets
Not-internalized
CD19-
CD19+
FSC-H (x 1000)
FSC-A (x 1000)
SSC-A (x 1000)
Membrane potential
CD19 Alexa Fluor 647
CD56-CD8+
CD56+CD8+
CD56+CD8+med
CD56+CD8-
CD56-CD8-
5.CD56-CD8+CD45RA-
4.CD56-CD8+CD45RA+
3.CD56+CD8+CD45RA+
2.CD19+CD56-CD8-
8.CD56-CD8-CD45RA-
7.CD56-CD8-CD45RA+
6.CD56+CD8-CD45RA+
1.CD56+CD8+medCD45RA+med
CD8 APC-Fire 810
CD45RA APC-H7
CD56 APC-R700
b
KCl gating strategy
c
Control
KCl
UMAP1
UMAP2
Membrane potential
High
Low
CD56+CD8+medCD45RA+med
CD19+CD56-CD8-
CD56+CD8+
CD56-CD8+CD45RA+
CD56-CD8+CD45RA-
CD56+CD8-
CD56-CD8-CD45RA+
CD56-CD8-CD45RA-
d
CD56-CD8-CD45RA-
Control
KCl
e
Membrane potential
ns
****
Control
KCl
f
Control
g
KCl

Supplementary fig. 12: Membrane potential analysis of PBMCs incubated with KCl buffer.

a–b, Spectral flow cytometry gating strategies to identify viable singlet PBMCs in **(a)** control and **(b)** KCl-incubated conditions. **c**, Cell type-dependent membrane potential calculations, showing baseline differences among immune subsets and responses to KCl treatment. **d**, Example full-spectrum analysis of $CD8^{-}CD45RA^{-}$ cells, illustrating single-cell resolution of membrane potential using the CV1 probe. **e**, Comparison of membrane potential across all PBMC subsets in control versus KCl-incubated conditions, highlighting cell type-dependent responses. Colors indicate replicate medians. Black lines indicate mean ± SD. **f–g,** Single-cell membrane potential distributions per replicate in **(f)** control and **(g)** KCl-incubated PBMCs. White lines represent the median and interquartile range. Data are representative of three independent experiments. Statistical analysis was tested by two-way ANOVA; **** $p<0.0001$; ns, not significant. Each sample includes measurements from at least fifteen thousand cells.

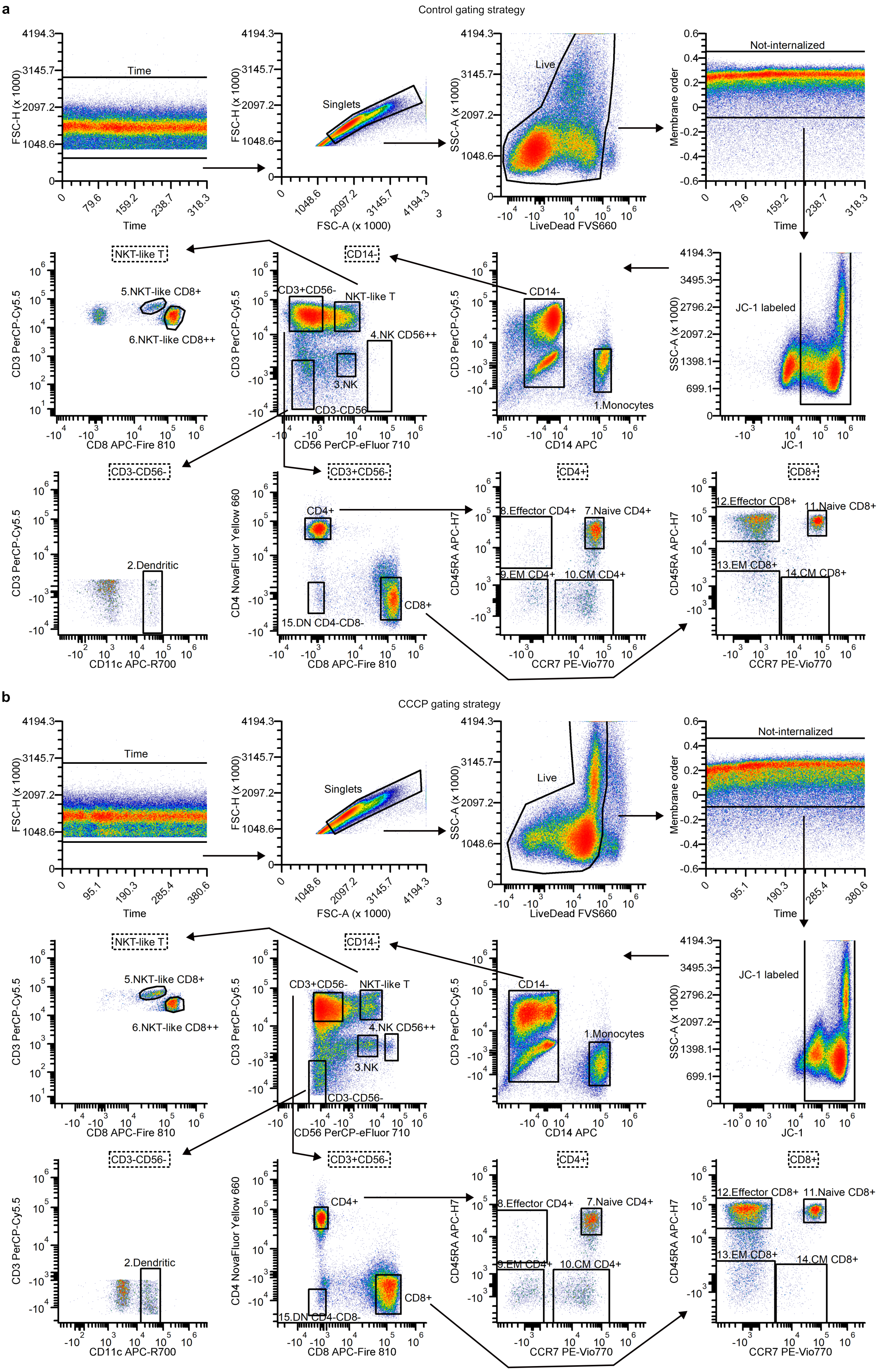

Supplementary fig. 13: Membrane order and mitochondrial depolarization analysis of PBMCs treated with CCCP v1.

a–b, Spectral flow cytometry gating strategies used to identify viable singlet PBMCs in **(a)** control and **(b)** CCCP-treated conditions for simultaneous analysis of membrane order (Pro12A) and mitochondrial depolarization (JC-1). Each sample includes measurements from at least fifteen thousand cells.

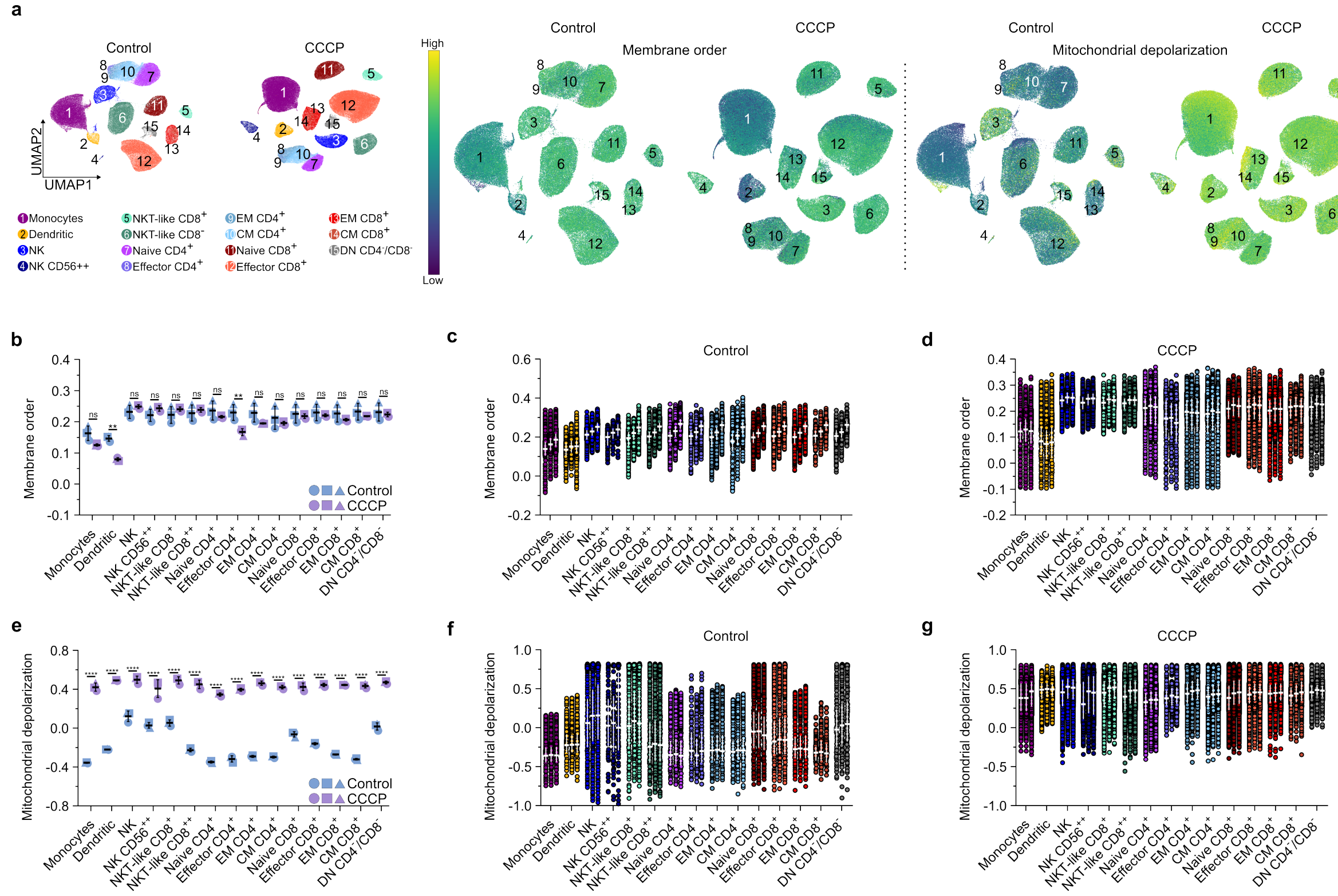

Supplementary fig. 14: Membrane order and mitochondrial depolarization analysis of PBMCs treated with CCCP v2.

a, Cell type-dependent calculations of membrane order (Pro12A) and mitochondrial depolarization (JC-1), highlighting baseline differences among immune subsets. **b**, Comparison of membrane order across all PBMC subsets in control versus CCCP-treated cells, showing cell type-dependent responses. Colors indicate replicate medians. Black lines indicate mean ± SD. **c–d,** Single-cell membrane order distributions per replicate in **(c)** control and **(d)** CCCP-treated PBMCs. White lines represent the median and interquartile range. **e**, Comparison of mitochondrial depolarization across all PBMC subsets in control versus CCCP-treated cells, illustrating differential responses among immune cell types. Colors indicate replicate medians. Black lines indicate mean ± SD. **f–g**, Single-cell mitochondrial depolarization distributions per replicate in **(f)** control and **(g)** CCCP-treated PBMCs. White lines represent the median and interquartile range. Data are representative of three independent experiments. Statistical analysis was tested by two-way ANOVA; **** $p<0.0001$; ** $p<0.01$; ns, not significant. Each sample includes measurements from at least fifteen thousand cells.

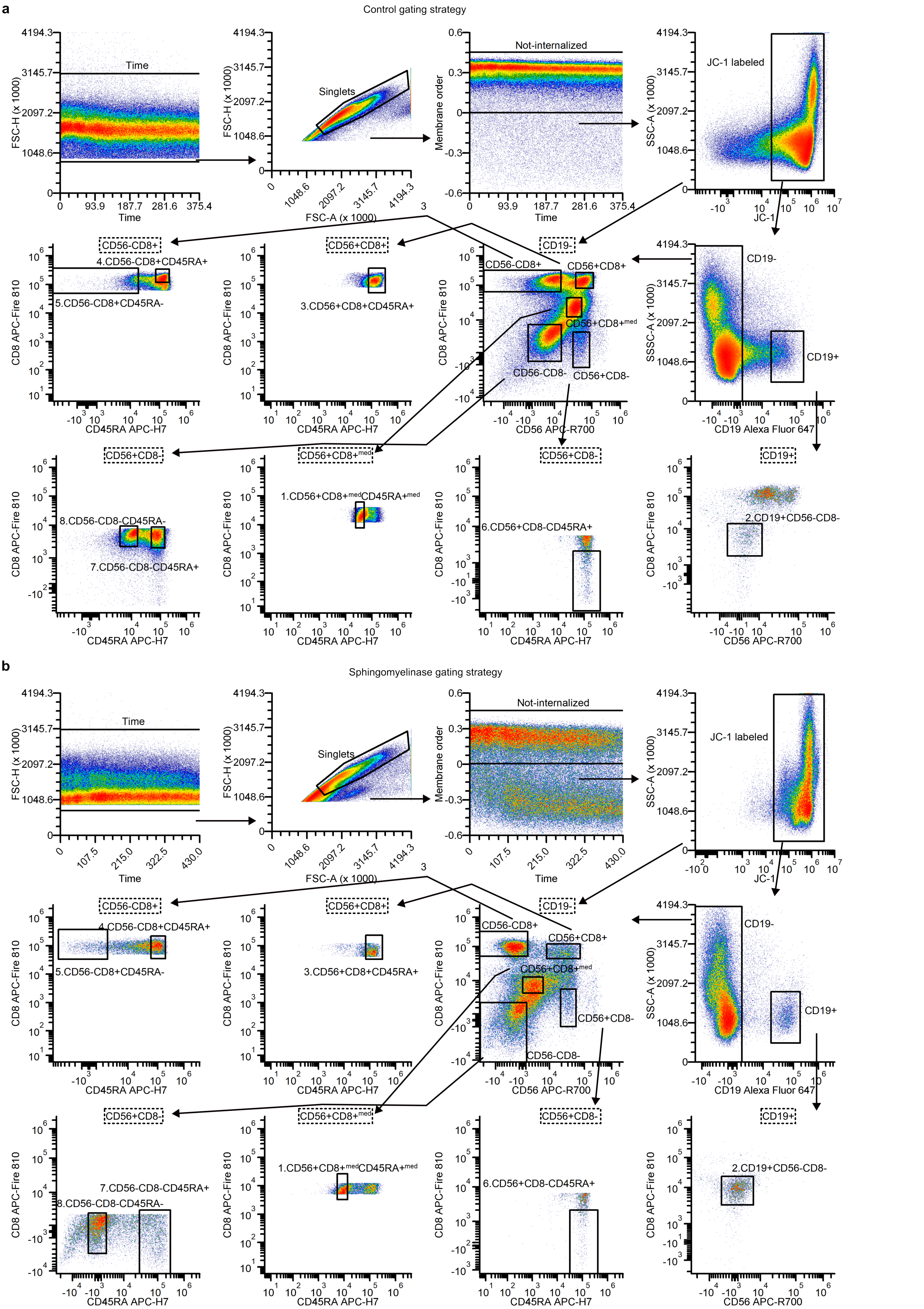

Supplementary fig. 15: Membrane order, mitochondrial depolarization, and membrane potential analysis of PBMCs treated with sphingomyelinase v1.
a–b, Spectral flow cytometry gating strategies used to identify viable singlet PBMCs in **(a)** control and **(b)** sphingomyelinase-treated conditions for simultaneous analysis

of membrane order (Pro12A), mitochondrial depolarization (JC-1), and membrane potential (CV1). Each sample includes measurements from at least fifteen thousand cells.

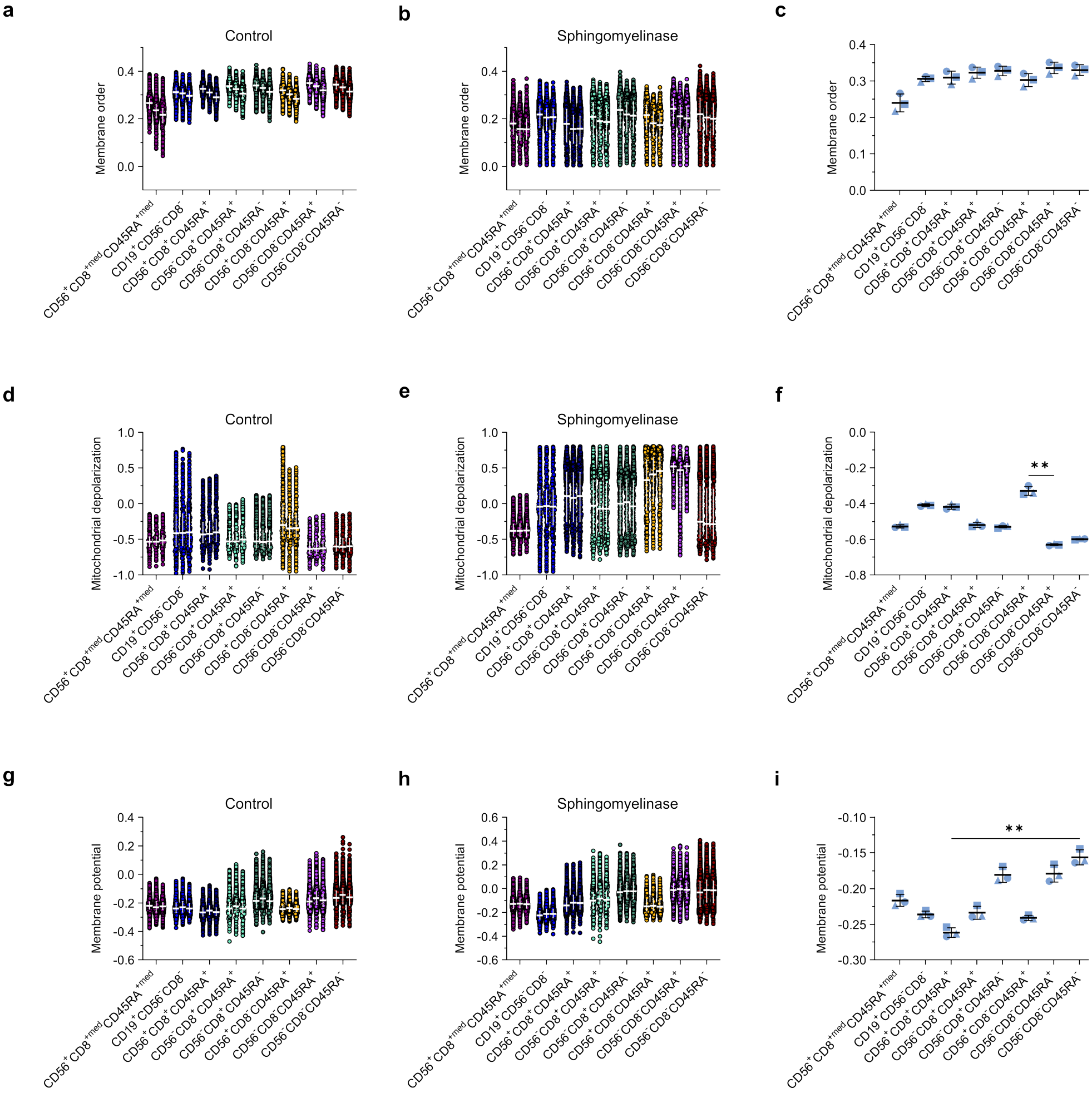

Supplementary fig. 16: Membrane order, mitochondrial depolarization, and membrane potential analysis of PBMCs treated with sphingomyelinase v2.

a–b, Single-cell membrane order distributions per replicate in **(a)** control and **(b)** sphingomyelinase-treated PBMCs, measured using Pro12A. **c**, Comparison of membrane order across all PBMC subsets in control, highlighting intrinsic heterogeneity between immune cell types. Colors indicate replicate medians. Black lines indicate mean ± SD. **d–e**, Single-cell mitochondrial depolarization distributions per replicate in **(d)** control and **(e)** sphingomyelinase-treated PBMCs, measured using JC-1. **f**, Comparison of mitochondrial depolarization across all PBMC subsets in control. **g–h**, Single-cell membrane potential distributions per replicate in **(g)** control and **(h)** sphingomyelinase-treated PBMCs, measured using CV1. **i**, Comparison of membrane potential across all PBMC subsets in control. White lines indicate the median and interquartile range. Data are representative of three independent experiments. Statistical significance was evaluated using a one-way nonparametric Kruskal–Wallis test: ** $p<0.01$, only significant ones are shown. Each sample includes measurements from at least fifteen thousand cells.

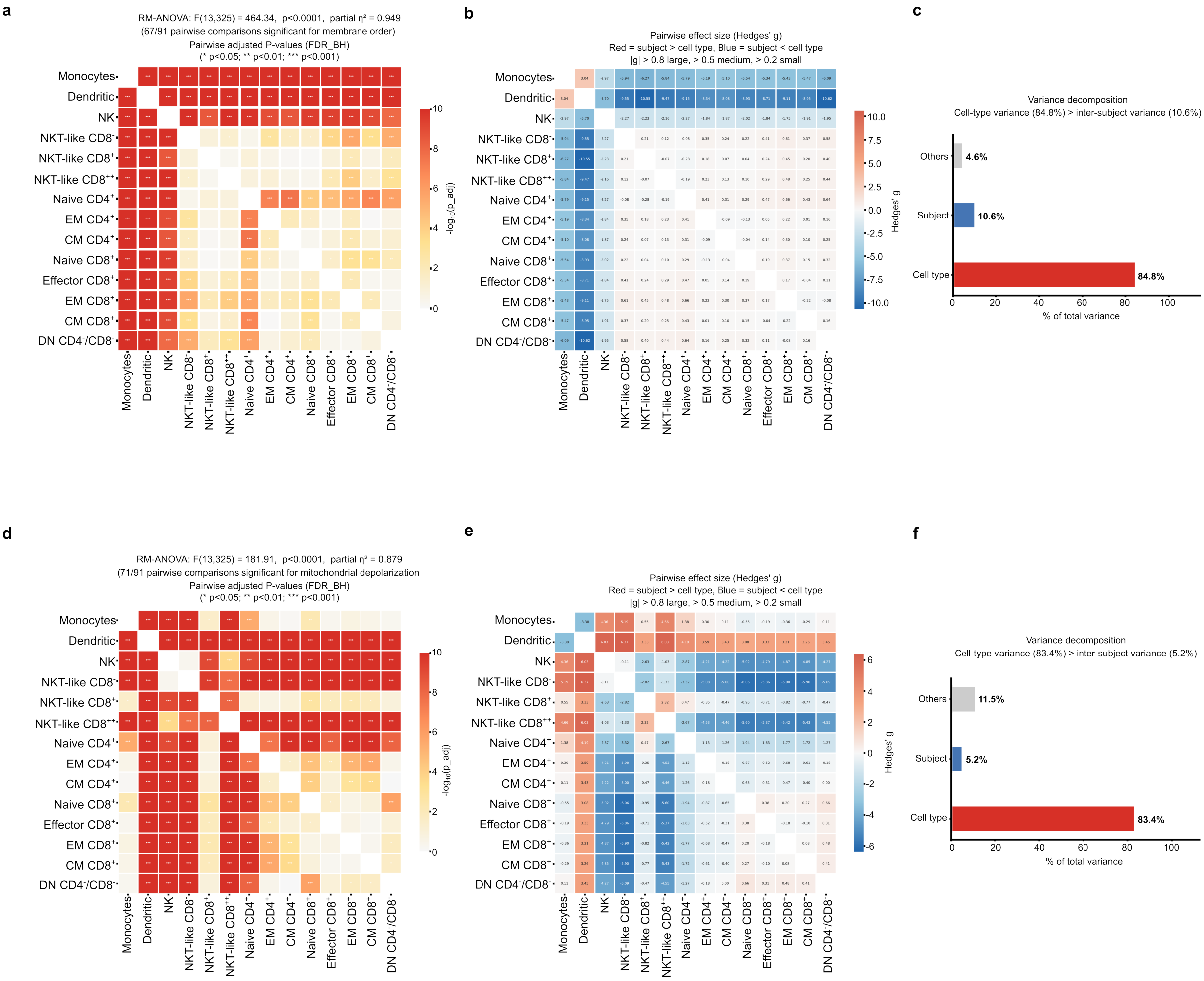

Supplementary fig. 17: Baseline biophysical heterogeneity across immune cell types in healthy donors.

a, Heatmap displaying pairwise adjusted p-values from post-hoc paired t-tests across all 14 cell types (91 comparisons). Cell colors represent $-\log_{10}$(adjusted p-value), with warmer colors indicating greater statistical significance. Asterisks denote significance thresholds: *$p < 0.05$, **$p < 0.01$, ***$p < 0.001$. P-values were corrected for multiple comparisons using the Benjamini-Hochberg false discovery rate procedure ($\alpha = 0.05$); 67 of 91 pairwise comparisons reached significance. Cell-type differences were first confirmed by repeated-measures one-way ANOVA with Greenhouse–Geisser correction for sphericity violation ($F(13,325) = 464.34$, $p < 0.0001$, partial $\eta^2 = 0.949$, generalized $\eta^2 = 0.848$). **b**, Heatmap showing the effect size (Hedges' g) for all pairwise comparisons between 14 cell types. Red indicates the row cell type has higher membrane order than the column cell type; blue indicates the inverse. Effect size thresholds: $|g| > 0.2$ small, > 0.5 medium, > 0.8 large. **c**, Total variance in membrane order was partitioned into three sources using a repeated-measures framework: variance attributable to cell type (between columns, 84.8%), variance attributable to inter-individual differences (between rows/subjects, 10.6%), and others (error and interaction, 4.6%). The dominant contribution of cell type confirms that observed differences are cell-type-dependent rather than driven by inter-subject variability. Overall cell-type effect: repeated-measures one-way ANOVA with Greenhouse–Geisser correction, $F(13,325) = 464.34$, $p < 0.0001$, partial $\eta^2 = 0.949$, generalized $\eta^2 = 0.848$; $n = 26$ healthy subjects. **d-f,** Heatmaps and total variance in mitochondrial depolarization. 71 of 91 pairwise comparisons reached significance ($F(13,325) = 181.91$, $p < 0.0001$, partial $\eta^2 = 0.879$, generalized $\eta^2 = 0.834$). While variance

attributable to cell type is 83.4%), variance attributable to inter-individual differences is 5.2%.

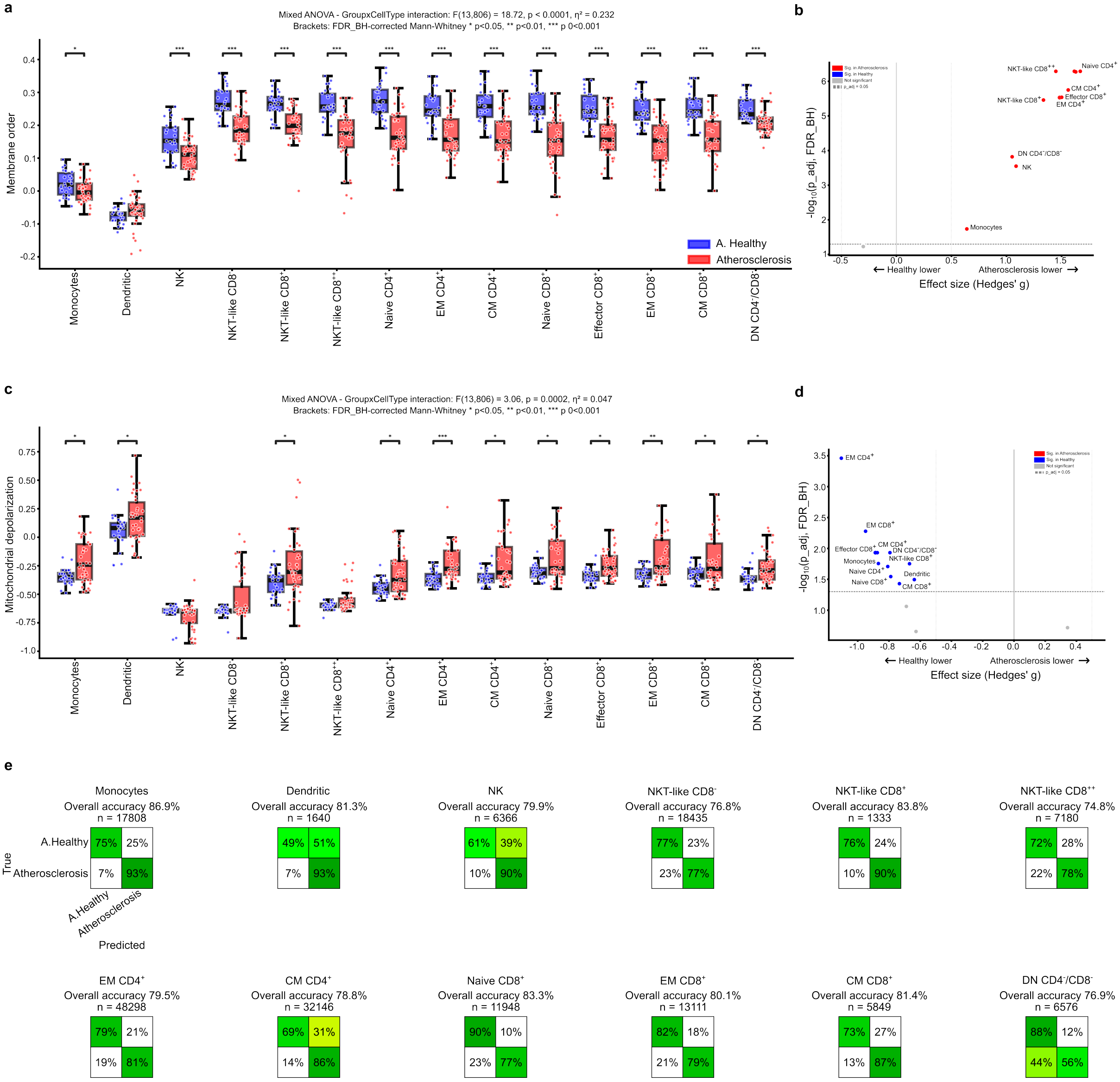

Supplementary fig. 18: Multi-parametric biophysical profiling of atherosclerosis by immune cell type.

a, Side-by-side boxplots of membrane order across 14 immune cell types comparing healthy donors (n = 26) and atherosclerotic individuals (n = 38). Brackets indicate pairwise significance (Mann-Whitney U test, Benjamini-Hochberg FDR correction, $\alpha = 0.05$). Group differences were assessed by two-way mixed ANOVA with group as the between-subject factor and cell type as the within-subject factor. A significant group and cell-type interaction confirmed that disease-associated membrane order changes are cell-type-dependent (group effect: $F(1,62) = 42.46$, $p < 0.0001$, partial $\eta^2 = 0.406$; cell-type effect: $F(13, 806) = 462.44$, $p < 0.0001$, partial $\eta^2 = 0.882$; interaction: $F(13,806) = 18.72$, $p < 0.0001$, partial $\eta^2 = 0.232$). **b**, Volcano plot summarizing per-cell-type group comparisons for membrane order. The x-axis shows effect size (Hedges' g; positive values indicate lower membrane order in atherosclerosis), and the y-axis shows $-\log_{10}$(adjusted p-value). Dashed horizontal line marks the significance threshold ($p_{adj} = 0.05$). Filled symbols indicate significance after Benjamini-Hochberg correction; 13 of 14 cell types reached significance. **c**, As in a, for mitochondrial depolarization. A significant group and cell-type interaction confirmed

cell-type-dependent remodeling (group effect: $F(1,62) = 15.85$, $p = 0.0002$, partial $\eta^2 = 0.204$; cell-type effect: $F(13, 806) = 200.93$, $p < 0.0001$, partial $\eta^2 = 0.764$; interaction: $F(13,806) = 3.06$, $p = 0.0002$, partial $\eta^2 = 0.047$). **d**, As in b, for mitochondrial depolarization; 11 of 14 cell types reached significance. **e**, Full-spectrum supervised classification by immune cell type, demonstrating accurate separation of cell populations based on biophysical signatures.

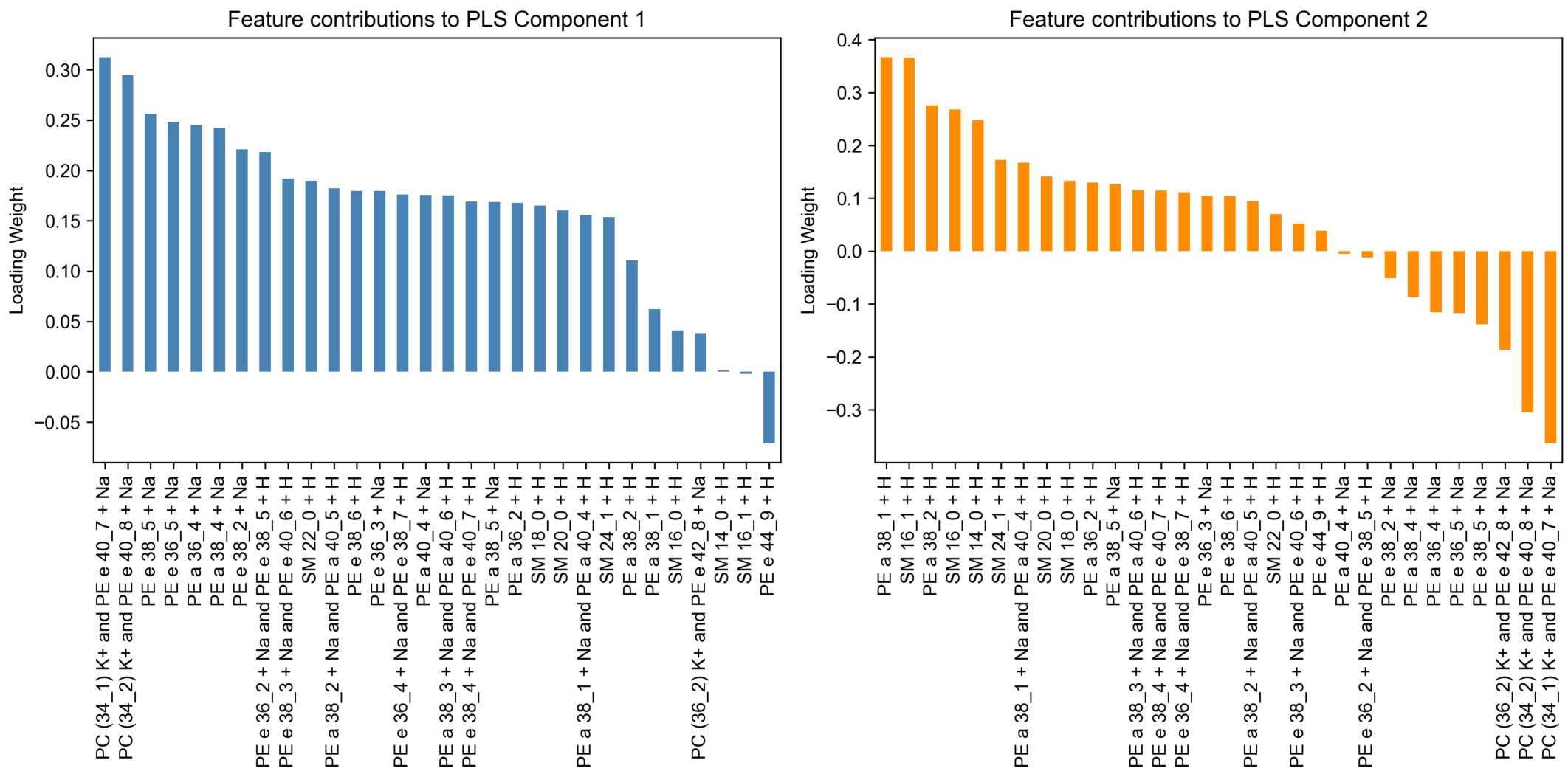

Supplementary fig. 19: Feature contributions to PLS components in atherosclerosis PBMC analysis.

This figure shows the contribution of individual lipidomic features to the principal components derived from partial least squares (PLS) regression. Each bar represents the weight of a specific feature in the corresponding PLS component, highlighting which variables drive separation between healthy and atherosclerosis donors. Features with larger absolute values contribute more strongly to the component.
