# Supplementary material for: Spectral biophysical cytometry with nanosensors reveals remodelling of immune cells in atherosclerosis
Source: Nat Nanotechnol. 2026 Aug 6;21(9):1308–15. doi: 10.1038/s41565-026-02236-8 (PMC13569417; doi:10.1038/s41565-026-02236-8)
Supplement: Supplementary file 2 [file 41565_2026_2236_MOESM2_ESM.pdf]

nature portfolio

Corresponding author(s): Erdinc Sezgin; Cenk O. Gurdap

Last updated by author(s): Apr 22, 2026

Reporting Summary

Nature Portfolio wishes to improve the reproducibility of the work that we publish. This form provides structure for consistency and transparency in reporting. For further information on Nature Portfolio policies, see our Editorial Policies and the Editorial Policy Checklist.

Statistics

For all statistical analyses, confirm that the following items are present in the figure legend, table legend, main text, or Methods section.

n/a	Confirmed	
☐	☒	The exact sample size (*n*) for each experimental group/condition, given as a discrete number and unit of measurement
☐	☒	A statement on whether measurements were taken from distinct samples or whether the same sample was measured repeatedly
☐	☒	The statistical test(s) used AND whether they are one- or two-sided<br>*Only common tests should be described solely by name; describe more complex techniques in the Methods section.*
☒	☐	A description of all covariates tested
☐	☒	A description of any assumptions or corrections, such as tests of normality and adjustment for multiple comparisons
☐	☒	A full description of the statistical parameters including central tendency (e.g. means) or other basic estimates (e.g. regression coefficient) AND variation (e.g. standard deviation) or associated estimates of uncertainty (e.g. confidence intervals)
☐	☒	For null hypothesis testing, the test statistic (e.g. *F*, *t*, *r*) with confidence intervals, effect sizes, degrees of freedom and *P* value noted<br>*Give P values as exact values whenever suitable.*
☒	☐	For Bayesian analysis, information on the choice of priors and Markov chain Monte Carlo settings
☒	☐	For hierarchical and complex designs, identification of the appropriate level for tests and full reporting of outcomes
☐	☒	Estimates of effect sizes (e.g. Cohen's *d*, Pearson's *r*), indicating how they were calculated

Our web collection on statistics for biologists contains articles on many of the points above.

Software and code

Policy information about availability of computer code

Data collection	Flow cytometry data were acquired using Cytek SpectroFlo software (v3.3.0). Confocal microscopy images were collected using the Zeiss Zen software (v2012 SP5). Sequencing was performed on an Illumina NovaSeq X Plus system (NovaSeq X Series Control Software v1.2.2.48004). Mass spectrometry was performed on an Orbitrap IQ-X (Thermo Fisher Scientific, San Jose, CA, USA) instrument. No proprietary or unpublished software was used during raw data collection aside from the instrument-native packages listed above.
Data analysis	Biophysical quantifications from microscopy images was performed using the previously published open-source software (https://www.nature.com/articles/s41596-024-01122-8; https://github.com/pcarravilla/fluiditycalculator/tree/main). Flow cytometry data were processed in FCS Express 7 (DeNovo Software, v7.28.0035). Sequencing data were processed with Cell Ranger (v9.0.1). Downstream analysis was conducted in R (v4.4.3) using Seurat (v5.2.1) pipeline. For mass spectrometry, raw data files were converted to .mzML using MSConvertGUI (ProteoWizard, v3.0.22285). For plotting and statistical analysis GraphPad Prism 10 (v10.6.1) was used. Figures and schematic was made in Biorender and Inkscape.

For manuscripts utilizing custom algorithms or software that are central to the research but not yet described in published literature, software must be made available to editors and reviewers. We strongly encourage code deposition in a community repository (e.g. GitHub). See the Nature Portfolio guidelines for submitting code & software for further information.

Data

Policy information about availability of data

All manuscripts must include a data availability statement. This statement should provide the following information, where applicable:

- Accession codes, unique identifiers, or web links for publicly available datasets
- A description of any restrictions on data availability
- For clinical datasets or third party data, please ensure that the statement adheres to our policy

All data is openly available in DOI: 10.17044/scilifelab.30406327

Research involving human participants, their data, or biological material

Policy information about studies with human participants or human data. See also policy information about sex, gender (identity/presentation), and sexual orientation and race, ethnicity and racism.

Reporting on sex and gender	Not Applicable
Reporting on race, ethnicity, or other socially relevant groupings	Not Applicable
Population characteristics	Not Applicable
Recruitment	Healthy donor samples were obtained from the Karolinska University Hospital biobank, while peripheral blood samples from individuals with atherosclerosis by clinical collaborators based on availability. No additional metadata were collected, as the aim of this study was to demonstrate the methodological capability of profiling biophysical measurements in immune cells rather than to perform cohort-level stratified analyses.
Ethics oversight	Not Applicable

Note that full information on the approval of the study protocol must also be provided in the manuscript.

Field-specific reporting

Please select the one below that is the best fit for your research. If you are not sure, read the appropriate sections before making your selection.

☒ Life sciences ☐ Behavioural & social sciences ☐ Ecological, evolutionary & environmental sciences

For a reference copy of the document with all sections, see nature.com/documents/nr-reporting-summary-flat.pdf

Life sciences study design

All studies must disclose on these points even when the disclosure is negative.

Sample size	No statistical methods were used to prospectively predetermine sample size. Instead, sample sizes were determined by the availability of donor material and the primary aim of the study, which was to establish and validate a methodological framework for biophysical measurements in human immune cells. A total of 26 healthy donors and 38 individuals with atherosclerosis were included, reflecting the number of high-quality samples that could be obtained from the corresponding clinical sources during the study period. Because the objective was to demonstrate proof-of-concept rather than to perform population-level comparisons or derive clinical associations, these sample sizes were considered sufficient to show technical feasibility, robustness of the measurement pipeline, and reproducibility of phenotyping across diverse immune cell types.
Data exclusions	Not Applicable
Replication	All key experimental findings were replicated across multiple independent biological samples. Standardized calibration procedures, such as daily flow cytometer check-ups and QC analysis, was performed to ensure technical consistency across acquisition days. All replication attempts were successful, and no experiments produced contradictory or irreproducible results, indicating that the observed effects are robust and reproducible across donors, batches, and experimental days.
Randomization	Not Applicable
Blinding	Not Applicable

Reporting for specific materials, systems and methods

We require information from authors about some types of materials, experimental systems and methods used in many studies. Here, indicate whether each material, system or method listed is relevant to your study. If you are not sure if a list item applies to your research, read the appropriate section before selecting a response.

Materials & experimental systems

n/a	Involved in the study
☐	☒ Antibodies
☐	☒ Eukaryotic cell lines
☒	☐ Palaeontology and archaeology
☒	☐ Animals and other organisms
☒	☐ Clinical data
☒	☐ Dual use research of concern
☒	☐ Plants

Methods

n/a	Involved in the study
☒	☐ ChIP-seq
☐	☒ Flow cytometry
☒	☐ MRI-based neuroimaging

Antibodies

Antibodies used

For Pro12A-only experiments, PBMCs were stained with the following antibodies: anti-human CD3-PerCP-Cy5.5 (clone SK7, BioLegend), CD56-PerCP-eFluor 710 (clone CMSSB, ThermoFisher), CD4-NovaFluor Yellow 660 (clone SK3, ThermoFisher), CD197 (CCR7)-PE-Vio770 (clone REA108|TG8, Miltenyi Biotec), CD14-APC (clone M5E2, BioLegend), CD19-Alexa Fluor 647 (clone HIB19, BioLegend), CD11c-APC-R700 (clone 3.9, BD Biosciences), CD45RA-APC-H7 (clone HI100, BD Biosciences), CD8a-APC-Fire 810 (clone SK1, BioLegend), and Fixable Viability Stain 570 (BD Biosciences). For JC-1-only and Pro12A–JC-1 combination experiments, the following antibodies were used: CD3-PerCP-Cy5.5, CD56-PerCP-eFluor 710, CD4-NovaFluor Yellow 660, CD197 (CCR7)-PE-Vio770, CD14-APC, CD11c-APC-R700, CD45RA-APC-H7, CD8a-APC-Fire 810, and Fixable Viability Stain 660 (BD Biosciences). For CV1-only and Pro12A–JC-1–CV1 combination experiments, the antibody panel included: CD56-PerCP-eFluor 710, CD19-Alexa Fluor 647, CD45RA-APC-H7, and CD8a-APC-Fire 810.

Validation

Validation of primary antibodies was performed by the manufacturers, as reported on their websites.

Eukaryotic cell lines

Policy information about cell lines and Sex and Gender in Research

Cell line source(s)

The CEM cell line were purchased from ATCC.

Authentication

None of the cell lines were authenticated.

Mycoplasma contamination

Cell lines were tested for mycoplasma contamination.

Commonly misidentified lines
(See ICLAC register)

None of the cell lines were authenticated.

Plants

Seed stocks

Report on the source of all seed stocks or other plant material used. If applicable, state the seed stock centre and catalogue number. If plant specimens were collected from the field, describe the collection location, date and sampling procedures.

Novel plant genotypes

Describe the methods by which all novel plant genotypes were produced. This includes those generated by transgenic approaches, gene editing, chemical/radiation-based mutagenesis and hybridization. For transgenic lines, describe the transformation method, the number of independent lines analyzed and the generation upon which experiments were performed. For gene-edited lines, describe the editor used, the endogenous sequence targeted for editing, the targeting guide RNA sequence (if applicable) and how the editor was applied.

Authentication

Describe any authentication procedures for each seed stock used or novel genotype generated. Describe any experiments used to assess the effect of a mutation and, where applicable, how potential secondary effects (e.g. second site T-DNA insertions, mosiacism, off-target gene editing) were examined.

Flow Cytometry

Plots

Confirm that:

☒ The axis labels state the marker and fluorochrome used (e.g. CD4-FITC).

☒ The axis scales are clearly visible. Include numbers along axes only for bottom left plot of group (a 'group' is an analysis of identical markers).

☒ All plots are contour plots with outliers or pseudocolor plots.

☒ A numerical value for number of cells or percentage (with statistics) is provided.

Methodology

Sample preparation

More detailed description written in the method part of the paper.

PBMCs (covered by ethical permit Dnr 2025-01255-01, 2025-02-20) were isolated from 45mL of buffy coat obtained from healthy donors. The buffy coat was diluted 1:1 with PBS and evenly distributed into two 50mL Falcon tubes. After thorough mixing with a serological pipette, 30mL of the diluted sample was carefully layered over 15mL of Ficoll-Paque (Sigma-Aldrich) in three separate 50mL tubes. Samples were centrifuged at 400×g for 25 minutes without applying the brake. The PBMC layer was collected and transferred into fresh tubes, followed by two PBS washes (300×g, 5 minutes, brake on). After the final wash, cells were resuspended in 20mL PBS, filtered through a 70µm cell strainer (ThermoFisher), and centrifuged at 200g for 10 minutes. The supernatant was discarded, and the cell pellet was resuspended in cryopreservation medium (90% FBS, 10% DMSO). Cells were aliquoted (1mL per vial) and stored at –150°C until use.
For experiments, frozen PBMCs were thawed by gradual addition of a 10-fold volume of pre-warmed RPMI-1640 medium supplemented with 10% FBS, followed by centrifugation at 400g for 6 minutes. Cells were processed in Eppendorf tubes at a final concentration of 6×105 cells per condition. For mitochondrial depolarization assays, cells were first incubated with JC-1 dye for 30 minutes at 37°C, followed by two PBS washes. Cells were then treated with CCCP for 5 minutes to induce mitochondrial depolarization and washed twice with PBS. For membrane order analysis, cells were treated with sphingomyelinase (Sigma-Aldrich) for 1 h at 37°C and subsequently washed twice with PBS. While antibody staining was performed after treatments and JC-1 labeling for mitochondrial depolarization, it was done before Pro12A or/and CV1 labeling.
Following treatments, cells and their untreated controls were used to prepare both single-stained controls and multi-color antibody panels for spectral flow cytometry. Antibody titrations were optimized based on staining index calculations. Each sample was either unstained, stained with a single antibody or probe (for spectral unmixing) or with a full antibody cocktail together with probe/s in staining buffer (ThermoFisher). Staining was performed at 4°C in the dark for 30 minutes, followed by two PBS washes. Cells were then resuspended in 500µL PBS, and viability was assessed by adding 1µL of a Live/Dead dye (1:100 dilution), incubating for 15 minutes at room temperature in the dark, followed by two final PBS washes. Cells were then resuspended in 300µL PBS in flow tubes for acquisition.
For Pro12A-only experiments, PBMCs were stained with the following antibodies: anti-human CD3-PerCP-Cy5.5 (clone SK7, BioLegend), CD56-PerCP-eFluor 710 (clone CMSSB, ThermoFisher), CD4-NovaFluor Yellow 660 (clone SK3, ThermoFisher), CD197 (CCR7)-PE-Vio770 (clone REA108|TG8, Miltenyi Biotec), CD14-APC (clone M5E2, BioLegend), CD19-Alexa Fluor 647 (clone HIB19, BioLegend), CD11c-APC-R700 (clone 3.9, BD Biosciences), CD45RA-APC-H7 (clone HI100, BD Biosciences), CD8a-APC-Fire 810 (clone SK1, BioLegend), and Fixable Viability Stain 570 (BD Biosciences). For JC-1-only and Pro12A–JC-1 combination experiments, the following antibodies were used: CD3-PerCP-Cy5.5, CD56-PerCP-eFluor 710, CD4-NovaFluor Yellow 660, CD197 (CCR7)-PE-Vio770, CD14-APC, CD11c-APC-R700, CD45RA-APC-H7, CD8a-APC-Fire 810, and Fixable Viability Stain 660 (BD Biosciences). For CV1-only and Pro12A–JC-1–CV1 combination experiments, the antibody panel included: CD56-PerCP-eFluor 710, CD19-Alexa Fluor 647, CD45RA-APC-H7, and CD8a-APC-Fire 810.

Instrument

Cytek Aurora [Spectral Flow Cytometry] 5 lasers

Software

SpectroFlo

Cell population abundance

Whole population of peripheral blood mononuclear cells without an enrichment

Gating strategy

More detailed description written in the method part of the paper and provided as a supplementary. In short:
Flow cytometry data were analyzed by first gating on time, non-debris, singlets, and live cells. After these initial steps, further gating was performed according to the specific markers that define each cell population, described in detailed in supplementary figures.

☒ Tick this box to confirm that a figure exemplifying the gating strategy is provided in the Supplementary Information.
