# Supplementary material for: Spectral biophysical cytometry with nanosensors reveals remodelling of immune cells in atherosclerosis
Source: Nat Nanotechnol. 2026 Aug 6;21(9):1308–15. doi: 10.1038/s41565-026-02236-8 (PMC13569417; doi:10.1038/s41565-026-02236-8)
Supplement: Supplementary file 3 [file 41565_2026_2236_MOESM3_ESM.pdf]

nature portfolio

Peer Review File

Spectral biophysical cytometry with nanosensors reveals remodeling of immune cells in atherosclerosis

Corresponding Author: Dr Erdinc Sezgin

Version 0:

Reviewer comments:

Reviewer #1

(Comments for the Author)
SUMMARY OF THE KEY RESULTS:
The manuscript written by Gurdap et al. reports the use of environment-sensitive fluorescence probes on a commercial spectral flow cytometer to detect multiple biophysical properties of individual cells, alongside standard biochemical properties. Specifically, one probe that the author previously reported and two commercially available probes are used to assess membrane packing, membrane potential, and mitochondrial potential. Machine learning was applied to the full-spectrum profiles to achieve probing sensitivity superior to conventional ratiometric measurements.
The effectiveness of these probes was validated across various sample types with proper perturbations, including engineered microbeads, drug-treated cell lines, and human peripheral blood cells subjected to drug treatments. This biophysical profiling was then integrated with immune phenotyping to investigate differences in the properties of human blood-derived immune cells under two conditions: (1) normal versus treatment with sphingomyelinase, and (2) cells extracted from healthy individuals versus those with atherosclerosis. With the aid of immunophenotyping, the authors reported not only significant changes in cell biophysical properties in both cases but also a subtype dependency of these properties. These findings support the assay's applicability in monitoring physiologically relevant changes and its potential as a biomarker for disease screening. The later finding is further studied and explained by mass spectrometry and single-cell RNA sequencing.

ORIGINALITY AND SIGNIFICANCE
Notably, probing multiple physiologically relevant biophysical properties at high throughput has been a challenge in the field of biophysical cytometry, but the authors' approach, using a panel of reported/existing fluorophores with high-dimensional analysis, may be regarded as a straightforward approach in the field of flow cytometry. The clinical study is the first showing that atherosclerosis is associated with a change in membrane potential and packing of immune cells, with scRNAseq and mass spectrometry to support the observation and explain its origin.

DATA & METHODOLOGY:
The methodology is sound, and the use of flow cytometry and collected data meets the required standard. However, the cell-type specificity of the biophysical properties used is not fully supported by the data analysis.

REFERENCES: APPROPRIATE CREDIT TO PREVIOUS WORK?
The work below, which presents the use of a fluorescence tag in combination with flow cytometry to probe the biophysical properties of cells at high throughput, is relevant to this work.
- Ma, Rong, et al. "Molecular mechanocytometry using tension-activated cell tagging." Nature methods 20.11 (2023): 1666-1671.

CLARITY AND CONTEXT
The reported method to probe biophysical properties of single cells is clearly presented.

APPROPRIATE USE OF STATISTICS AND TREATMENT OF UNCERTAINTIES
The number of replicates, samples, and cell count is sufficient. More statistical analysis could be applied to better justify the cell-type specificity of the biophysical properties used.

CONCLUSIONS: ROBUSTNESS, VALIDITY, RELIABILITY
The cell-type specificity of the biophysical properties used requires more justification.

COMMENTS AND SUGGESTED IMPROVEMENTS

1. The reported subtype dependency of the biophysical properties used is very interesting; however, it requires more analysis to substantiate this claim. Specifically:
- The authors should analyse the composition of the cluster and present the result in the form of a confusion matrix for PBMCs treated with sphingomyelinase, comparing PBMCs from healthy donors versus those from atherosclerosis patients. This approach would allow for the evaluation of the specificity of the biophysical properties by comparing them to antibody staining, which is the gold standard.
- UMAP plots color-coded by subtypes could be presented in multiple plots, each color-coded by only one subtype, to clearly show the distribution among clusters. The composition of the cluster can hardly be clearly visualized in a single plot due to the dense packing of data points.
- Even if the biophysical properties are subtype specific, the change of these properties may not be. The authors stated that,

"Interestingly, all three parameters changed in treated cells, but in a cell-type specific manner (Supplementary Fig. 1-15, 1-16)"

"Interestingly, we observed cell type-specific remodeling of biophysical properties, with T cells being the most affected."

"Extending the analysis to other immune cell types, we generated 2D feature plots across all measured subsets (Fig. 2e), along with UMAP and classification results
(Supplementary Fig. 2-1) confirming cell type-specific biophysical alterations in
atherosclerosis."

However, the authors did not analyze the relationship between the change in the properties and the cell subtype. The bar plots given in the main figures or supplementary figures show that the change of biophysical properties varies among subtypes, but not supportive enough to claim subtype-specific.
2. The authors report that the living status of cells significantly influences membrane order and propose this property as a biomarker to distinguish live from dead cells. However, without live/dead staining or dead cells as controls, how can the analysis and gating be ensured to avoid misleading results due to cell death? For example, in supplementary Fig. 1-13 and 1-15.
3. How was the threshold of membrane potential being set to discriminate live and dead cells without live/dead staining or any control where the membrane order is not an absolute value?
4. The observation of high predictive accuracy for distinguishing healthy and atherosclerotic conditions in both naïve CD4+ and effector CD8+ populations is not sufficiently supported by the analysis. The tables in Figures 2c and 2d only indicate that cells from healthy and atherosclerosis subjects exhibit different biophysical properties. In the analysis of assay accuracy, the unit should be a subject rather than a cell. The authors should first define a classifier and demonstrate its classification accuracy using a confusion matrix at the subject level.
5. The gating strategy should also be provided for the clinical data.

MINOR COMMENTS:
1. Please correct Supplementary Fig.1-2h to Supplementary Fig 1-2g.
2. In Method, Drug treatments on CEM cells, there is an incomplete sentence.
"…in the flow cytometry tubes immediately before the. For mitochondrial …"
3. In Method, PBMCs with atherosclerosis, there is a typo.
"…After the final wash, cells were cryopreserved at 1 × 106 cells/cryovial in freezing medium (90% FBS + 10% DMSO). …"

Reviewer #2

(Comments for the Author)
The authors describe Spectral Biophysical Cytometry (SBC) as a high-throughput method that integrates spectral flow cytometry with environment-sensitive nano sensors to measure multiple biophysical properties of cells simultaneously. They claim that unlike traditional tools, which analyse one cell or a single parameter at a time, SBC enables multi-parametric profiling of millions of cells at single-cell resolution. It further captures features such as membrane order, tension, deformability, and mitochondrial potential, providing richer insights into cellular physiology. They finally demonstrated in immune cells from individuals with atherosclerosis, SBC revealed disease-specific biophysical remodelling. This platform offers new opportunities for diagnostics, prognosis, and advancing our understanding of how biophysical states govern health and disease.
The here presented approach is a robust and good addition to the growing number of single cell analysis, particularly since it is taking membrane properties into account and will be beneficial not only for cell biology and physiology but also for translational research in general. However, the SBC methodology is not fundamentally novel but rather combines existing methods such as membrane biosensors with cytometry. The manuscript is overall concisely written but lacks clarity in many parts. While the cytometry is well done and nicely detailed, i.e. the lipidomics lacks a bit of precision and detail (see comments below). Once these comments are addressed, I consider this manuscript suitable for publication in nature nanotechnology.

Major Points:
- The manuscript in general is lacking important explanations i.e. as to why specific drugs were chosen, and how they impact particularly membrane properties. This is (to give only one example here) reflected in using the term "Membrane drug" which is not telling anything about the nature of the drug nor its impact on the membrane. Here one would wish for a

more detailed description and reasoning.
- There is a complete lack of microscopic data showing or confirming the phenotypes described in the paper (i.e. for the membrane probes CV1 and JC1)
- In the part about the immune cells, it is not clear why the authors use the disrupted systems and do not comment on differences in healthy state between cell lines
- The part about Atherosclerosis also lacks reasoning missing as to why they chose this model
- The figures are generally not very coherent, particularly regarding the colour scheme. It would be great to have a coherent colour scheme throughout the manuscript or at least within one figure. In this figure sometimes the same condition has a different colour depending on the figure panel, which makes it hard to follow.
- The figures in general lack statistics.

Minor Points: Main Text
- The figure legends in general are not detailed and informative enough
- Supplementary Figure 1-1: What is on the x-axis, this is nowhere explained, I guess this is channels, but this needs an explanation particularly for a broader readership.
- Supplementary Figure 1-2: It is difficult to understand the reasoning for the probe Pro12A. From the figure it is also not clear how the changes of membrane properties lead to a chromatic shift of the dyes, but rather a change in signal intensity, but this might become clearer, when it is clear what is plotted on the x-axis. Then, panel h should be panel g.
- Figure 1 k: There is at least a comment missing for a better understanding of the findings
- Figure 1 l: It is not clear what the numbers in the UMAPs represent.
- The authors mention that T-cells are most affected, however, there is no comment on the exact markers they mention, which makes it currently not possible to link the text to the figure since the labelling in figure (CD4+/CD8+ etc) is not linked no comment on T-cell origin of those.
- Figure 2c and d – it is not clear what the middle UMAP panel is supposed to convey
- Figure 2e: It would be desirable to include a more detailed interpretation of the data. It would further be interesting to explore if there are specific donors where all cell lines overlap with the healthy control or whether different atherosclerosis patients show different cell line affects. In other words, would it be possible to identify based on the cell types defined subsets of Atherosclerosis patients with specific cell lines affected? Finally, please mark in 2e those patients shown in 2f (where are those patients across cell lines?)
- Figure 2g: The text refers to lipid classes while plot is a PCA which does not show lipid classes, creating a disconnect between the text and the figure. It would be helpful to either include a figure displaying the described effect on lipid classes in the main or the supplementary or limit the explanations directly linking to the clustering of the PCA
- Figure 2g/h: It would be desirable to see a plot with not only ratios but abundances for the different lipid classes in healthy vs diseased cells.
- There is a mention in the text that the authors have seen “strong correlation between theoretically calculated membrane potential and the ratiometric fluorescence signal validating CV1 as a reporter for membrane potential in non-excitable cells” However, there is no corresponding figure to support this finding.
- Figure 2 i: it is unclear where these genes come from and where the raw data is, since there is none shown or referenced.
- It is not clearly explained why the authors treat the PBMCs with sphingomyelinase
- Regarding the scRNA data, the sequencing quality control data missing from supplement

Version 1:

Reviewer comments:

Reviewer #1

(Comments for the Author)
Comments 1 and 3 have not been sufficiently addressed in the authors’ response. The explanations provided do not resolve my concerns, and the manuscript requires further revision to improve clarity and rigor.

1. Regarding the authors’ response to my comment, “Even if the biophysical properties are subtype specific, the change of these properties may not be.”, I assume that the Fig. 1m stated in the following comments corresponds to Fig. 2f-g.

“…In addition, we added the statistics and delta values (mean difference) for representing the difference in biophysical properties between conditions (Fig. 1m), which is discussed in the next point below.”

“…To this end, we added “delta membrane order” values on the graphs (Fig. 1m) for each cell type, so that it is immediately clear to the reader that different cell types are affected differently by drug treatment.”

I do not see how these two sub-figures provide additional support for the “cell type-dependent” claim. Firstly, it is not analytically meaningful to compare each patient's data to the mean of healthy data. Secondly, the variation in the difference of membrane order and depolarization between patients appears visually more significant than that between cell types (i.e., variation along rows vs. variation along columns). To validate whether cell–type–dependent differences truly exist, a proper statistical analysis should be performed to determine whether the observations are statistically significant.

2. The specificity and effectiveness of membrane order as a viability marker must be vigorously validated to substantiate the claims in the manuscript.

Line 100-101, "…Notably, we found that Pro12A is also effective for distinguishing live and dead cells…."

It is clear that the membrane order is significantly influenced by various other parameters, as shown in Figs. 1and 2, spanning a broad range from -0.6 to 1. Notably, the membrane order shown in the Supplementary Fig. 1-3 falls well within the range – indicating NO absolute threshold that reliably identifies dead cells within a heterogeneous population, especially in complex clinical samples. Hence, a quantitative and rigorous analysis should be conducted to compare with the live/dead stain to demonstrate its validity for the blood sample.

Reviewer #2

(Comments for the Author)
I would like to thank the authors for addressing all previously raised points and for providing the additionally requested data.

Minor Comments

• In the introduction, several sentences - particularly from line 72 onward - are very long and could be made more concise. Furthermore, after "(Fig. 1a)." the full stop appears to be incorrectly placed.

• Regarding the suggestion for coherent colors: using an inclusive color palette is appreciated. However, colors should still remain coherent. For example, in Fig. 1e the color used for the 18:1–18:1 lipid should match the pink used for this composition in Fig. 1b and remain distinct from the purple used for 16:1–16:1. The same applies to Fig. 1c. Keeping colors consistent across panels for the same lipid composition makes the figures easier to follow.

• Figure 1e, the main text, and Supplementary Figures 1–2e: the wording "Lipid tails length" should be revised. It should either be "tail length" or "tail's length," but ideally it should be replaced with "acyl chain length."

• Supplementary Figures 1–2: thank you for adding the arrow to highlight the difference of interest. However, it remains unclear what the essential information is e.g., is it the differential y-value of differently ordered compositions? Additionally, while the colors between panels a and d are consistent, the colors between panels d and e are not. Improving color coherence across panels would help the reader.

• Supplementary Figure 1–3: statistical information is missing. Displaying non-significant changes as well would strengthen the point being made.

• Figure 2c & d (left) and corresponding text around line 280: the use of the term "clear" may be overstated given that the groups partially overlap.

• Figure 2h (in relation to f & g): outlining the further-analyzed samples greatly improved clarity - thank you for adding this. It may also be helpful to indicate which specific patient (patient numbers as in panels f & g) each highlighted data point corresponds to, especially now that an overall trend per patient is visible in the heatmaps.

• A further thought on Fig. 2h: selecting the four samples per condition that are most clearly separated is entirely reasonable. Still, it could be interesting to explore whether lipidomics or transcriptomics alone can discriminate the overlapping areas. This does not need to be addressed; it is more of a conceptual suggestion.

• Another consideration regarding Fig. 2c–k: if available, integrating physiological disease severity into the separation could be informative. Mapping disease severity onto the analysis space might reveal inherent trends, such as clearer separation with increasing severity, and may even allow visualization of disease progression.

Version 2:

Reviewer comments:

Reviewer #1

(Comments for the Author)
The authors have made substantial improvements and have addressed my previous concerns in a precise, thorough, and satisfactory manner. I also appreciate the authors' efforts in carefully revising the manuscript and responding to the reviewers' comments. In particular:

1. Analytical validity
The methodology has been clarified, and additional supporting data have been provided, strengthening the overall rigor of the approach.

2. Statistical analysis for cell-type dependency
The revised analysis is more rigorous and better justified, increasing confidence in the reported distinctions between cell

types.

3. Validation of membrane order as a viability marker
The added validation experiments and clarifications adequately support the authors’ claims and improve the credibility of this aspect of the study.

In light of these revisions, I believe that the manuscript would be a valuable contribution to the field and is suitable for publication.

Open Access This Peer Review File is licensed under a Creative Commons Attribution 4.0 International License, which permits use, sharing, adaptation, distribution and reproduction in any medium or format, as long as you give appropriate credit to the original author(s) and the source, provide a link to the Creative Commons license, and indicate if changes were made.
In cases where reviewers are anonymous, credit should be given to 'Anonymous Referee' and the source.
The images or other third party material in this Peer Review File are included in the article’s Creative Commons license, unless indicated otherwise in a credit line to the material. If material is not included in the article’s Creative Commons license and your intended use is not permitted by statutory regulation or exceeds the permitted use, you will need to obtain permission directly from the copyright holder.
To view a copy of this license, visit https://creativecommons.org/licenses/by/4.0/

We sincerely thank the reviewers and the editor for their thorough evaluation of our manuscript and for their constructive feedback. We believe that the revisions have strengthened the manuscript and made it more accessible to a broader audience. Below, we provide detailed responses to each comment along with the corresponding changes made in the manuscript.

Editorial requests:

Data Availability

As per our institutional guidelines, all data will be openly available in Figshare DOI: 10.17044/scilifelab.30406327

Further experimental data, controls and data analysis

As detailed below, we added new data in Fig 2 and several supplementary figures to clarify our findings.

Novelty against membrane tension biosensors

Rong et al mentioned by the Reviewer 1 (cited in the revised manuscript) uses conventional flow cytometry for the identification of mechanically active cells. Therefore, this application is very different than our methodology both technically and biologically. While their aim is to see mechanically strained cell surface, we achieve biophysical phenotyping of multiple cell types simultaneously through smart probes designed for organelle specific readout. We cited this manuscript and discussed our method against the other methods using mechanical sensors.

Reviewer #1:

DATA & METHODOLOGY:

The methodology is sound, and the use of flow cytometry and collected data meets the required standard. However, the cell-type specificity of the biophysical properties used is not fully supported by the data analysis.

We addressed this below in the comments section and added the modifications to the manuscript.

REFERENCES: APPROPRIATE CREDIT TO PREVIOUS WORK?

The work below, which presents the use of a fluorescence tag in combination with flow cytometry to probe the biophysical properties of cells at high throughput, is relevant to this work.

- Ma, Rong, et al. "Molecular mechanocytometry using tension-activated cell tagging." Nature methods 20.11 (2023): 1666-1671.

We apologize for missing out on this important manuscript, we cited it in the revised manuscript

Lines 59-68:

‘Recent studies have demonstrated the utility of integrating single biophysical parameter, such as membrane order[6,7] or forces exerted through adhesion receptors[8], with or without surface marker profiling with conventional flow cytometry to reveal previously undetected cell states. However, most current methods are: *i)* custom-built; *ii)* constrained by their single parameter design; *iii)* low sensitivity and resolution due to one-detector-per-fluorochrome approach; and *iv)* not readily compatible with immune phenotyping pipelines. This limits both cell-type specificity and the ability to integrate diverse biophysical properties. As a result, our understanding of how biophysical states vary across diverse cell types and how these features collectively contribute to health and disease remains limited.’

APPROPRIATE USE OF STATISTICS AND TREATMENT OF UNCERTAINTIES

The number of replicates, samples, and cell count is sufficient. More statistical analysis could be applied to better justify the cell-type specificity of the biophysical properties used.

We put statistics to all graphs possible.

CONCLUSIONS: ROBUSTNESS, VALIDITY, RELIABILITY

The cell-type specificity of the biophysical properties used requires more justification.

We addressed this below in the comments section and added the modifications to the manuscript.

COMMENTS AND SUGGESTED IMPROVEMENTS

1. The reported subtype dependency of the biophysical properties used is very interesting; however, it requires more analysis to substantiate this claim. Specifically:

- The authors should analyse the composition of the cluster and present the result in the form of a confusion matrix for PBMCs treated with sphingomyelinase, comparing PBMCs from healthy donors versus those from atherosclerosis patients. This approach would allow for the evaluation of the specificity of the biophysical properties by comparing them to antibody staining, which is the gold standard.

We thank the reviewer for raising this point. We realize that our original explanation of the analysis may not have been sufficiently clear. To clarify, the definition of cell types in our study was not based on biophysical properties. Instead, we established cell-type identity using a classical gating strategy with well-established antibody combinations, exactly as the reviewer suggested. The UMAP projections were applied solely for visualization of antibody-defined clusters, providing a broader, single-cell representation of the biophysical data. Consequently, the composition of each cluster is already well defined through manual gating, as presented in the supplementary figures for each PBMC-related analysis.

We have clarified this point in the revised manuscript and figure legends. We also changed all PBMC related figures in a way that shows cell clustering first and then biophysical projection on top.

Lines 227-229:

‘Our analysis strategy was to generate UMAP projections of manual gating based on antibodies for cell-type clusters and then overlay biophysical properties for each cluster with single-cell resolution.’

We also appreciate the reviewer’s suggestion of employing a confusion matrix. While in the current study the manual gating approach is sufficient, we agree that such a strategy will be particularly valuable in future work where we aim to multiplex larger biomarker panels. In those contexts, when manual gating becomes less practical, the application of a confusion matrix will indeed be highly useful. If we have misunderstood the reviewer’s concern, we would be grateful for further clarification so that we may address it more directly in the revision.

- UMAP plots color-coded by subtypes could be presented in multiple plots, each color-coded by only one subtype, to clearly show the distribution among clusters. The composition of the cluster can hardly be clearly visualized in a single plot due to the dense packing of data points.

We assume that composition concern is again regarding the first comment. Hope it is now clearer with revised version.

We use current plotting strategy since it allows one to appreciate both the within-cluster and between-cluster distributions as well as their biophysical counterparts. For this reason, we chose to present the more complex UMAP projection in the main figures, complemented by 2D plots in the main and supplementary materials for clarity.

To make it even clearer, we showed cell clustering first and then biophysical projection on top **(Fig. 1l and Fig. 2b).** In addition, we added the statistics and delta values (mean difference) for representing the difference in biophysical properties between conditions **(Fig. 1m)** which is discussed in the next point below.

- Even if the biophysical properties are subtype specific, the change of these properties may not be. The authors stated that,

“Interestingly, all three parameters changed in treated cells, but in a cell-type specific manner (Supplementary Fig. 1-15, 1-16)” “Interestingly, we observed cell type-specific remodeling of biophysical properties, with T cells being the most affected.” “Extending the analysis to other immune cell types, we generated 2D feature plots across all measured subsets (Fig. 2e), along with UMAP and classification results (Supplementary Fig. 2-1) confirming cell type-specific biophysical alterations in atherosclerosis.”

However, the authors did not analyze the relationship between the change in the properties and the cell subtype. The bar plots given in the main figures or supplementary figures show that the change of biophysical properties varies among subtypes, but not supportive enough to claim subtype-specific.

We agree that the wording “**cell type specific**” is not fully accurate. While we see different effect on different cells, we still see an effect on every cell type, therefore the effect is “**cell type dependent**” rather than “cell type specific”. Therefore, we changed this wording to “cell type dependent”. We also made this cell type dependent effect clearer in the figures. To this end, we added “delta membrane order” values on the

graphs **(Fig 1m)** for each cell type, so that it is immediately clear to the reader that different cell types are affected differently by drug treatment.

2. The authors report that the living status of cells significantly influences membrane order and propose this property as a biomarker to distinguish live from dead cells. However, without live/dead staining or dead cells as controls, how can the analysis and gating be ensured to avoid misleading results due to cell death? For example, in supplementary Fig. 1-13 and 1-15.

First, we need to clarify that, we included live/dead staining alongside our membrane order probe in all experiments, including those shown in Supplementary Fig. 1-13 (as part of the gating strategy), except for measurements using CV1 (due to its broad red spectrum). Therefore, we revised the text accordingly to avoid any misunderstanding that we bypass the live/dead stain.

Having said that, as shown in Supplementary Fig. 1-3b–d, our membrane order probe performs comparably to conventional live/dead staining in detecting non-viable cells. This aspect of our probe is very useful because there is typically a delay between live/dead staining-washing and running the cells on the cytometer. During this interval, some cells that were not initially labeled by the live/dead dye may undergo cell death, whereas the membrane order probe (applied immediately before acquisition) can readily detect these cells.

To further minimize dead cells, we routinely assessed cell viability by counting prior to each experiment and consistently worked with samples of high viability, both for cultured cells and PBMCs. In those cases, membrane order, together with light scatter (as dead cells display distinct size distributions), provided a reliable means of discriminating live from dead cells. We agree that this is an important point, and we have clarified it in the revised text.

Lines 106-112:

'Nevertheless, to ensure robustness, we included conventional viability staining alongside membrane order probe in all experiments, except in measurements using membrane potential dye due to its broad red spectrum. In these cases, the combination of membrane order and light scatter (since dead cells display distinct size distributions) provided a reliable means of discriminating live from dead cells. Importantly, all experiments were performed under optimized culturing conditions, and our later analyses of donor samples consistently started with the highest possible viability.'

3. How was the threshold of membrane potential being set to discriminate live and dead cells without live/dead staining or any control where the membrane order is not an absolute value?

As explained above, we always included live/dead staining alongside our membrane order probe in all experiments, except for measurements using CV1 for membrane potential measurements due to the spectral properties of CV1. Being aware of this limitation, we did not use membrane potential probe in clinical data shown in the paper.

For the healthy cell measurements where membrane potential was used and combined with membrane order probe, threshold for discriminating live and dead cells with our membrane order probe was not defined arbitrarily. As shown in supplementary Fig. 1-3b-d, we directly compared membrane order readouts with conventional live/dead staining and found a near-complete overlap between the two approaches. This benchmarking allowed us to establish the range of probe ratios corresponding to dead cells. Importantly, dead cells consistently exhibit a pronounced and reproducible shift toward significantly lower membrane order values, reflecting disrupted membranes and probe internalization, which provides a clear biological separation from live cells. To further ensure robustness, we routinely performed cell counting and viability assessment prior to experiments, and we worked with highly viable cultures and clinical PBMC samples. In addition, forward and side scatter parameters were used as an orthogonal measure during the gating strategy, as dead cells display distinct scatting profiles.

Taken together, these measures allowed us to set thresholds confidently and reproducibly without relying exclusively on viability dyes, while also minimizing the risk of misclassification. Due to the spectral limitation of CV1, we did not use this probe in clinical data; however, we are actively working to improve the method by evaluating additional probes with diverse biophysical properties and narrower spectral profiles. This will allow us to identify more optimal probes for multiplexed measurements, reduce spectral overlap, and further enhance the robustness and applicability of our approach in future studies.

4. The observation of high predictive accuracy for distinguishing healthy and atherosclerotic conditions in both naïve CD4+ and effector CD8+ populations is not sufficiently supported by the analysis. The tables in Figures 2c and 2d only indicate that cells from healthy and atherosclerosis subjects exhibit different biophysical properties. In the analysis of assay accuracy, the unit should be a subject rather than a cell. The authors should first define a classifier and demonstrate its classification accuracy using a confusion matrix at the subject level.

We thank the reviewer for raising this important point. We agree that subject-level accuracy is the appropriate clinical unit. Our present study was a proof-of-concept single-cell investigation and is not powered to yield a robust subject-level classifier. The cohort sizes (26 healthy, 38 atherosclerotic) are too small to reliably train and validate machine-learning models without a substantial risk of overfitting. Additionally, we observe biologically plausible within-subject heterogeneity (some healthy donors contain cells with disease-associated phenotypes and vice versa), which underscores the utility of single-cell resolution but complicates direct translation to a binary subject label.

We have added a statement to the manuscript clarifying that the present study demonstrates predictive modelling for cell-level differences, but not subject-level prediction.

Lines 285-290:

'However, our current cohort (26 healthy, 38 atherosclerosis) was designed for proof-of-concept discovery at the single-cell level and is not powered to support definitive subject-level diagnostic models. Small cohort sizes substantially increase the risk of overfitting and yield wide uncertainty in machine-learning performance estimations for subject-level prediction. Thus, we only explored cell level prediction power of the method.'

We are currently exploring different disease models with our method and would like to establish larger cohorts where we can implement subject-level classifier in the future.

5. The gating strategy should also be provided for the clinical data.

We thank reviewer for pointing it out. We applied a similar gating strategy as described in Supplementary Fig. 1-13, from singlets and live-dead to cell-typing. We added this to the revised manuscript.

Lines 271-273:

'We applied the similar gating strategy we established in drug-treated PBMCS with Pro12A and JC-1 **(Supplementary fig.1-13)**.'

MINOR COMMENTS:

1. Please correct Supplementary Fig.1-2h to Supplementary Fig 1-2g.

2. In Method, Drug treatments on CEM cells, there is an incomplete sentence.

"…in the flow cytometry tubes immediately before the. For mitochondrial …"

3. In Method, PBMCs with atherosclerosis, there is a typo.

"…After the final wash, cells were cryopreserved at 1 × 106 cells/cryovial in freezing medium (90% FBS + 10% DMSO). …"

All minor mistakes are fixed.

Reviewer #2:

The manuscript is overall concisely written but lacks clarity in many parts. While the cytometry is well done and nicely detailed, i.e. the lipidomics lacks a bit of precision and detail (see comments below). Once these comments are addressed, I consider this manuscript suitable for publication in nature nanotechnology.

We thank the reviewer for their constructive comments. We addressed their concerns as detailed below.

Major Points:

- The manuscript in general is lacking important explanations i.e. as to why specific drugs were chosen, and how they impact particularly membrane properties. This is (to give only one example here) reflected in using the term "Membrane drug" which is not

telling anything about the nature of the drug nor its impact on the membrane. Here one would wish for a more detailed description and reasoning.

We thank the reviewer for pointing this out. We agree that our original text did not sufficiently explain the rationale for drug selection. In the revised manuscript, we now provide a more detailed description of the chosen compounds, their molecular targets, and their impact on membrane organization and biophysical properties.

Lines 114-130:

’Under optimized staining conditions, we treated CEM cells with a panel of compounds selected to perturb distinct cellular structures known to influence membrane organization **(Fig.1f)**. We selected drugs that can act on actin cytoskeleton, membrane lipids and glycocalyx. While all these structures are crucial for membrane integrity and function, it is not clear how they contribute to distinct biophysical properties. Actin cytoskeleton drugs (CK-666, an Arp2/3 inhibitor, and latrunculin B, which sequesters actin monomers) were used to alter cortical actin dynamics that directly support membrane structure. Plasma membrane lipid composition was perturbed by methyl-β-cyclodextrin (extracting cholesterol) and sphingomyelinase (hydrolyzing sphingomyelin) which target two important lipids for membrane properties. To probe the role of the glycocalyx, we included oseltamivir (a neuraminidase inhibitor that preserves sialylated glycans) and swainsonine (an α-mannosidase inhibitor that interferes with N-glycan maturation), thereby altering surface glycan composition. Across all drug treatments, we observed a consistent decrease in membrane order, showing the impact of these structural elements on physical properties of membranes and indicating the sensitivity of Pro12A to biophysical perturbations induced by various cellular structures.’

Lines 135-151:

’To enable multi-parametric biophysical phenotyping and uncover underexplored inter-parameter correlations, we integrated two additional environment-sensitive probes, JC-1 (mitochondrial depolarization) and CV1 (membrane potential)[12]. Using JC-1, we monitored mitochondrial depolarization in cells treated with carbonyl cyanide 3-chlorophenylhydrazone (CCCP, a protonophore uncoupler) or digitonin (which permeabilizes plasma membrane without notable damage to intracellular membranes and disrupts ionic homeostasis) **(Fig.1g, Supplementary fig.1-7)**. JC-1 accumulates in mitochondria in a potential-dependent manner: at high membrane potential (lower depolarization), it forms red-fluorescent aggregates, and at low potential (higher depolarization), it remains as green-fluorescent monomers. As expected, depolarized cells displayed an increased JC-1 monomer signal. For membrane potential analysis, cells were modulated with valinomycin, a potassium ionophore enabling the selective transport of potassium ions (K^+) in combination with varying extracellular K^+/Na^+ ratios to calibrate CV1 responses according to the Nernst equation. We observed a strong correlation between the theoretically calculated membrane potential (shown in x-axis) and the ratiometric fluorescence signal, validating CV1 as a reporter of membrane potential in non-excitable cells **(Fig.1h, Supplementary fig.1-8)**.’

- There is a complete lack of microscopic data showing or confirming the phenotypes described in the paper (i.e. for the membrane probes CV1 and JC1)

We agree with the reviewer that microscopic validation is important. For JC-1, we presented supplementary fig.1-7a for microscopic data. We further explained it in the revised manuscript.

Lines 137-144:

Using JC-1, we monitored mitochondrial depolarization in cells treated with carbonyl cyanide 3-chlorophenylhydrazone (CCCP, a protonophore uncoupler) or digitonin (which permeabilizes plasma membrane without notable damage to intracellular membranes and disrupts ionic homeostasis) **(Fig.1g, Supplementary fig.1-7)**. JC-1 accumulates in mitochondria in a potential-dependent manner: at high membrane potential (lower depolarization), it forms red-fluorescent aggregates, and at low potential (higher depolarization), it remains as green-fluorescent monomers.

For CV1, however, we were limited by detector sensitivity in microscopes. The CV1 probe emits in the near-infrared range, where the detector arrays available in standard commercial confocal systems (e.g., Zeiss LSM series) have reduced quantum efficiency. In contrast, the Cytek Aurora flow cytometer employs avalanche photodiodes, which provide better sensitivity at long wavelengths and allowed us to have a higher resolution in flow cytometry. Nevertheless, we have included a representative microscopy image of CV1-labeled cells (Supplementary fig.1-8) and cited the original publication that describes this probe in detail. In parallel, we are developing and testing new probes optimized for use on widely available imaging platforms, so that non-expert users without custom microscopes can apply them more easily with this method in the future.

- In the part about the immune cells, it is not clear why the authors use the disrupted systems and do not comment on differences in healthy state between cell lines

We thank the reviewer for pointing this out. We added the explanation on our goal for using disrupted systems in revised manuscript.

Lines 216-224:

'Our goal was to integrate immune phenotyping with biophysical profiling to assess how different immune cell types exhibit distinct biophysical signatures in health versus disease. We first focused on perturbed immune cells to assess how different cell types respond to membrane-related challenges and to evaluate the sensitivity of our system **(Fig.1k)**. By applying well-defined cellular perturbations, we could determine whether SBC can detect biophysical changes at the single-cell level in a cell type-dependent manner. This approach provides a benchmark for the performance of the method before extending it to clinical or more heterogeneous samples.'

We also added a reasoning for why we observed difference in healthy states between cell types.

Lines 251-255:

‘We also observed differences between untreated immune cell types. Consistent with recent lipidomic analyses14, immune cells exhibit complex and distinct lipid compositions, which are likely reflected in their varying membrane order and their response to lipid-modifying drugs.‘

- The part about Atherosclerosis also lacks reasoning missing as to why they chose this model

We revised the manuscript to make the reasoning clearer:

Lines 258-268:

‘Following the validation of SBC on drug-treated PBMCs, we next integrated immune phenotyping with biophysical profiling to differentiate apparently healthy individuals from those with atherosclerosis **(Fig.2a)**. Atherosclerosis is a multifactorial disease marked by endothelial dysfunction, dysregulated lipid metabolism, chronic inflammation, and sustained immune activation[14]. Recent reports elucidate that the circulating immune cells are central to both the initiation and progression of atherosclerotic lesions in a cell type dependent manner[15]. While transcriptomic and proteomic features of these cells have been studied to reveal the role of immune niches in cardiovascular diseases, their biophysical phenotypes remain poorly characterized, largely due to a lack of high-throughput tools capable of resolving immune cell heterogeneity at the single-cell level.’

- The figures are generally not very coherent, particularly regarding the colour scheme. It would be great to have a coherent colour scheme throughout the manuscript or at least within one figure. In this figure sometimes the same condition has a different colour depending on the figure panel, which makes it hard to follow.

We thank the reviewer for this comment to make the figures more understandable. While we chose the color schemes in our figures with accessibility in mind (including for color-blind readers) and we tried to optimize it for clarity within each experiment; it is really challenging to make it consistent across the entire manuscript or a figure since we are dealing with various different parameters in different panels in a single figure. For example, bead categories are consistently grouped by chemical property (e.g., saturation, double bond position), and their colors overlap with their counterparts in the corresponding UMAP plots. Similarly, probe-related drug treatments were grouped by functional category (e.g., actin, membrane, glycocalyx drugs share one color scheme, whereas mitochondrial and membrane potential perturbations use distinct palettes). Finally, multi-parametric experiments were assigned yet another scheme to help distinguish them clearly. While we think current color design of the figures is the optimal for accessibility given the multi-parameters in figures, we would be happy to revise the scheme (e.g., use monochromatic graphs for all) if the reviewer still considers our scheme confusing.

- The figures in general lack statistics.

We put statistics to all graphs possible.

Minor Points: Main Text

- The figure legends in general are not detailed and informative enough

We have now expanded the legends throughout the manuscript including the supplementary figures to include more detailed descriptions of the experimental setup, probe/drug treatments, gating strategies, and key observations shown in each panel.

- Supplementary Figure 1-1: What is on the x-axis, this is nowhere explained, I guess this is channels, but this needs an explanation particularly for a broader readership.

We apologize for overlooking this; we have now explicitly labeled x-axis and y-axis as “channel” in the first relevant legend and clarified this in the corresponding figure legend. We also added supplementary info, showing corresponding channel wavelengths in spectral flow setup.

- Supplementary Figure 1-2: It is difficult to understand the reasoning for the probe Pro12A. From the figure it is also not clear how the changes of membrane properties lead to a chromatic shift of the dyes, but rather a change in signal intensity, but this might become clearer, when it is clear what is plotted on the x-axis. Then, panel h should be panel g.

We totally agree that it was confusing in the last version. To clarify the interpretation of Pro12A in supplementary fig. 1-2, we have added arrows to the figure indicating the direction of membrane order (left = more ordered, right = more disordered). The x-axis represents spectral detection channels, which correspond to emission wavelength ranges. We also added channels’ corresponding wavelength as supplementary info.

We also corrected the labeling of panels.

- Figure 1 k: There is at least a comment missing for a better understanding of the findings

We made it clearer in the figure legend that it is a schematic of the following experiment.

- Figure 1 l: It is not clear what the numbers in the UMAPs represent.

We made it clearer in the figure legend.

- The authors mention that T-cells are most affected, however, there is no comment on the exact markers they mention, which makes it currently not possible to link the text to the figure since the labelling in figure (CD4+/CD8+ etc) is not linked no comment on T-cell origin of those.

We made the transition clearer.

Line 275-277:

’Interestingly, we observed cell type-dependent remodeling of biophysical properties, with T cells overall, including $CD4^+$ and $CD8^+$ subsets and their respective subtypes, showing the most pronounced changes.’

- Figure 2c and d – it is not clear what the middle UMAP panel is supposed to convey

We agree with the reviewer that it did not add extra information to the figure, therefore decided to remove it from the figure.

- Figure 2e: It would be desirable to include a more detailed interpretation of the data. It would further be interesting to explore if there are specific donors where all cell lines overlap with the healthy control or whether different atherosclerosis patients show different cell line affects. In other words, would it be possible to identify based on the cell types defined subsets of Atherosclerosis patients with specific cell lines affected? Finally, please mark in 2e those patients shown in 2f (where are those patients across cell lines?)

We thank the reviewer for this valuable suggestion. First, we modified the figure and marked the selected individuals for omics for each panel in the revised figure. This makes the reader see the biophysical properties of each cell type for the donors that we used for omics analysis.

Concerning the patient-specific cell type changes: it is a fantastic idea. We included the new figures in the revised manuscript **(Fig. 2f, g)**. In short, median differences of each patient for each cell type was calculated using median of all healthy individuals.

Line 294-301:

'To further examine inter-individual variability, we visualized patient-specific deviations in membrane order and mitochondrial potential across all 14 immune cell populations **(Fig. 2f, g)**. This representation revealed distinct biophysical signatures per donor, illustrating that although consistent cell type-level trends are apparent, the magnitude and pattern of remodeling vary between individuals. Together, these findings highlight the selective and personalized nature of disease-associated biophysical changes and underscore the value of SBC in uncovering both shared and individual-dependent biomarkers.'

- Figure 2g: The text refers to lipid classes while plot is a PCA which does not show lipid classes, creating a disconnect between the text and the figure. It would be helpful to either include a figure displaying the described effect on lipid classes in the main or the supplementary or limit the explanations directly linking to the clustering of the PCA

We fixed the manuscript and legend and made the flow better.

Lines 342-345:

'Mass spectrometry–based lipidomics of T cells from eight donors revealed global alterations in lipid composition in individuals with atherosclerosis **(Fig. 2i)**. Notably, the ether:acyl phospholipid and phospholipid:lysophospholipid ratios were significantly altered **(Fig. 2j)**.'

- Figure 2g/h: It would be desirable to see a plot with not only ratios but abundances for the different lipid classes in healthy vs diseased cells.

We really wanted to do that, but traditional lipidomics approaches typically require on the order of 9 million cells per sample, whereas our biological samples (isolated PBMCs from patients and further negatively selected for T cells) were limited to a few thousand cells per donor. To overcome this limitation, we employed a recently

developed low-input lipidomics technique, which allows reliable measurements from such limited material. To ensure comparability across donors and conditions, we strictly controlled the number of cells used per sample and applied total ion current normalization to account for potential technical variability. Due to differences in ionization efficiency across lipid species inherent to this approach, absolute abundances cannot be directly compared between lipid classes. Therefore, presenting lipid ratios for each sample provides the most accurate and unbiased representation of relative lipid remodeling. This ratio approach ensures robust, reproducible insights while minimizing potential artifacts arising from differential ionization or low-input variability.

- There is a mention in the text that the authors have seen "strong correlation between theoretically calculated membrane potential and the ratiometric fluorescence signal validating CV1 as a reporter for membrane potential in non-excitable cells" However, there is no corresponding figure to support this finding.

We referred to the figure in the manuscript (x-axis shows the theoretically calculated values and y-axis shows the experimentally measured values) and also make the legend clearer.

Lines 145-151:

'For membrane potential analysis, cells were modulated with valinomycin, a potassium ionophore enabling the selective transport of potassium ions (K^+) in combination with varying extracellular K^+/Na^+ ratios to calibrate CV1 responses according to the Nernst equation. We observed a strong correlation between the theoretically calculated membrane potential (**shown in x-axis**) and the ratiometric fluorescence signal, validating CV1 as a reporter of membrane potential in non-excitable cells **(Fig.1h, Supplementary fig.1-8)**.'

- Figure 2 i: it is unclear where these genes come from and where the raw data is, since there is none shown or referenced.

We have clarified the figure legend and manuscript text to improve the flow and explicitly indicate the source of the data, **Fig. 2k, l.**

- It is not clearly explained why the authors treat the PBMCs with sphingomyelinase

We added our motivation.

Lines 243-249:

"We used sphingomyelinase as a model drug that targets sphingomyelin which is one of the main lipids regulating the membrane integrity and order13. It catalyzes the hydrolysis of sphingomyelin to ceramide, which is known to induce changes in membrane packing. Recent cell type specific lipidomics analysis showed different levels of sphingomyelin in different immune cell types14. Therefore, we treated PBMCs with sphingomyelinase to test how modulating membrane sphingomyelin content alters biophysical properties in a cell type-dependent manner."

- Regarding the scRNA data, the sequencing quality control data missing from supplement

We added the 10x scRNA sequencing quality control as supplementary info. QC in analysis is described in the method section in detail. In short, the classic Seurat pipeline was used.

We thank the reviewers for their critical feedback. We apologize for misunderstanding the concerns of Reviewer 1 in the previous round. We believe the new data we added addresses these concerns.

Reviewer #1:

Comments 1 and 3 have not been sufficiently addressed in the authors' response. The explanations provided do not resolve my concerns, and the manuscript requires further revision to improve clarity and rigor.

We really appreciate the reviewer for the continued and constructive engagement with this point. Upon careful re-reading of the second revision comment, we realized that we misinterpreted the reviewer's comments in the first revision, and we recognize that our previous response did not adequately address two distinct concerns raised by the reviewer:

1. The analytical validity of comparing individual patient values to the mean of the healthy group.
2. The need for a formal statistical test demonstrating that cell-type-dependent differences in the disease effect are statistically significant, not merely a visual impression.

We apologize for this oversight. We have now fully addressed both concerns with new statistical analyses and figures described in detail below. Importantly, we have restructured our analytical approach to ensure that each statistical test is matched to the specific question it is designed to answer.

1. Regarding the authors' response to my comment, "Even if the biophysical properties are subtype specific, the change of these properties may not be.", I assume that the Fig. 1m stated in the following comments corresponds to Fig. 2f-g.

"…In addition, we added the statistics and delta values (mean difference) for representing the difference in biophysical properties between conditions (Fig. 1m), which is discussed in the next point below."

"…To this end, we added "delta membrane order" values on the graphs (Fig. 1m) for each cell type, so that it is immediately clear to the reader that different cell types are affected differently by drug treatment."

I do not see how these two sub-figures provide additional support for the "cell type-dependent" claim. **Firstly, it is not analytically meaningful to compare each patient's data to the mean of healthy data. Secondly, the variation in the difference of membrane order and depolarization between patients appears visually more significant than that between cell types (i.e., variation along rows vs. variation along columns).** To validate whether cell–type–dependent differences truly exist, a proper statistical analysis should be performed to determine whether the observations are statistically significant.

Concern 1: The mean-subtracted representation is analytically inadequate

***Reviewer:** "It is not analytically meaningful to compare each patient's data to the mean of healthy data."*

The reviewer is entirely correct. This representation in the previous Fig. 2f–g subtracted each patient's value from the healthy group mean, treating the healthy cohort as a single fixed reference constant. This approach has three fundamental problems: (i) it discards the variance within the healthy distribution, (ii) it provides no confidence interval for the reference, and (iii) it yields no inferential framework, so one cannot determine whether an observed difference is statistically meaningful because the uncertainty of the reference itself is ignored.

What we changed: We have **removed the mean-subtraction representation** entirely and implemented two complementary changes:

- All visualization panels (Fig. 2f–g) that previously used mean-subtracted representation have been replaced with pooled Z-score normalization **(new Fig. 2f–g)**. Z-score normalization computed per cell type across the pooled cohort (healthy and atherosclerosis combined), so no group serves as a fixed reference. Importantly, Z-score normalization preserves inter-individual variability within both groups, unlike mean subtraction which effectively collapses the healthy reference to a single point.

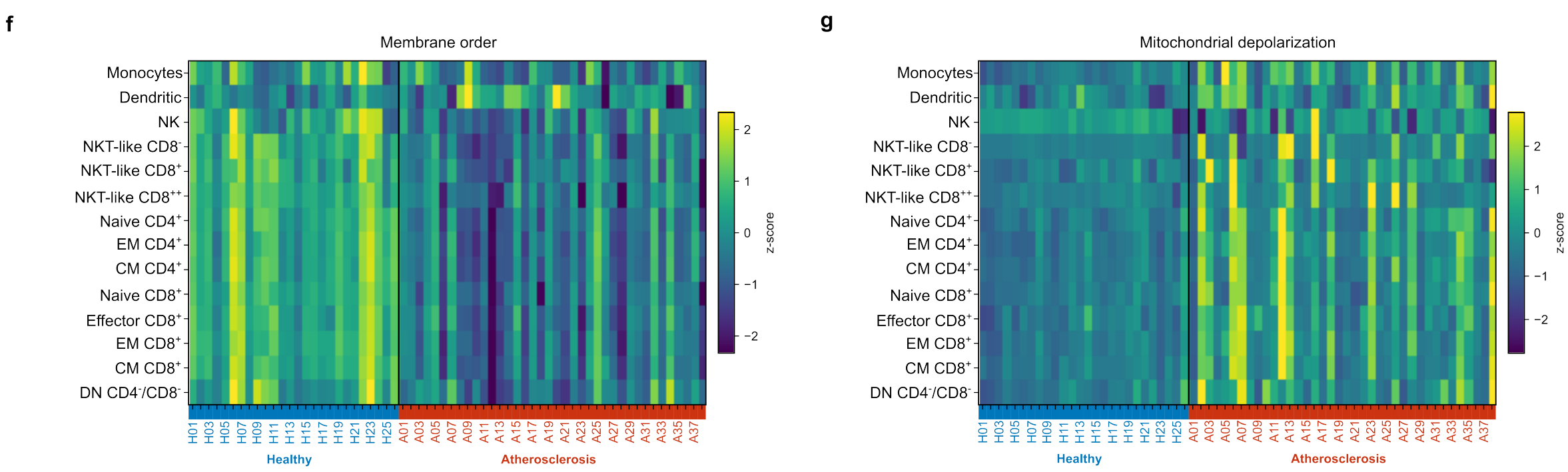

- All group comparisons (Healthy vs. Atherosclerosis) are now performed using **a two-way mixed ANOVA** (described in detail under Concern 2), which compares the full distributions of both groups within a single inferential framework. No individual patient is compared to a fixed mean; instead, the two groups are compared using their complete distributions including means, standard deviations, and sample sizes (**New Supplementary Fig. 2-2a and c**).

a

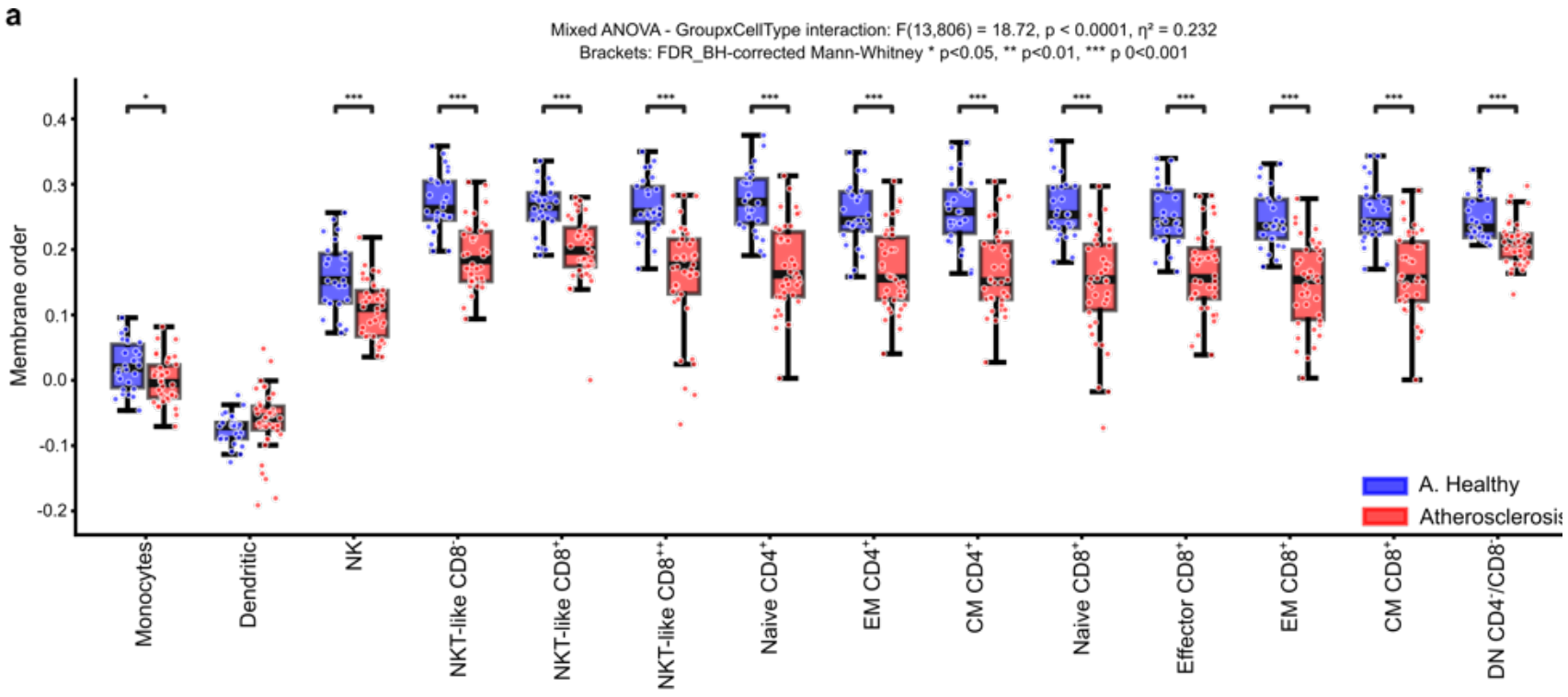

c

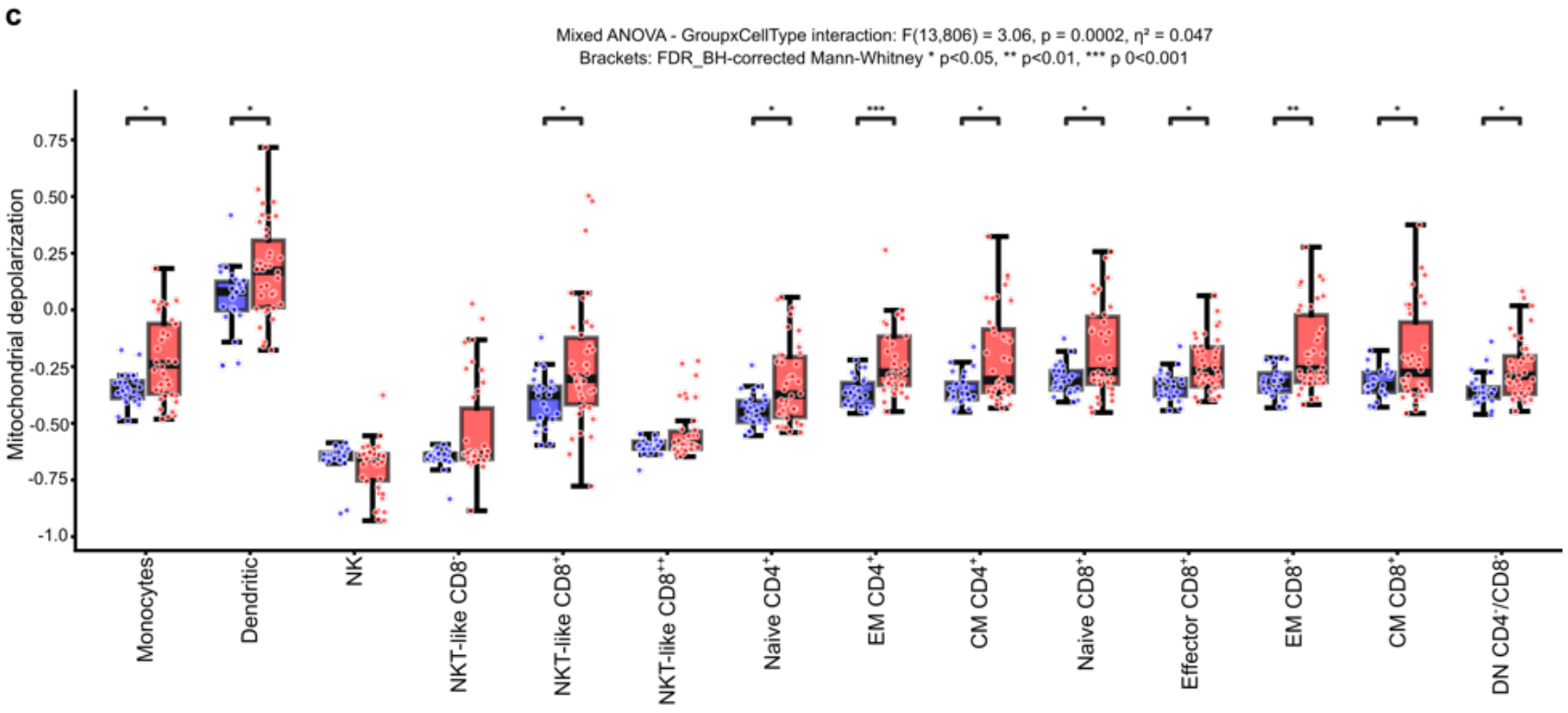

Together, these changes directly resolve the reviewer's concern: the reference group is never reduced to a fixed constant, and all statistical comparisons are performed on complete distributions within a rigorous inferential framework as detailed below.

Concern 2: Is the disease effect truly cell-type-dependent?

Reviewer: *"The variation in the difference of membrane order and depolarization between patients appears visually more significant than that between cell types (i.e., variation along rows vs. variation along columns). To validate whether cell-type-dependent differences truly exist, a proper statistical analysis should be performed to determine whether the observations are statistically significant."*

We fully agree that a formal statistical test is required, and we have now performed exactly the analysis the reviewer requested. Our response is organized in two parts: first, the **primary analysis** that directly answers the reviewer's question about the disease effect, and second, a **supporting baseline analysis** that establishes the biological context.

Primary analysis: Two-way mixed ANOVA (Healthy vs. Atherosclerosis)

The reviewer's core question is: *does the magnitude of disease-associated biophysical change truly differ across cell types?* This is a question about an **interaction effect**, specifically, whether the group difference (Healthy vs. Atherosclerosis) depends on which cell type is being examined.

Statistical model: We applied **a two-way mixed ANOVA** with group (Healthy, n = 26; Atherosclerosis, n = 38) as "between-subject" factor and cell type (14 levels) as "within-subject" factor. This design is the appropriate test for this question because:

- It uses the full distributions of both groups (not a single fixed reference), directly addressing Concern 1.
- It models the within-subject structure correctly (each individual is measured across all 14 cell types).
- It yields a Group × Cell Type interaction F-statistic that directly and formally tests whether the disease effect is the same across all cell types. **A significant interaction means that the disease effect is cell-type-dependent; a non-significant interaction would mean the effect is uniform.**

Results for membrane order (Supplementary Fig. 2-2a-b):

Group effect:	$F(1, 62) = 42.46$, $\mathbf{p < 0.0001}$, partial $\eta^2 = 0.406$
Cell-type effect:	$F(13, 806) = 462.44$, $\mathbf{p < 0.0001}$, partial $\eta^2 = 0.882$
Group × Cell Type:	$F(13, 806) = 18.72$, $\mathbf{p < 0.0001}$, partial $\eta^2 = 0.232$

Per-cell-type Mann–Whitney U (BH-corrected): 13/14 cell types significant

Results for mitochondrial depolarization (Supplementary Fig. 2-2c-d):

Group effect:	$F(1, 62) = 15.85$, $\mathbf{p = 0.0002}$, partial $\eta^2 = 0.204$
Cell-type effect:	$F(13, 806) = 200.93$, $\mathbf{p < 0.0001}$, partial $\eta^2 = 0.764$
Group × Cell Type:	$F(13, 806) = 3.06$, $\mathbf{p = 0.0002}$, partial $\eta^2 = 0.047$

Per-cell-type Mann–Whitney U (BH-corrected): 11/14 cell types significant

Interpretation of the interaction terms: The Group × Cell Type interaction is highly significant for both parameters (membrane order: $p < 0.0001$; mitochondrial depolarization: $p = 0.0002$). **This is the formal statistical proof that the disease-associated biophysical changes are not uniform across cell types; they are cell-type-dependent. In practical terms, this means that atherosclerosis does not alter membrane order or mitochondrial depolarization by the same amount in every cell type; some cell types are affected much more than others.**

The interaction effect size is substantially larger for membrane order (partial $\eta^2 = 0.232$, a medium-to-large effect) than for mitochondrial depolarization (partial $\eta^2 = 0.047$, a small but statistically significant effect). This may indicate that the cell-type specificity of disease-associated remodeling is particularly pronounced at the level of plasma membrane organization.

Which cell types drive the interaction? Because the interaction is significant, the overall group effect cannot be interpreted uniformly, so the logical next step is to examine per-cell-type comparisons. We performed Mann–Whitney U tests for each cell type separately, corrected for multiple testing using the Benjamini–Hochberg

procedure (FDR, α = 0.05), with effect sizes reported as Hedges' g ± 95% CI. Higher effect size means better discrimination between different groups.

Membrane order, effect sizes by cell type (Hedges' g):

Naive $CD4^+$:	g = 1.68	p_adj < 0.001	***
EM $CD8^+$:	g = 1.67	p_adj < 0.001	***
NKT-like $CD8^-$:	g = 1.66	p_adj < 0.001	***
CM $CD8^+$:	g = 1.63	p_adj < 0.001	***
Naive $CD8^+$:	g = 1.62	p_adj < 0.001	***
CM $CD4^+$:	g = 1.56	p_adj < 0.001	***
Effector $CD8^+$:	g = 1.50	p_adj < 0.001	***
EM $CD4^+$:	g = 1.48	p_adj < 0.001	***
NKT-like $CD8^{++}$:	g = 1.45	p_adj < 0.001	***
NKT-like $CD8^+$:	g = 1.34	p_adj < 0.001	***
NK:	g = 1.09	p_adj < 0.001	***
DN $CD4^-/CD8^-$:	g = 1.05	p_adj < 0.001	***
Monocytes:	g = 0.64	p_adj = 0.018	*
Dendritic:	g = −0.30	p_adj = 0.059	n.s.

Mitochondrial depolarization — effect sizes by cell type (**Hedges' g**):

EM $CD4^+$:	g = −1.11	p_adj < 0.001	***
EM $CD8^+$:	g = −0.95	p_adj = 0.005	**
Effector $CD8^+$:	g = −0.89	p_adj = 0.012	*
CM $CD4^+$:	g = −0.87	p_adj = 0.012	*
Monocytes:	g = −0.87	p_adj = 0.018	*
Naive $CD4^+$:	g = −0.81	p_adj = 0.020	*
DN $CD4^-/CD8^-$:	g = −0.79	p_adj = 0.012	*
Naive $CD8^+$:	g = −0.79	p_adj = 0.029	*
CM $CD8^+$:	g = −0.73	p_adj = 0.037	*
NKT-like $CD8^-$:	g = −0.69	p_adj = 0.087	n.s.
NKT-like $CD8^+$:	g = −0.67	p_adj = 0.018	*
Dendritic:	g = −0.64	p_adj = 0.032	*

NKT-like $CD8^{++}$: g = −0.63 p_adj = 0.221 n.s.

NK: g = 0.34 p_adj = 0.192 n.s.

Interpretation: The per-cell-type comparisons reveal a clear gradient of disease effects. For membrane order, T cell subsets, particularly $CD4^{+}$ and $CD8^{+}$ subsets show the largest effect sizes (**Hedges' g ranging from 1.34 to 1.68**), indicating large and robust differences between healthy donors and atherosclerosis patients hence making these cell types better discriminators between healthy and atherosclerosis groups. In contrast, Monocytes show only a moderate effect (g = 0.64) and Dendritic cells show no significant difference (p_adj = 0.059). For mitochondrial depolarization, a similar gradient is observed, with EM $CD4^{+}$ showing the strongest effect (**g = −1.11**) and NK cells showing no significant change (p_adj = 0.192).

This range of effect sizes, from non-significant to very large, is itself the hallmark of cell-type dependence. If the disease effect were uniform across all cell types, all effect sizes would be comparable, and the interaction term would not be significant. The fact that the interaction is significant, and the effect sizes span a wide range confirms that atherosclerosis differentially remodels biophysical properties in a cell-type-dependent manner.

We presented this data in Supplementary Figure 2-2b and d together with the previous data in Supplementary Figure 2-2a and c.

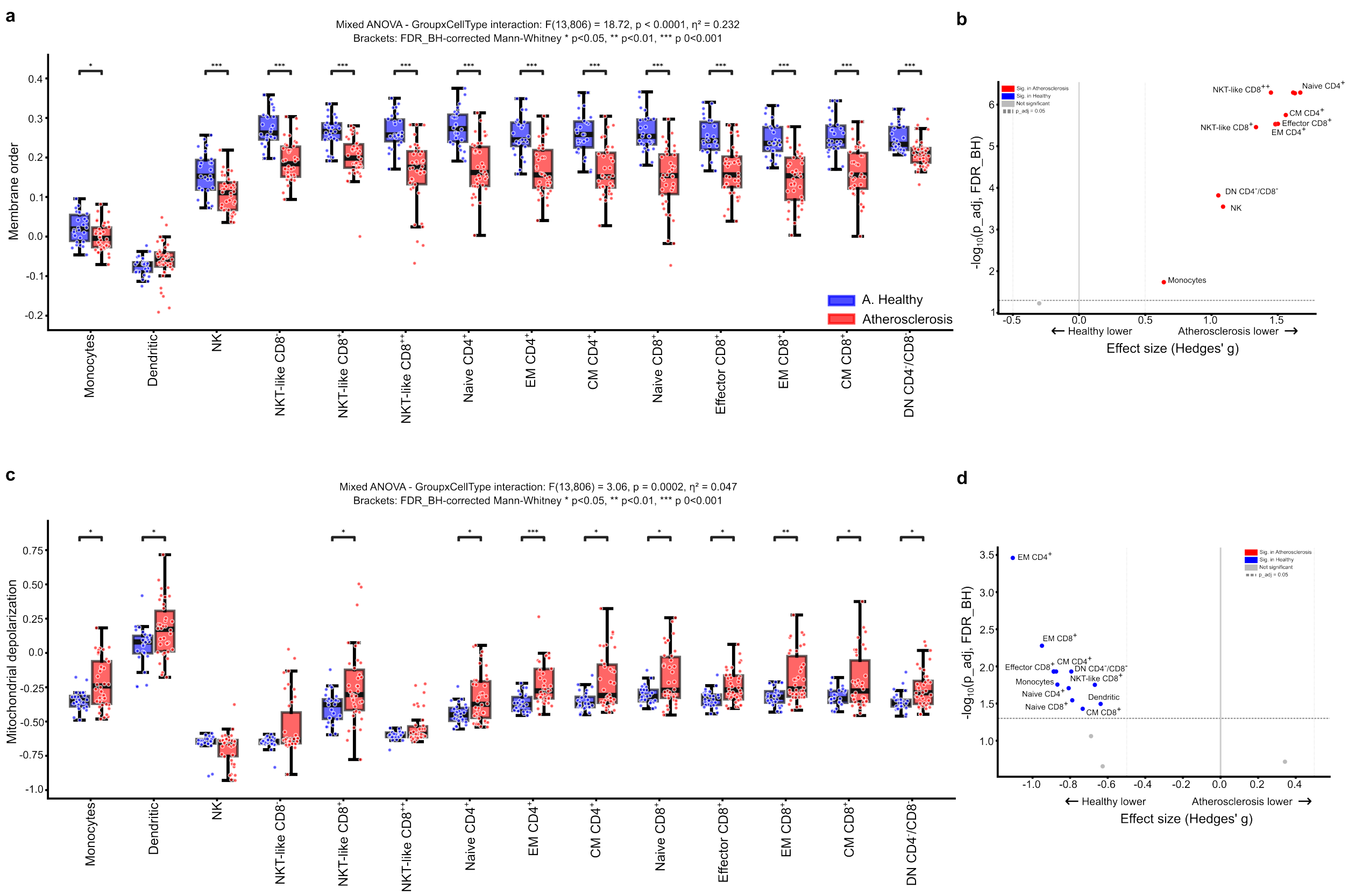

Supporting baseline analysis: Cell-type differences in healthy donors

While the mixed ANOVA above directly answers the reviewer's question about the disease effect, we also performed a complementary analysis on the healthy cohort alone to establish the biological foundation: that biophysical properties are intrinsically cell-type-dependent even at baseline, before any disease comparison is made.

Statistical model: Repeated-measures one-way ANOVA with cell type as the within-subject factor ($n = 26$ donors × 14 cell types). This model divides **total variance into three components: cell type, subject, and residual**. Therefore, one can directly assess what aspect is the stronger contributor to the overall variance of the system, e.g., differences in cell types or differences in donors. Greenhouse–Geisser correction was applied due to violation of sphericity (Mauchly's test $p < 0.0001$ for both parameters).

Results for membrane order

$F(13, 325) = 464.34$, $p < 0.0001$, partial $\eta^2 = 0.949$

**Cell-type variance:**	**84.8% of total**
Subject variance:	**10.6% of total**
Residual:	**4.6%**

Post-hoc: 67/91 pairwise comparisons significant (paired t-tests, BH-corrected)

Results for mitochondrial depolarization

$F(13, 325) = 181.91$, $p < 0.0001$, partial $\eta^2 = 0.879$

Cell-type variance:	**83.4% of total**
Subject variance:	**5.2% of total**
Residual:	**11.4%**

Post-hoc: 71/91 pairwise comparisons significant (paired t-tests, BH-corrected)

Why this matters for the disease comparison: This analysis establishes that the biophysical properties we measure are genuinely cell-type-dependent at the biological level; **cell-type identity explains ~83–85% of total variance**, **far exceeding inter-individual variability (~5–11%)**. This is important because it confirms that the biophysical readouts have **sufficient cell-type resolution and robust healthy-donor baseline** to detect differential disease effects in the first place. In other words, if the baseline values were not cell-type-specific, testing for cell-type-dependent disease effects would have limited biological meaning. The very large partial η^2 values (0.949 and 0.879) and the majority of pairwise comparisons being significant confirm that the readouts capture genuine biological differences between cell types.

We show these results in **New Supplementary Figure 2-1**. We are grateful for the reviewer’s insistence on this point, which has substantially strengthened the statistical rigor of our manuscript. We apologize again for misinterpreting the previous comments.

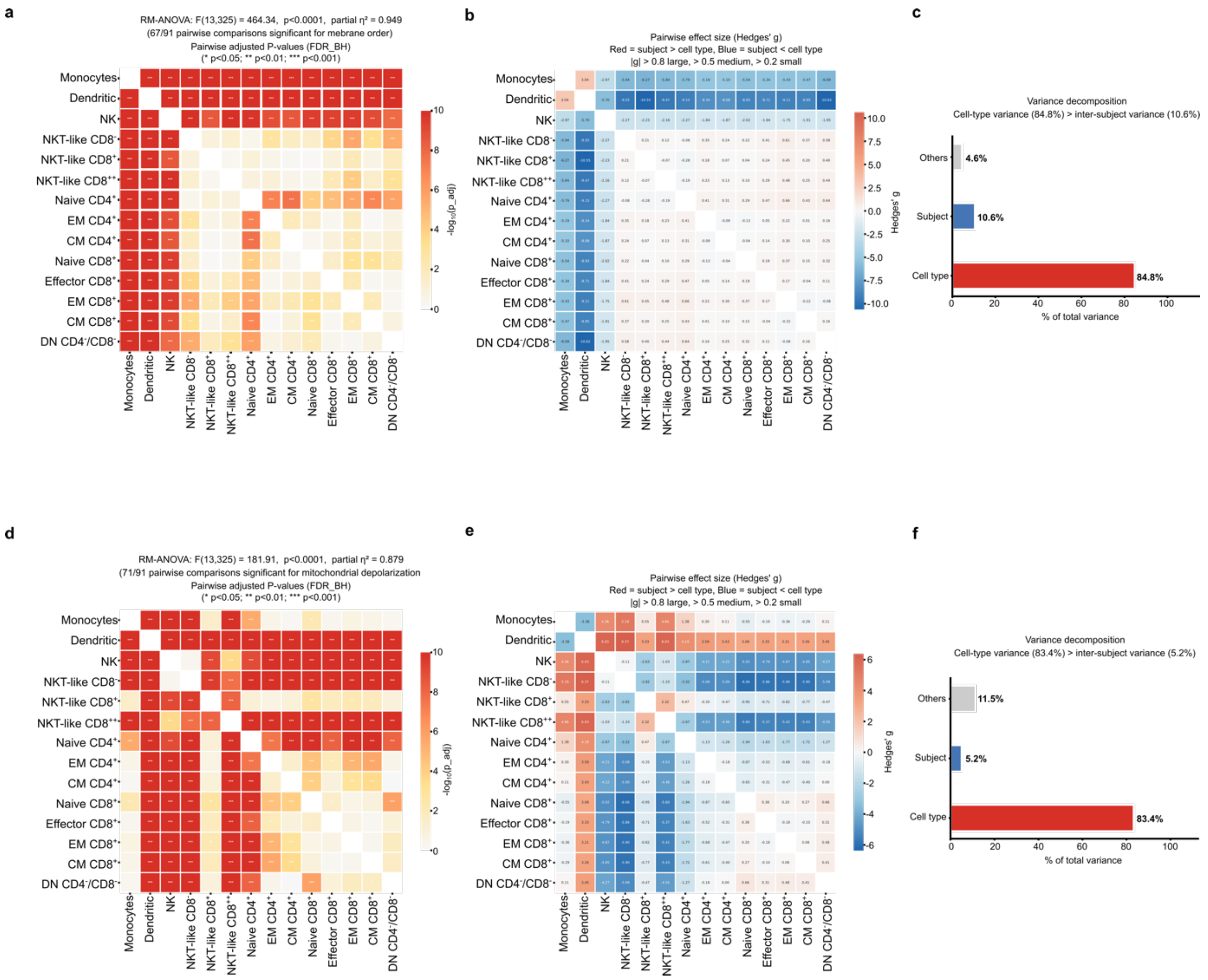

Modified manuscript:

Lines 279-300:

Before comparing conditions, we characterized the baseline biophysical heterogeneity across immune cell types within the healthy donor cohort **(Supplementary Fig. 2-1)**. Membrane order and mitochondrial depolarization both exhibited pronounced cell type-dependent differences, demonstrating that biophysical states are inherently heterogenous across the immune system rather than uniformly distributed. Pairwise comparisons confirmed that most cell types carry statistically distinct biophysical profiles with consistent effect sizes across donors **(Supplementary Fig. 2-1a–b, d–e)**. Critically, variance decomposition analysis showed that cell identity accounted for the large fraction of total variability in both membrane order (84.8%) and mitochondrial depolarization (83.4%), with inter-individual differences contributing substantially less (10.6% and 5.2%, respectively; **Supplementary Fig. 2-1c, f**). Together, these results establish a cell type-intrinsic baseline landscape of immune cell biophysics in healthy

individuals, providing a necessary reference for interpreting disease-associated alterations in a cell type-resolved manner. We then compared healthy donors and atherosclerotic individuals across all 14 immune cell types. Two-way mixed ANOVA revealed significant group and cell-type interactions for both membrane order and mitochondrial depolarization, confirming that disease-associated biophysical remodeling is cell-type-dependent rather than uniform across the immune compartment **(Supplementary Fig. 2-2)**. Per-cell-type comparisons identified T cells, including $CD4^+$ and $CD8^+$ subsets and their respective subtypes, as carrying the most pronounced biophysical changes in both parameters **(Supplementary Fig. 2-2a–d)**.

Lines 312-319:

To examine inter-individual variability better, we visualized donor-specific deviations in membrane order and mitochondrial depolarization across all 14 populations **(Fig. 2f, g)**. While consistent cell type-level trends were apparent, the magnitude and pattern of remodeling varied between individuals, revealing distinct donor-specific biophysical signatures and remodeling. Together, these findings highlight the selective and personalized nature of disease-associated biophysical remodeling and underscore the value of SBC in uncovering both shared and individual-specific immune biomarkers.

2. The specificity and effectiveness of membrane order as a viability marker must be vigorously validated to substantiate the claims in the manuscript.

Line 100-101, "…Notably, we found that Pro12A is also effective for distinguishing live and dead cells…."

It is clear that the membrane order is significantly influenced by various other parameters, as shown in Figs. 1and 2, spanning a broad range from -0.6 to 1. Notably, the membrane order shown in the Supplementary Fig. 1-3 falls well within the range – indicating NO absolute threshold that reliably identifies dead cells within a heterogeneous population, especially in complex clinical samples. Hence, a quantitative and rigorous analysis should be conducted to compare with the live/dead stain to demonstrate its validity for the blood sample.

We appreciate the reviewer's continued scrutiny. To address their concern, we added new data showing the robustness of Pro12A as cell death marker (**New Supplementary Fig. 1-3**). However, before explaining the results shown in this figure, we must respectfully clarify two fundamental misinterpretations.

1-) In the manuscript, we always used Live/Dead staining together with Pro12A (except one experiment with CV-1 in cell lines - not in clinical samples – as CV1 spectrally overlaps with the Live/Dead staining). Moreover, in all experiments, scattering of the cells was used to assess the cell viability in addition to these probes. Therefore, our observation of Pro12A being a robust cell death marker is a side-point in the manuscript which could easily be removed without affecting conclusions of the manuscript. However, we believe this is an important point because Live/Dead staining makes flow cytometry measurements more challenging (explained later).

2-) The broad range of membrane order values (−0.6 to 1) observed across the manuscript does not reflect variability within a single experimental condition. This range spans measurements obtained across entirely distinct experimental contexts, synthetic lipid bead standards with very disordered membranes, cell lines, and primary PBMCs, each acquired with different probe combinations and spectral channel configurations, as explicitly detailed in the Methods (SBC measurements and analysis). **Within each defined experimental context**, the separation between live and dead cells is consistent, pronounced, and unambiguous. Specifically, for PBMCs measured with the Pro12A + JC-1 combination (the configuration used for all clinical data in the paper), live cells consistently showed membrane order values in minimum range of −0.1/−0.2, while dead cells shift reproducibly to approximately −0.4 to −0.5. This represents a large, biologically driven separation that reflects disrupted membrane integrity and probe internalisation into the aqueous intracellular environment.

New experimental data: Despite these two points, we agree with the reviewer that we needed to show that Pro12A is a robust cell death marker. To address the reviewer's request for a quantitative and rigorous validation, we have expanded Supplementary Figure 1-3 with the following analyses:

- Cell lines (3 replicates): Live and dead populations were gated independently using (i) conventional live/dead staining and (ii) membrane order threshold. Statistical comparison found no significant difference between the live populations identified by each method, nor between the dead populations (Supplementary Figure 1-3d), confirming equivalence of these two gating strategies, hence the probes.
- Primary PBMCs (3 replicates): The same paired analysis was performed separately in lymphocyte and monocyte gates. No significant difference was observed between live/dead staining and membrane order-based gating in either population (Supplementary Figure 1-3g and j).

We believe, these results demonstrate that membrane order provides equivalent discriminatory power to conventional viability dyes within the relevant measurement context, with full quantitative support across both cell lines and primary clinical samples.

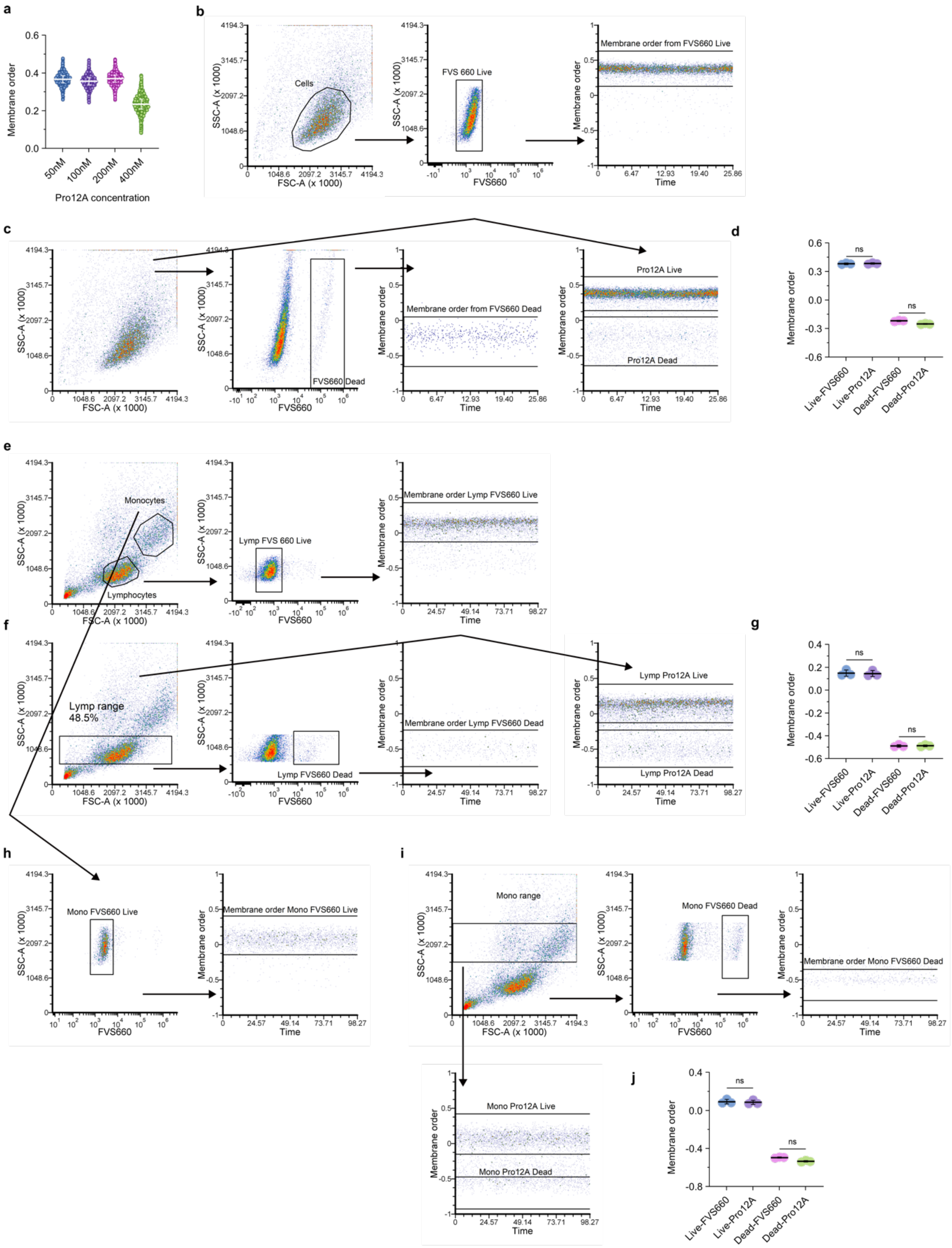

Why we think it is important to include this data: As stated earlier, we can easily remove this statement without compromising any of the biological conclusions in the manuscript. However, beyond equivalence in performance, we are confident that our probe offers meaningful advantages over conventional amine-reactive live/dead dyes that are worth highlighting for the community:

- Temporal resolution: The membrane order probe is applied immediately before acquisition. Conventional live/dead dyes require 15–30 minutes of staining plus pre- and post-washes. During this interval, cells that were viable at the time of staining can undergo death and will not be detected by the dye. The membrane order probe captures this in real time, as dead cells display an immediate and pronounced shift toward lower values reflecting membrane disruption and probe internalisation.
- Reduced cell loss: Additional wash steps associated with live/dead staining reduce total cell yield, which is particularly problematic for rare primary cell subsets in clinical PBMC samples. Eliminating these steps preserves sample integrity.
- No additional staining step: Integration of viability assessment into the membrane order measurement itself simplifies the experimental workflow without sacrificing sensitivity or specificity.

For these reasons, we think that community can get benefit from the probe to identify live cells considering the advantages mentioned.

Modified manuscript:

Lines 104-123:

Notably, we found that Pro12A is also reliable and real-time readout of cell viability that is equivalent to conventional Live/Dead staining **(Supplementary fig.1-3b–j)**, without requiring additional staining or wash steps. In dead cells, Pro12A is rapidly internalized and reports low membrane order, reflecting the disordered nature of intracellular organelles. Because the probe is applied immediately before acquisition, it detects cells that undergo death during the interval between conventional viability staining and cytometric measurement, a limitation inherent to amine-reactive dyes. This integrated viability assessment could reduce cell loss during sample preparation, simplifies the experimental workflow, and minimizes viability-related artefacts in time-sensitive sample measurements. This suggests that Pro12A could potentially obviate traditional viability dyes during biophysical measurements, which is especially advantageous when working with multiple probes and limited spectral bandwidth. Nevertheless, to ensure robustness, we included conventional viability staining alongside membrane order probe in all experiments, except in measurements using membrane potential dye due to its broad red spectrum. In these cases, the combination of membrane order and light scatter (since dead cells display distinct size distributions) provided a reliable means of discriminating live from dead cells. Importantly, all experiments were performed under optimized culturing conditions, and our analyses of blood samples consistently started with the highest possible viability.

Reviewer #2

I would like to thank the authors for addressing all previously raised points and for providing the additionally requested data.

Minor Comments

• In the introduction, several sentences - particularly from line 72 onward - are very long and could be made more concise. Furthermore, after "(Fig. 1a)." the full stop appears to be incorrectly placed.

Fixed.

Lines 76-79:

'First, to demonstrate the sensitivity and robustness of the pipeline with low complexity, we utilized a model membrane system, called bead-supported lipid bilayers (BSLBs), by coating cell-sized 5 μm silica beads with synthetic liposomes **(Fig. 1a)**.'

• Regarding the suggestion for coherent colors: using an inclusive color palette is appreciated. However, colors should still remain coherent. For example, in Fig. 1e the color used for the 18:1–18:1 lipid should match the pink used for this composition in Fig. 1b and remain distinct from the purple used for 16:1–16:1. The same applies to Fig. 1c. Keeping colors consistent across panels for the same lipid composition makes the figures easier to follow.

We changed all colors which are coherent now as suggested by the reviewer, both in **Fig.1b-e** and **Supplementary fig.1-2**.

• Figure 1e, the main text, and Supplementary Figures 1–2e: the wording "Lipid tails length" should be revised. It should either be "tail length" or "tail's length," but ideally it should be replaced with "acyl chain length."

Acyl chain length is chosen as suggested by the reviewer.

• Supplementary Figures 1–2: thank you for adding the arrow to highlight the difference of interest. However, it remains unclear what the essential information is e.g., is it the differential y-value of differently ordered compositions? Additionally, while the colors between panels a and d are consistent, the colors between panels d and e are not. Improving color coherence across panels would help the reader.

Explained better in the legend and colors fixed.

'**(d)** Normalized emission spectra across all lipid compositions. Arrow shows increased relative emission at longer wavelengths corresponds to a red shift associated with lower membrane order.'

• Supplementary Figure 1–3: statistical information is missing. Displaying non-significant changes as well would strengthen the point being made.

Fixed.

• Figure 2c & d (left) and corresponding text around line 280: the use of the term "clear" may be overstated given that the groups partially overlap.

Fixed.

Lines 300-303:

‘To visualize inter-individual differences, we plotted median membrane order and mitochondrial depolarization for Naive $CD4^{+}$ and Effector $CD8^{+}$ T cells in a 2D feature space, indicating a shift in the distribution between healthy and atherosclerotic individuals **(Fig. 2c, d, left)**.’

• Figure 2h (in relation to f & g): outlining the further-analyzed samples greatly improved clarity - thank you for adding this. It may also be helpful to indicate which specific patient (patient numbers as in panels f & g) each highlighted data point corresponds to, especially now that an overall trend per patient is visible in the heatmaps.

Both healthy and patient numbers are added.

• A further thought on Fig. 2h: selecting the four samples per condition that are most clearly separated is entirely reasonable. Still, it could be interesting to explore whether lipidomics or transcriptomics alone can discriminate the overlapping areas. This does not need to be addressed; it is more of a conceptual suggestion.

We thank the reviewer for this excellent question. We would like to explore this in the future with a more budget-friendly approach to chosen cell types.

• Another consideration regarding Fig. 2c–k: if available, integrating physiological disease severity into the separation could be informative. Mapping disease severity onto the analysis space might reveal inherent trends, such as clearer separation with increasing severity, and may even allow visualization of disease progression.

We thank the reviewer for this great question. Unfortunately, we did not have access to systematic clinical information. We would like to explore this in the future with a bigger cohort integrated with clinical information.
